# Light-Induced Access to Carbazole-1,3-dicarbonitrile: A Thermally Activated Delayed Fluorescent (TADF) Photocatalyst for Cobalt-Mediated Allylations

**DOI:** 10.1021/acs.joc.2c01825

**Published:** 2022-11-16

**Authors:** Emanuele Pinosa, Elena Bassan, Sultan Cetin, Marco Villa, Simone Potenti, Francesco Calogero, Andrea Gualandi, Andrea Fermi, Paola Ceroni, Pier Giorgio Cozzi

**Affiliations:** † Alma Mater Studiorum - Università di Bologna, Dipartimento di Chimica “G. Ciamician”, Via Selmi 2, 40126 Bologna, Italy; ‡ Center for Chemical Catalysis - C3, Alma Mater Studiorum - Università di Bologna, Via Selmi 2, 40126 Bologna, Italy; § Scuola Normale Superiore, Piazza dei Cavalieri 7, 56126 Pisa, Italy

## Abstract

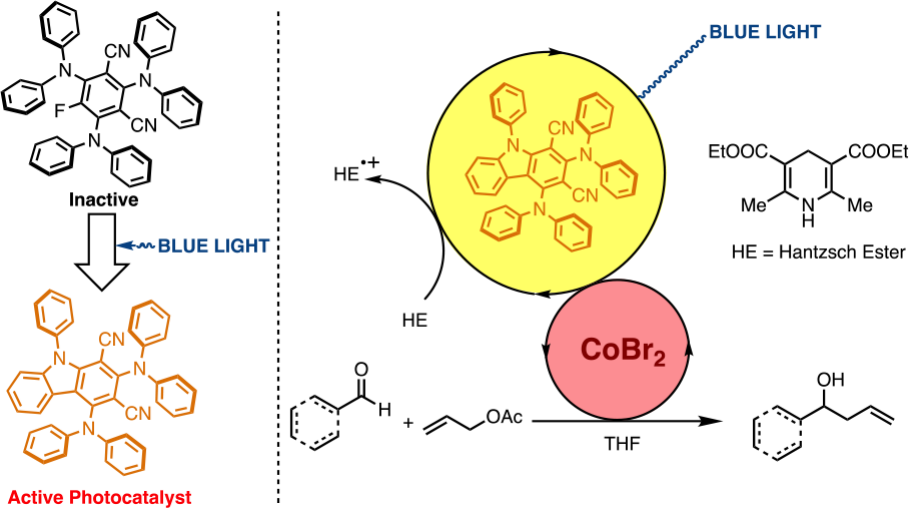

The stability of a photocatalyst under irradiation is important in photoredox applications. In this work, we investigated the stability of a thermally activated delayed fluorescence (TADF) photocatalyst {3DPAFIPN [2,4,6-tris(diphenylamino)-5-fluoroisophthalonitrile]}, recently employed in photoredox-mediated processes, discovering that in the absence of quenchers the chromophore is unstable and is efficiently converted by irradiation with visible light into another species based on the carbazole-1,3-dicarbonitrile moiety. The new species obtained is itself a TADF emitter and finds useful applications in photoredox transformations. At the excited state, it is a strong reductant and was efficiently applied to cobalt-mediated allylation of aldehydes, whereas other TADFs (4CzIPN and 3DPAFIPN) failed to promote efficient photocatalytic cycles.

## Introduction

Recently, photoredox catalysis has been exploited as a source of innovative methodologies in organic chemistry.^1^ Among the possibilities offered by photoredox catalysis, dual metal and photoredox catalysis,^2^ i.e., the combination of metal-promoted processes with photoredox cycles, is attracting more and more interest in academia and industry. For the development of new efficient and selective metal-promoted reactions, the use of inexpensive, readily synthesized, and efficient organic dyes represents a strategic topic in research.^3^ In this context, organic dyes need to compete with and replace widely employed inorganic complexes based on Ir(III) and Ru(II), which have long excited state lifetimes that can favor dynamic quenching with organic molecules. Normally, organic dyes have shorter excited state lifetimes, which is a major drawback for the design of efficient photoredox processes. Recently, a particular class of organic chromophores have attracted considerable attention for their interesting properties and efficiency.^4^ These molecules possess a property called thermally activated delayed fluorescence (TADF), which is displayed by molecules exhibiting a small energy gap (generally <0.2 eV) between the two lowest excited states, namely, S_1_ and T_1_. In these molecules, reverse intersystem crossing (RISC) from T_1_ back to S_1_ takes place at room temperature by a thermally activated process, yielding the so-called delayed fluorescence. The challenge is to couple the high efficiency of RISC to the high quantum yield of fluorescence. In 2012, Adachi published a seminal paper^5^ reporting carbazolyl dicyanobenzene molecules displaying the desired photophysical properties and demonstrated their applications in organic light-emitting diodes (OLEDs). Since then, similar TADF chromophores have been applied in a variety of different fields, including photocatalysis.^4,6^ By taking advantage of the easily tunable redox potentials and the long-lasting singlet excited states due to TADF, isophthalonitriles are suitable chromophores for exploitation as organic photocatalysts for a broad selection of chemical reactions.^7^ Specifically, 2,4,6-tris(diphenylamino)-5-fluoroisophthalonitrile (3DPAFIPN) has been used in recent years for a number of visible-light-fueled synthetic protocols, for instance, in intramolecular cyclizations^8,9^ and C–C,^10,11^ N–C,^12^ and P–C bond formation.^13^

3DPAFIPN is reported to be stable under the reaction conditions used for photocatalysis,^14,15^ as determined in experiments by some of us in the photoredox allylation of aldehydes by using either titanium^16,17^ or nickel^18^ in its low oxidation state. The photostability of 3DPAFIPN in those applications was demonstrated by its recovery at the end of the reaction, making its reuse possible. However, upon prolonged irradiation of a degassed THF solution of 3DPAFIPN, we observed the formation of a photoproduct, which prompted us to further investigate the photoreactivity of the former. The stability and reactivity of photoredox catalysts have recently been addressed, given their importance in defining the species that are genuinely involved in the photoredox processes. In some cases, upon photoirradiation, new photocatalysts with enhanced redox properties were formed in solution, allowing challenging transformations.^19^ We have also demonstrated that a two-photon process can be driven by the formation of a photoproduct originating from the starting photocatalyst.^20^

The photoreactivity of TADF chromophores like 3DPAFIPN has already been reported in the literature. For instance, König reported that, upon blue-light irradiation in the presence of phenylacetic acid, tetracarbazolyl derivative 2,4,5,6-tetrakis(9*H*-carbazol-9-yl) isophthalonitrile (4CzIPN) undergoes a photosubstitution reaction of a cyano group with a benzyl group.^21^ In general, irradiation of 4CzIPN in the presence of aliphatic carboxylic acids (R-COOH) produces the photosubstitution product in which one cyano is replaced by the alkylic group R.^22^ The resulting photoproducts display blue-shifted absorption and emission, and more negative reduction potentials. The authors demonstrated that the photosubstituted TADF chromophores are responsible for the observed photocatalytic reactions in many reported literature procedures. In our investigation, we considered a TADF chromophore (3DPAFIPN) that features not only cyano and diphenylamino groups but also a fluorine atom in the aromatic core. Fluorobenzenes are known to undergo photoreactions via homolytic cleavage of the C–F bond or photosubstitution by nucleophilic attack, generally proceeding via electron transfer processes.^23^ For example, the photoreaction of fluorobenzene with aliphatic amines yields the substitution product^24^ and addition products.^25,26^

**Figure 1:**
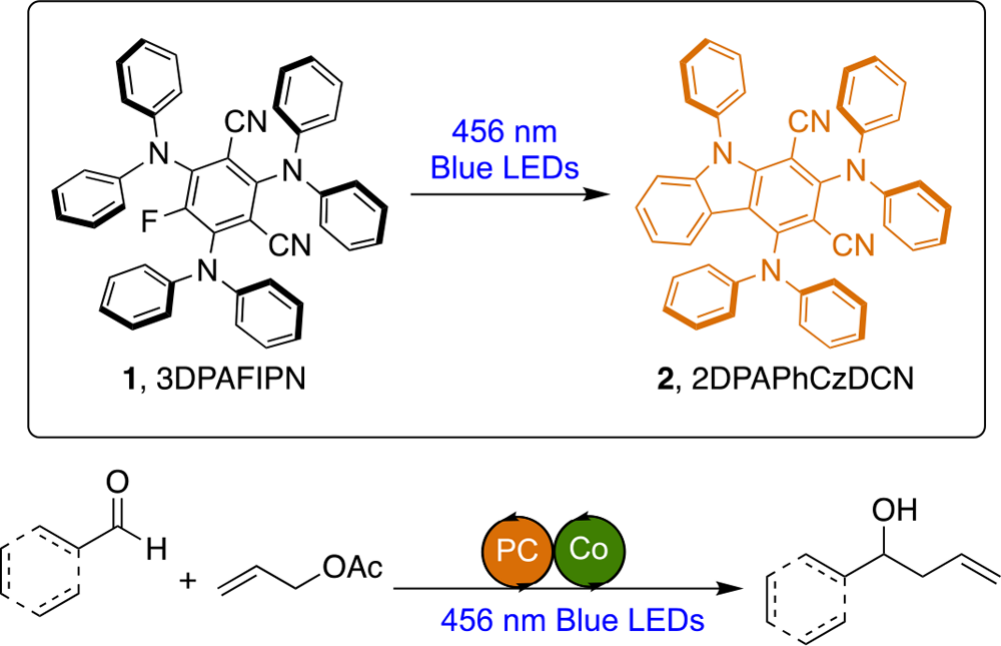
3DPAFIPN (**1**), the new photocatalyst 1,3-dicyanocarbazole (**2**), and cobalt-promoted allylation of aldehydes.

Here, we investigate the photoreaction of 3DPAFIPN (**1**) by isolating the photoproduct 2,4-bis(diphenylamino)-9-phenyl-9*H*-carbazole-1,3-dicarbonitrile [2DPAPhCzDCN, **2** (Figure 1)], and we compare the photophysical and electrochemical properties of the latter with those of the starting TADF chromophore. Because reduced compound **2**^•–^ is a stronger reductant [*E*(**2**/**2**^•–^) = −1.74 V vs SCE (vide infra)] compared to the parent species **1**^•–^ [*E*(**1**/**1**^•–^) = −1.53 V vs SCE (vide infra)], we examined the performance of **1** and **2** in the challenging allylation reaction of aldehydes mediated by cobalt with allyl acetate.^27^ We in fact report that the widely used and commercially available 4CzIPN^28^ was giving only traces of the desired homoallylic product.^29^ Herein, we report the full and detailed photophysical investigation of the new photocatalyst **2**(30) and its application in the cobalt-mediated allylation reaction (Figure 1).

## Results and Discussion

**Scheme 1:**
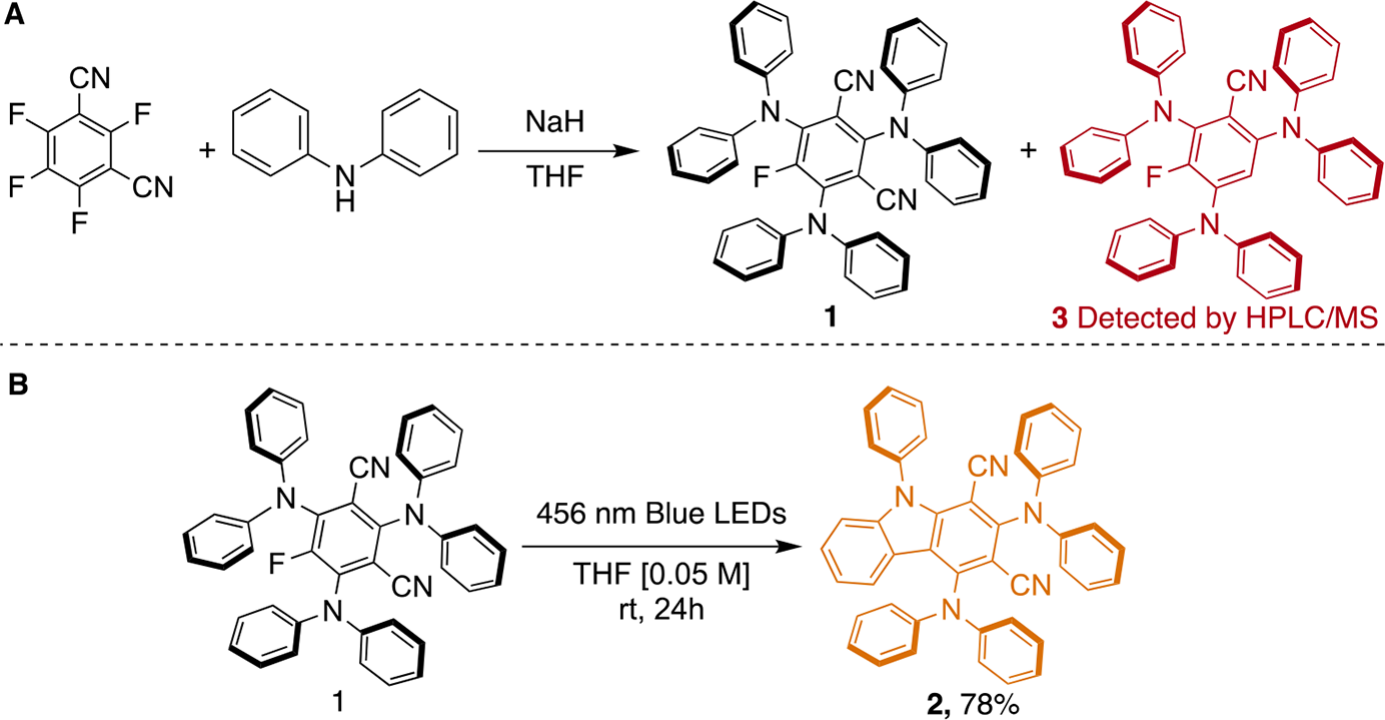
Synthesis of Dyes **1** (3DPAFIPN) and **2** (2DPAPhCzDCN)

During the photophysical investigations of photoredox reactions involving 3DPAFIPN (**1**) by irradiation with visible light in the absence of any quencher, we observed the formation of another product, which was isolated and fully characterized. By careful ^1^H and ^13^C NMR analysis, and application of several two-dimensional NMR techniques (see the Supporting Information for details), we were able to assign the structure of **2** to the newly formed compound. First, we checked if the starting material contained any impurity that could drive the photochemical transformation. By a careful HPLC-MS analysis, we discovered that the methodology reported for the preparation of **1**(7) led to the concomitant formation of traces of product **3**, namely the corresponding monocyano derivative (Scheme 1A). A challenging chromatographic purification was therefore needed to isolate a pure sample of **1**. Then, a THF solution of **1** was then irradiated using a blue Kessil lamp (456 nm). The photoreaction was scaled to 0.1 mmol, and irradiation for 24 h allowed the complete transformation to **2** (Scheme 1B; see the Supporting Information for details).

**Figure 2:**
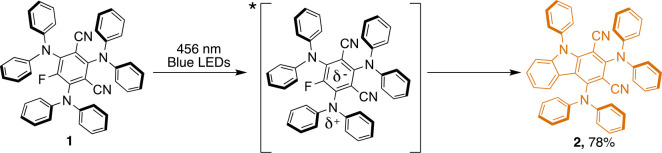
Charge transfer state involved in the formation of **2** by irradiation of **1** with a blue LED.

It is worth mentioning that the photoreaction was observed when carrying out the experiment in the absence and presence of oxygen and the use of different solvents (toluene, DMF, and MeCN) yielded similar results. HPLC-MS analysis showed that 2,4,5,6-tetrakis(diphenylamino) isophthalonitrile (4DPAIPN) does not undergo cyclization, as expected for a nonfluorinated compound. On the basis of the literature, three mechanisms can be envisioned for this class of fluorinated molecules, involving electron transfer, photonucleophilic substitution, or homolysis of the C–F bond.^31−34^ The charge transfer nature of the lowest excited state of **1** (Figure 2), with increased electronic density on the fluorinated aryl moiety, would assist a mechanism involving an electron transfer. However, photonucleophilic substitution is also plausible and discerning between these two mechanisms is difficult. The pathway that includes the homolytic cleavage of the C–F bond in **1** can be excluded on the basis of the insufficient energy of the absorbed photons [absorption onset at 480 nm = 60 kcal/mol (Figure 2)] compared to that of the C–F bond (127 kcal/mol).^35^

**1** and **2** were studied from photophysical and electrochemical points of view, to analyze and rationalize the effect of the cyclization on their electronic properties (Table 1).

**Table 1:** Photophysical and Electrochemical Properties (*E*_1/2_ in volts vs SCE) of **1** and **2** in THF at Room Temperature, Unless Otherwise Noted

	absorption		emission								electrochemistry	
	λ_ABS_^MAX^ (nm)	ε (M^–1^ cm^–1^)	λ_FLUO_^MAX^ (nm)	λ_PHOS_^MAX^ (nm)^a^	*τ*_FLUO_ (ns)	τ_TADF_ (μs)^b^	τ_PHOS_ (ms)^a^	Φ_FLUO_ (%)	Φ_TADF (%)_	Φ_Δ_ (%)	*E*(A^•+^/A) (V)^c^	*E*(A/A^**•**–^) (V)^c^
**1**	364	15700	510	518	3.3	130	180	5.7	35	88	+1.31^d^	-1.53
**2**	377	10500	479	513	9.1	680	374	21	6.2	55	**+1.31**	-1.74

a At 77K in a glassy matrix [1:1 (v/v) DCM/MeOH].

b Degassed solution.

c In MeCN.

d Anodic peak potential at 1 V/s, chemically irreversible electron transfer process.

**Figure 3:**
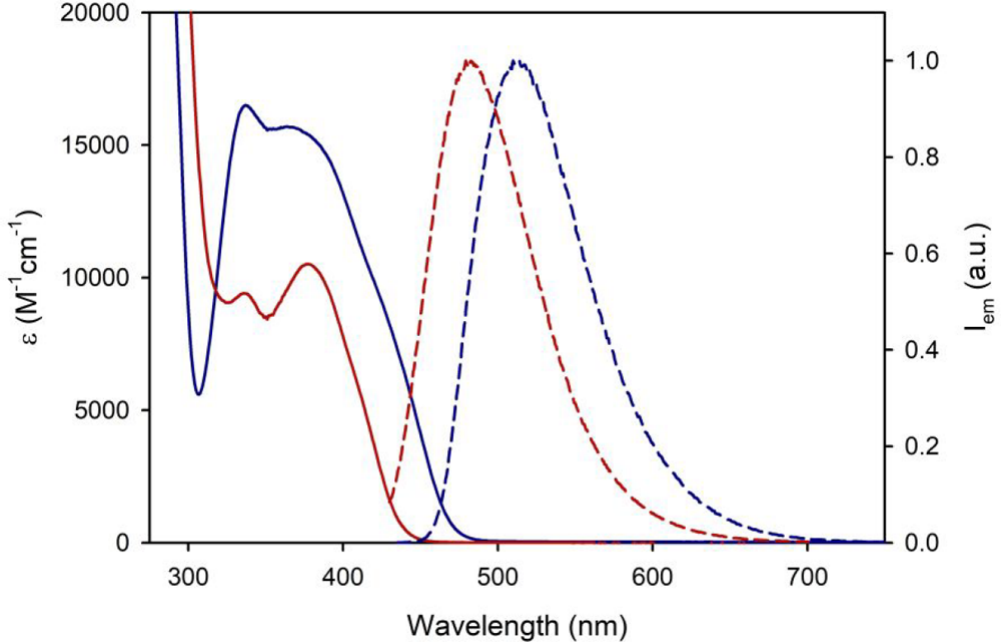
Absorption (solid line) and emission spectra (dashed line; λ_ex_ = 420 nm) of compounds **1** (blue) and **2** (red) in air-equilibrated THF.

The absorption spectrum of **2** appears to be blue-shifted compared to that of **1**, in terms of absorption onsets (Figure 3). The same trend is observed in the fluorescence spectra (λ_max_= 510 nm for **1** and 479 nm for **2**, in THF at rt) (Figure 3). In both compounds, two lifetimes are observed in degassed THF solutions at room temperature. The shorter component, in the range of nanoseconds, has been ascribed to prompt fluorescence (τ_PROMPT_), while the longer one, in the range of microseconds, has been attributed to TADF [τ_TADF_ (Table 1)]. In fact, the shape of the emission spectra for both compounds **1** and **2** is not affected by the presence of molecular oxygen, thus suggesting that the transition responsible for the longer lifetime is the same, namely the radiative deactivation S_1_ → S_0_. On the contrary, the emission quantum yield is decreased in air-equilibrated solutions due to efficient quenching of the chromophores’ T_1_ excited state by dioxygen, which consequently prevents the thermally activated RISC from undergoing the T_1_ to S_1_ step.

**Figure 4:**
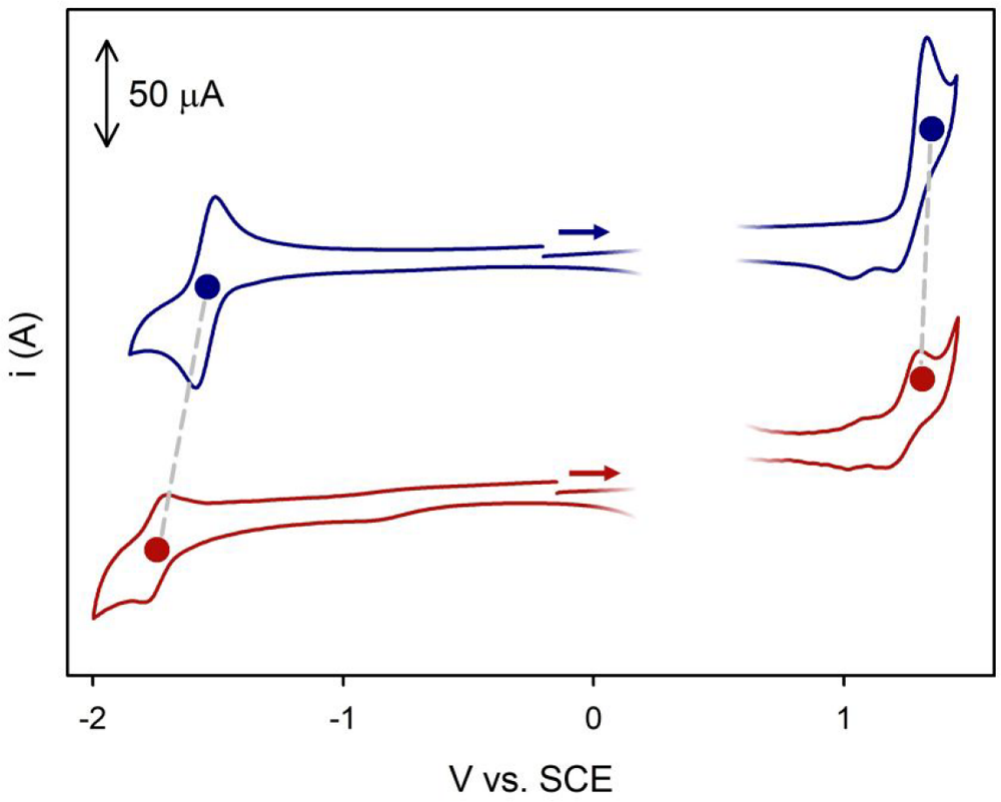
Cyclic voltammetry using the IUPAC convention of **1** (blue) and **2** (red) (0.5 mM) in a degassed MeCN solution containing 0.05 M tetraethylammonium hexafluorophosphate as the supporting electrolyte and ferrocene as the internal standard. Conditions: glassy carbon working electrode, Pt wire counter electrode, Ag wire quasi-reference electrode, scan rate of 1 V/s, rt. Ferrocene’s peaks have been omitted for the sake of clarity.

The quantum yield of prompt fluorescence (Φ_FLUO_) is enhanced from 5.7% to 21% upon passing from **1** to **2**, as expected for a cyclization that rigidifies the molecular structure. The rigidity of the new chromophore causes an increase in τ_PROMPT_, τ_TADF_, and τ_PHOS_, as well. Moreover, emission spectra recorded in a glassy matrix at 77 K evidence the presence of phosphorescence for both compounds [λ_max_ = 518 and 513 nm for **1** and **2**, respectively (Figure S1)]. Under these experimental conditions, the phosphorescence bands are slightly red-shifted compared to their fluorescence, indicating that S_1_ and T_1_ are close in energy. In particular, the S_1_–T_1_ energy gap (Δ*E*_ST_) is larger for compound **2** (320 meV) than for the pristine chromophore **1** [190 meV (Figure S1)]. We expect that the same trend is maintained at room temperature, proving the lower TADF quantum yield and longer τ_TADF_ for **2** than for **1**. As a rule of thumb, a high Δ*E*_ST_ is expected to lead to a low *k*_RISC_ because of the increased activation energy for the T_1_ → S_1_ intersystem crossing. Moreover, given the inverse proportionality between kinetic constants and lifetimes, a low *k*_RISC_ should concomitantly lead to a high τ_TADF_. However, this assumption cannot always be generalized because relatively small differences in Δ*E*_ST_ can result in great differences in *k*_RISC_, as reported for other classes of TADF-active chromophores.^36,37^ Cyclic voltammetry was carried out to determine the redox potentials of both species (Figure 4).

In the case of compound **1**, the reduction process (−1.53 V vs SCE) is chemically and electrochemically reversible while the oxidation process (+1.31 V vs SCE) shows only partial chemical reversibility at a scan rate of 1 V/s. Derivative **2** displays less chemically reversible electron transfer processes. While its oxidation potential is unchanged compared to that of the parent compound (+1.28 V vs SCE), its reduction potential is cathodically shifted to −1.74 V vs SCE. Taking into consideration the localized nature of frontier molecular orbitals in TADF molecules,^38^ the electrochemical data indicate that the LUMO orbital is destabilized in **2** compared to **1**, as expected upon removal of the fluorine substituent in the photoproduct. On the contrary, the HOMO orbital is not appreciably affected, as two electron-donating diphenylamine groups are also present in compound **2**. Ultimately, the larger energy gap between the HOMO and LUMO orbitals detected from electrochemical measurements of **2** is in accordance with the blue-shifted absorption and emission spectra of **2** (Figure 3).

**Scheme 2:**
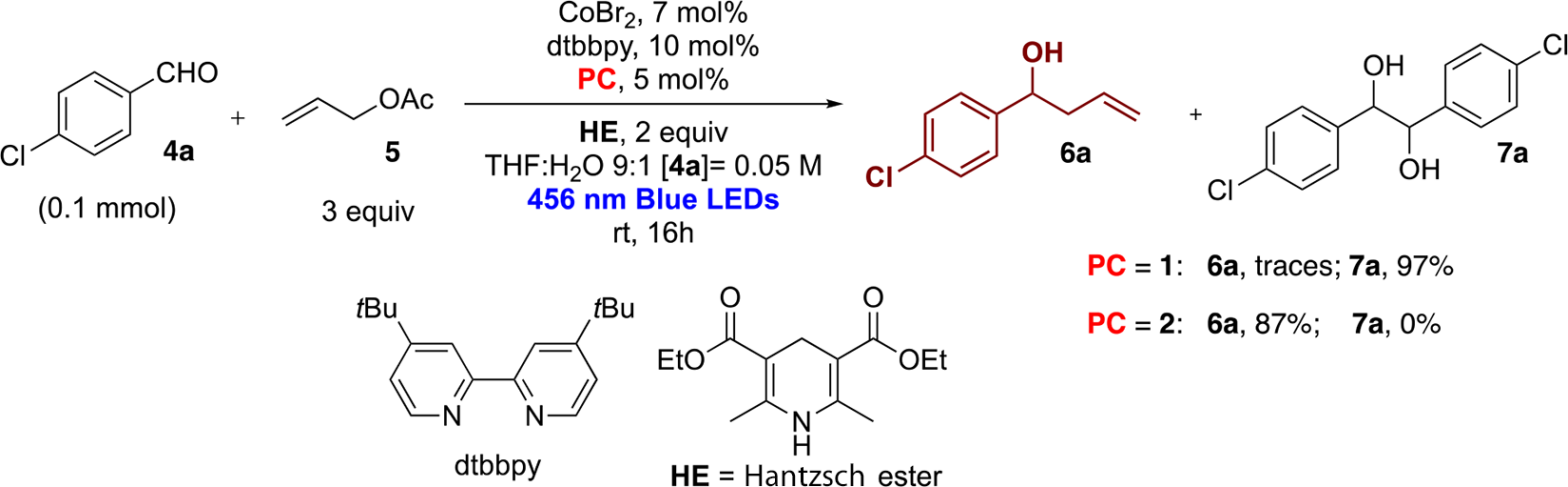
Allylation of 4-Chlorobenzaldehyde (**4a**) under Photoredox Conditions in the Presence of Photocatalysts **1** and **2**

Recently, we have reported a cobalt-mediated photoredox allylation reaction,^24^ in the presence of the abundant CoBr_2_ (10 mol %), 4,4′-di-*tert*-butyl-2,2′-dipyridyl (dtbbpy, 10 mol %), allyl acetate (3 equiv), [Ir(dtbbpy)(ppy)_2_]PF_6_ (ppy = 2-phenylpyridine, 2 mol %), and *N*,*N*-diisopropylethylamine (DIPEA, 4 equiv). We faced the problem that available TADF dyes like 4CzIPN were completely inert in this reaction because of their low reduction potentials. In the proposed mechanistic picture,^24^ a reduction of Co(II) to reactive Co(I) was proposed.^39^ In particular, the stronger reductant [Ir(dtbbpy)(ppy)_2_], generated by reductive quenching of the excited state of [Ir(dtbbpy)(ppy)_2_]^**+**^ by DIPEA, is responsible of the reduction of Co(II). We have reinvestigated the cobalt-mediated allylation reaction with the stronger reductants **1** and **2**, considering that the better reducing properties of the two organic dyes were sufficient to trigger the reactivity of the Co(II) center needed for the allylation reaction [namely, the reduction of Co(II) to Co(I)], and allowing us to replace expensive Ir(III) photocatalysts. We were delighted to find out that **2** was active in cobalt-mediated allylation of aldehydes. We set up some key experiments for the evaluation of the key parameters of the reaction (see the Supporting Information for details) using 4-chlorobenzaldehyde (**4a**) as the model substrate (Scheme 2 and Table S1). The reaction proceeds with an excellent yield of homoallylic alcohol in the presence CoBr_2_ (7 mol %), dtbbpy (10 mol %), allyl acetate (**5**, 3 equiv), and Hantzsch’s ester (HE, 2 equiv) as the final reductant in a mixture of THF and H_2_O (9:1) under 456 nm Kessil lamp irradiation, where the photocatalyst absorbs most of the light compared to the other components of the reaction (Figure S2).

**Scheme 3:**
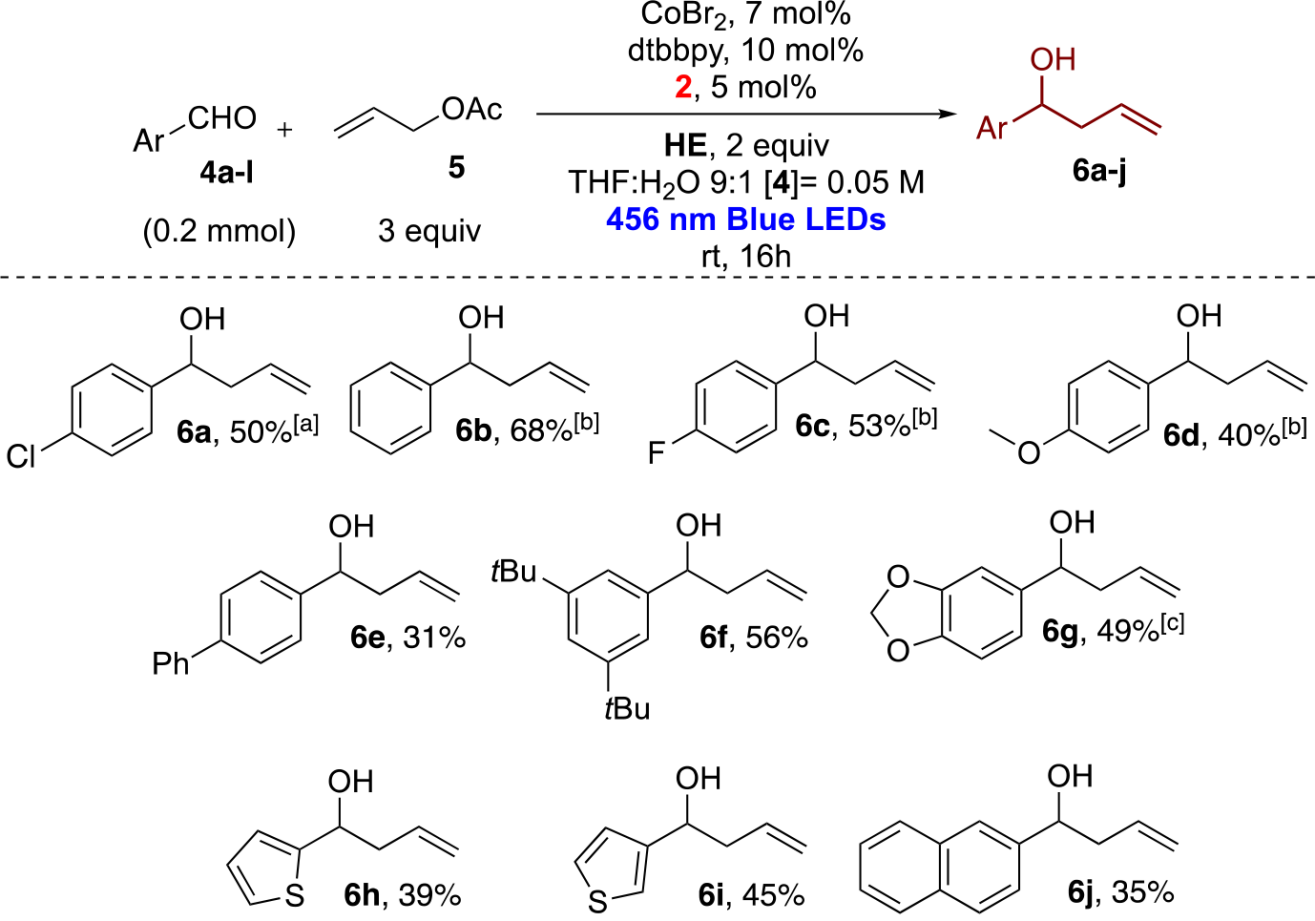
Dual Photoredox Allylation of Aromatic Aldehydes with CoBr_2_ Using **2** as a Photocatalyst (isolated yields reported) Reaction performed on a 1 mmol scale with Co(OAc)_2_ hydrate. Reaction performed on a 0.1 mmol scale. Reaction time of 40 h.

As we have already remarked in the Introduction, the better reducing properties of **2** were the key for the reaction, while 3DPAFIPN or 4CzIPN^29^ were not suitable. The cobalt salt, the photocatalyst, Hantzsch’s ester, and irradiation with visible light are all required for a successful reaction. The absence of the ligand, 4,4′-di-*tert*-butyl-2,2′-dipyridyl (dtbbpy), which was carefully selected in our previous study,^27^ caused the complete consumption of aldehyde **4a** with the formation of the corresponding pinacol product **7a** as the major product, and only 10% of **6a** was detected. We have recently reported that organic photocatalysts can promote pinacol coupling in the presence of HE, which activates the aldehyde and increases its reduction potential for the pinacolization via ketyl radical.^40^ In general, we observed complete conversion in the case of aromatic aldehydes. Byproducts such as pinacol products (10–20%) and benzylic alcohols (10–15%) were observed in the reactions, and this explains the moderate yields of isolated products. During the revision of the manuscript, we determined that other cobalt salts can promote the reaction (see the Supporting Information for details). In particular, Co(OAc)_2_ hydrate was found to be less active than CoBr_2_ but a smaller amount of byproducts was observed in the model reaction. Therefore, the scale-up of the reaction to 1 mmol was performed for 72 h in the presence of this cobalt salt.^41^ The addition of water was found to be important for promoting the allylation with aromatic and aliphatic aldehydes avoiding, in the case of aromatic aldehydes, the favorite pinacol coupling.^27^ The selected conditions were employed for various aromatic aldehydes, as reported in Scheme 3.

In general, as noticed for the reaction performed with the iridium-based photocatalyst, the reactivity was strongly influenced by the aromatic moiety of the aldehydes. Electron rich aldehydes showed a reduced reactivity, and in some cases, we tried to improve the yields by increasing the reaction time, as for **6g**. In other cases, we did not observe better results. The moderate yields can be due to concurrent pinacol coupling or reduction of the aldehydes to the corresponding benzylic alcohol. The better reduction properties of **2** are unfortunately competing with the promotion of the ketyl dimerization. It is important to underline that 3DPAFIPN (**1**) is inert for the reaction and only traces of homoallylic alcohol **6a** were observed. The results obtained are comparable to those obtained with [Ir(III)] photocatalysts, but some aromatic aldehydes were not compatible with the cobalt-mediated process (see the Supporting Information). In particular, the presence of bromine or iodine on the aromatic core is not tolerated, as we observed partial dehalogenation in the isolated product.

**Scheme 4:**
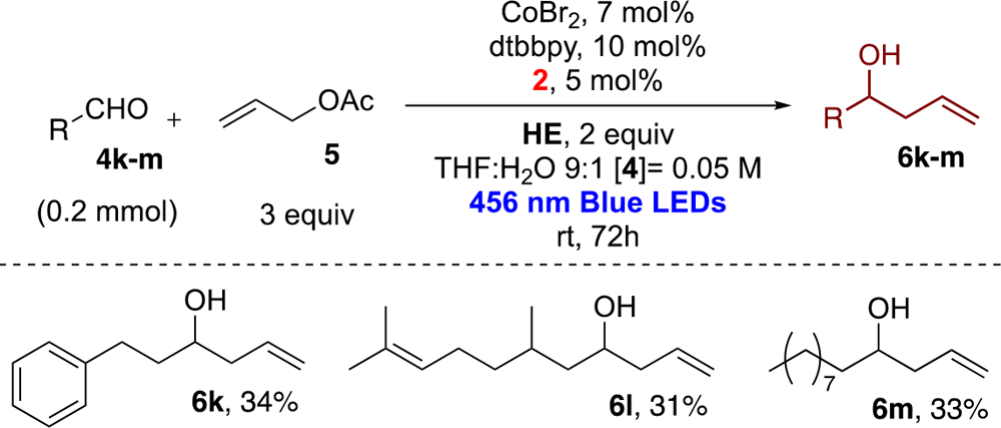
Dual Photoredox Allylation of Aliphatic Aldehydes (isolated yields reported)

Aliphatic aldehydes (Scheme 4), as in the case of Ir(III), suffered from reduced reactivity, as observed also by Shi.^29^ In fact, we have increased the reaction time to 72 h and obtained poor yields with linear aliphatic aldehydes, while branched aldehydes were not reactive (see the Supporting Information).

**Scheme 5:**
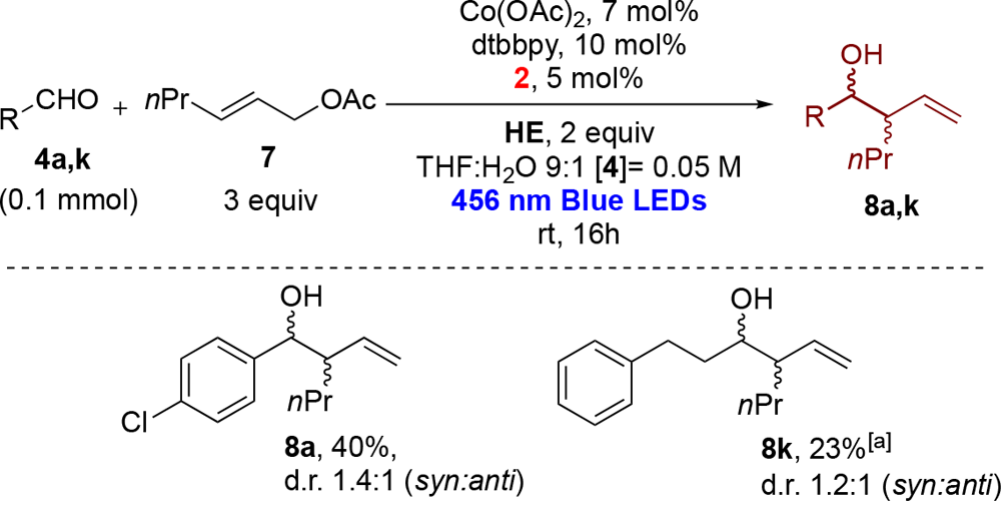
Reaction of Hex-2-en-1-yl Acetate with Aromatic and Aliphatic Aldehydes Reaction time of 48 h.

We have also performed the reaction with a prochiral acetate (Scheme 5), using Co(OAc)_2_ to minimize the tendency to produce byproducts with the less reactive substrates. In general, hex-2-en-1-yl acetate was found to be reactive with aromatic aldehydes, while with aliphatic aldehydes, we observed a scarce reactivity, in line with previous experiments.^27^ In the case of 4-chlorobenzaldehyde, the desired product was obtained in 40% yield with a diastereoisomeric ratio of 1.4:1 (*syn*:*anti*).

**Figure 5:**
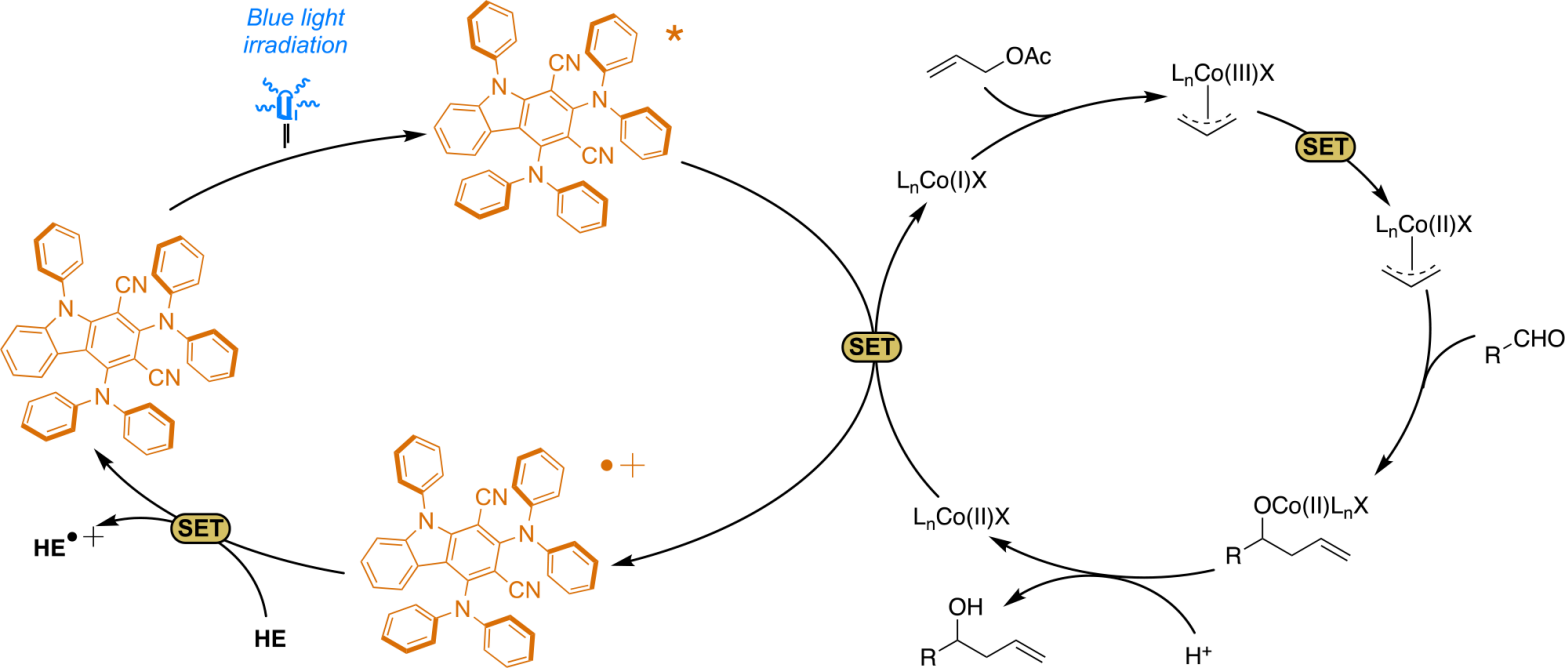
Suggested mechanistic picture for the reaction.

To investigate the photochemical mechanism of the allylation reaction, we performed some luminescence quenching analysis on compound **2** in the presence of all reactants. No change in the TADF emission lifetime was observed upon addition of 4-chlorobenzaldehyde, while allyl acetate and the Co(II) complex in the presence of dtbbpy both showed significant quenching of the delayed fluorescence (Figure S3). However, the estimated quenching constant is much larger for the latter. Unfortunately, we were not able to investigate in detail the quenching of **2** by HE because its absorption spectrum is largely overlapping that of the photocatalyst and, under the experimental conditions used for the luminescence measurements, most of the light is absorbed by HE. However, HE is likely not responsible for the photoreaction because no product formation is observed in the absence of photocatalyst **2** (entry 3, Table S1). On the basis of the experiments performed, we can suggest the mechanistic picture depicted in Figure 5. In recent years, several authors have suggested that the reaction follows a Co(II)–Co(I)–Co(III)–Co(II) cycle.^27,29,39,42^ Co(II) is reduced by **2** in its excited state [E(**2**^•+^/*2) = −1.31 vs SCE] to Co(I) {*E*[Co(II)/Co(I)] = −1.05 V vs SCE^43^} that is reacting with allyl acetate to form a Co(III) allyl intermediate. The Co(III) allyl is then further reduced by a SET event (**2** or HE^•+^) to Co(II) allyl,^39^ the reactive organometallic species that can react with aldehydes by a Zimmerman–Traxler transition state.^41^**2** [*E*(**2**^•+^/**2**) = +1.31 V vs SCE (see Table 1)] was restored to the initial state by HE [E(HE^•+^/HE) = +1.0 V vs SCE].^44^

## Conclusions

The photodegradation of TADF-active halo-isophthalonitriles must be considered as a key factor for their use as photocatalysts and for the design of suitable photoredox-promoted chemical transformations. In this paper, we demonstrated that the photoconversion of 3DPAFIPN affords an easily isolated carbazole-1,3-dicarbonitrile derivative (**2**) that is also showing peculiar photophysical properties, including TADF. Specifically, the lifetime of its delayed fluorescence and the modulation of the redox potentials in the ground and excited states are useful properties to employ in photoredox-activated catalysis in the presence of a Co(II) species. Dual metal and photoredox catalysis has been employed for the efficient allylation of aldehydes to afford homoallylic alcohols in good yields. The employment of the new dyes **2** in different photoredox catalytic reactions and the development of a stereocontrolled photoredox version of this reaction are under investigation in our laboratory.^45^

## Experimental Section

### General Methods and Materials

^1^H NMR and ^13^C NMR spectra were recorded on a Varian Mercury 400 spectrometer. Chemical shifts are reported in parts per million from TMS with the solvent resonance as the internal standard (CHCl_3_, δ 7.26; CDCl_3_, δ 77.0). Data are reported as follows: chemical shift, multiplicity (s, singlet; d, duplet; t, triplet; q, quartet; dd, double duplet; m, multiplet), coupling constants (hertz). Structural assignments were made with additional information from gCOSY, gHSQC, and gHMBC experiments. Chromatographic purification was performed with 240–400 mesh silica gel. HPLC-MS analyses were performed on an Agilent Technologies HP1100 instrument coupled with an Agilent Technologies MSD1100 single-quadrupole mass spectrometer using a Phenomenex Gemini C18 3 μm (100 mm × 3 mm) column; mass spectrometric detection was performed in full-scan mode from *m*/*z* 50 to 2500, with a scan time of 0.1 s in positive ion mode, an ESI spray voltage of 4500 V, nitrogen gas at 35 psi, a drying gas flow rate of 11.5 mL min^–1^, and a fragmentor voltage of 30 V. HRMS was performed on a Waters Xevo G2-XS QTof instrument, ESI^+^, with a cone voltage of 40 V, a capillary voltage of 3 kV, and a source temperature of 120 °C. All reactions were set up under an argon atmosphere in oven-dried glassware using standard Schlenk techniques. The reaction mixture was irradiated with a Kessil PR160L@456 nm instrument (see Figure S20 for the emission profile). Diethyl 2,6-dimethyl-1,4-dihydropyridine-3,5-dicarboxylate (Hantzsch’s ester)^46^ was prepared following a literature procedure.

### Procedure for the Synthesis of 3DPAFIPN (**1**)

We adapted the procedure reported by Zeitler and co-workers.^7^ A 50 mL round-bottom flask, equipped with a magnetic stirring bar, was charged with diphenylamine (5.0 equiv, 10 mmol, 1.69 g) and dry THF (20 mL). The solution was cooled to 0 °C, and NaH (60% in mineral oil, 7.5 equiv, 15 mmol, 600 mg) was slowly added under vigorous stirring. After 2 h, tetrafluoroisophthalonitrile (1.0 equiv, 2 mmol, 400 mg) was added, and the mixture was stirred at room temperature in the dark. The solution slowly turned from colorless to bright yellow. When the TLC showed a complete consumption of the starting material (usually 2 days is needed), water (1 mL) was added dropwise under vigorous stirring to decompose the excess of NaH, and the mixture was evaporated to give a yellow solid. The solid was washed with water (10 mL) and ethyl acetate (20 mL), and the suspension was filtered over a Gooch crucible. The bright yellow solid was dried under vacuum to afford NMR-pure 3DPAFIPN (1.04 g, 1.6 mmol, 80% yield). By HPLC/MS analysis, the formation of 5–7 mol % **3** that was not detected by ^1^H NMR was observed after purification. To obtain pure 3DPAFIPN **1**, the product was further purified by flash chromatography (SiO_2_, 98:2 cyHex/Et_2_O) to obtain pure 3DPAFIPN **1** as a bright yellow solid: ^1^H NMR (400 MHz, CDCl_3_) δ 7.25 (m, 12H), 7.11–7.01 (m, 6H), 7.01–6.86 (m, 12H); ^13^C{^1^H} NMR (100 MHz, CDCl_3_) δ 152.4 (d, *J* = 259.5 Hz, 1C), 151.8 (d, *J* = 4.0 Hz, 1C), 145.5 (2C), 145.3 (4C), 143.0 (d, *J* = 11.0 Hz, 2C), 129.43 (8C), 129.36 (4C), 124.6 (4C), 124.0 (2C), 122.73 (8C), 122.70 (4C), 112.6 (d, *J* = 3.4 Hz, 2C) 108.9 (d, *J* = 3.2 Hz, 2C).

### Procedure for the Synthesis of 2DPAPhCzDCN (**2**)

A dry 20 mL Schlenk tube, equipped with a Rotaflo stopcock and a magnetic stirring bar under an argon atmosphere, was first charged with 3DPAFIPN (0.09 mmol, 60 mg). Then, inhibitor-free dry THF (10 mL) was added, and the reaction mixture was irradiated with a blue Kessil lamp (456 nm) ∼15 cm from the light source, under vigorous stirring for 48 h. After that, the solvent was removed under reduced pressure. The crude solid was purified by flash column chromatography (SiO_2_, DCM) to afford product **2** as a bright yellow solid in 78% (44 mg, 0.07 mmol): ^1^H NMR (400 MHz, CDCl_3_) δ 7.69 (d, *J* = 8.0 Hz, 1H), 7.57 (m, 3H), 7.49 (m, 2H), 7.36 (t, *J* = 7.5 Hz, 1H), 7.30–7.15 (m, 16H overlapped with the residual peak of the solvent), 7.12–7.04 (m, 3H), 7.04–6.96 (m, 8H); ^13^C{^1^H} NMR (100 MHz, CDCl_3_) δ 153.2, 148.2, 146.0 (2C), 145.1 (2C), 144.5, 143.2, 134.9, 130.4, 129.8, 129.4 (4C), 129.43, 129.37, 129.2, 129.2 (4C), 129.1, 127.7, 124.6, 123.5 (2C), 123.5 (2C), 122.9, 122.74 (4C), 122.4, 121.8 (4C), 121.7, 121.0, 120.9, 119.6, 114.2, 112.2, 110.5, 108.0; HRMS (ESI/Q-TOF) *m*/*z* [M + H]^+^ calcd for C_44_H_30_N_5_ 628.2496, found 628.2495; HRMS (ESI/Q-TOF) *m*/*z* [M + K]^+^ calcd for C_44_H_29_KN_5_ 666.2055, found 666.2054.

### Standard Procedure for Photoredox Cobalt-Catalyzed Allylation of Aldehydes

All of the reactions were performed on a 0.2 mmol scale of aldehyde, or in duplicate on a 0.1 mmol scale. A dry 10 mL Schlenk tube, equipped with a Rotaflo stopcock, a magnetic stirring bar, and an argon supply tube, was first charged with CoBr_2_·6H_2_O (7 mol %, 14 μmol, 4.6 mg) that was flame-dried under vacuum to remove the presence of water. Then 4,4′-di-*tert*-butyl-2,2′-dipyridyl (dtbbpy) (10 mol %, 20 μmol, 5.4 mg) and freshly distilled inhibitor-free THF (1 mL) were added. The reaction was kept under vigorous stirring for a few minutes, and then substrate **4** (0.2 mmol), organic photocatalyst **2** (5 mol %, 0.01 mmol, 6.3 mg), and diethyl 1,4-dihydro-2,6-dimethyl-3,5-pyridinedicarboxylate HE (2 equiv, 0.4 mmol, 101 mg) were added. THF (2.6 mL) and distilled water (0.4 mL) were then added; the reaction mixture was further subjected to a freeze–pump–thaw procedure (three cycles), and the vessel was refilled with argon. Then, allyl acetate **5** (3 equiv, 0.6 mmol, 60 mg, 65 μL) was added. The reaction mixture was irradiated with a blue Kessil lamp (456 nm) ∼15 cm from the light source, under vigorous stirring from 16 to 72h. After that, the reaction was quenched with water (approximately 4 mL) and the mixture extracted with EtOAc (3 × 10 mL). The combined organic layers were dried over anhydrous Na_2_SO_4_, and the solvent was removed under reduced pressure. The crude was purified by flash column chromatography (100% DCM) to afford products **6** in the stated yields.

### 1-(4-Chlorophenyl) But-3-en-1-ol (**6a**)

Pale yellow oil, 87% (16 mg, 0.088 mmol). The general procedure (16 h) was applied using **4a** (0.1 mmol, 14 mg) and **5** (0.3 mmol, 3 equiv, 32 μL). The title compound was isolated by flash column chromatography (100% DCM). Spectroscopic data were according to the literature:^47^^1^H NMR (400 MHz, CDCl_3_, 25 °C) δ 7.31–7.26 (m, 4H), 5.81–5.71 (m, 1H), 5.17–5.11 (m, 2H), 4.71 (dd, *J* = 7.8, 5.1 Hz, 1H), 2.52–2.39 (m, 2H); ^13^C{^1^H} NMR (100 MHz, CDCl_3_, 25 °C) δ 142.2, 133.9, 133.1, 128.5 (2C), 127.2 (2C), 118.9, 72.5, 43.9.

### 1-Phenylbut-3-en-1-ol (**6b**)

Pale yellow oil, 68% (10 mg, 0.068 mmol). The general procedure (16 h) was applied using previously distilled **4b** (0.1 mmol, 10 μL) and **5** (0.3 mmol, 3 equiv, 32 μL). The title compound was isolated by flash column chromatography (100% DCM). Spectroscopic data were according to the literature:^47^^1^H NMR (400 MHz, CDCl_3_, 25 °C) δ 7.35–7.24 (m, 5H), 5.81–5.76 (m, 1H), 5.18–5.11 (m, 2H), 4.72 (dd, *J* = 7.6, 5.4 Hz, 1H), 2.52–2.49 (m, 2H), 2.10 (br s, 1H); ^13^C{^1^H} NMR (100 MHz, CDCl_3_, 25 °C) δ 143.9, 134.5, 128.4 (2C), 127.5, 125.8 (2C), 118.3, 73.3, 43.8.

### 1-(4-Fluorophenyl) But-3-en-1-ol (**6c**)

Pale yellow oil, 53% (9 mg, 0.054 mmol). The general procedure (16 h) was applied using previously distilled **4c** (0.1 mmol, 11 μL) and **5** (0.3 mmol, 3 equiv, 32 μL). The title compound was isolated by flash column chromatography (100% DCM). Spectroscopic data were according to the literature:^48^^1^H NMR (400 MHz, CDCl_3_, 25 °C) δ 7.33–7.29 (m, 2H), 7.05–6.99 (m, 2H), 5.82–5.72 (m, 1H), 5.17–5.12 (m, 2H), 4.71 (dd, *J* = 7.9, 4.8 Hz, 1H), 2.52–2.43 (m, 2H), 1.88 (br s, 1H); ^13^C{^1^H} NMR (100 MHz, CDCl_3_, 25 °C) δ 163.3, 160.9 139.5, 134.1, 127.4 (2C), 118.7, 115.3, 115.1, 72.6, 43.9; ^19^F NMR (377 MHz, CDCl_3_, 25 °C) δ −115.23 (m, 1F).

### 1-(4-Methoxyphenyl) But-3-en-1-ol (**6d**)

Pale yellow oil, 40% (7.2 mg, 0.040 mmol). The general procedure (16 h) was applied using previously distilled **4d** (0.1 mmol, 12 μL) and **5** (0.3 mmol, 3 equiv, 32 μL). The title compound was isolated by flash column chromatography (100% DCM). Spectroscopic data were according to the literature:^47^^1^H NMR (400 MHz, CDCl_3_, 25 °C) δ 7.29–7.24 (m, 2H), 6.89–6.85 (m, 2H), 5.79 (ddt, *J* = 17.2, 10.2, 7.1 Hz, 1H), 5.17–5.09 (m, 2H), 4.67 (t, *J* = 6.5 Hz, 1H), 3.79 (s, 3H), 2.51–2.47 (m, 2H); ^13^C{^1^H} NMR (100 MHz, CDCl_3_, 25 °C) δ 159.0, 136.0, 134.6, 127.0 (2C), 118.2, 113.8 (2C), 72.9, 55.3, 43.7.

### 1-([1,1′-Biphenyl]-4-yl) But-3-en-1-ol (**6e**)

Pale yellow oil, 31% (14 mg, 0.063 mmol). The general procedure (16 h) was applied using **4e** (0.2 mmol, 36 mg) and **5** (0.6 mmol, 3 equiv, 65 μL). The title compound was isolated by flash column chromatography (100% DCM). Spectroscopic data were according to the literature:^47^^1^H NMR (400 MHz, CDCl_3_, 25 °C) δ 7.59–7.57 (m, 4H), 7.43–7.41 (m, 4H), 7.35–7.31 (m, 1H), 5.89–5.79 (m, 1H), 5.22–5.14 (m, 2H), 4.78 (t, *J* = 5.4 Hz, 1H), 2.58–2.52 (m, 2H), 2.07 (br s, 1H); ^13^C{^1^H} NMR (100 MHz, CDCl_3_, 25 °C) δ 142.9, 140.8, 140.5, 134.4, 128.7 (2C), 127.2, 127.1 (2C), 127.0 (2C), 126.2 (2C), 118.5, 73.0, 43.8.

### 1-(3,5-Di-*tert*-butylphenyl) But-3-en-1-ol (**6f**)

Pale yellow oil, 56% (29 mg, 0.11 mmol). The general procedure (16 h) was applied using **4f** (0.2 mmol, 44 μL) and **5** (0.6 mmol, 3 equiv, 65 μL). The title compound was isolated by flash column chromatography (100% DCM). Spectroscopic data were according to the literature:^49^^1^H NMR (400 MHz, CDCl_3_, 25 °C) δ 7.35 (s, 1H), 7.20 (s, 2H), 5.91–5.81 (m, 1H), 5.21–5.13 (m, 2H), 4.71 (t, *J* = 5.4 Hz, 1H), 1.94 (br s, 1H), 1.33 (s, 18H); ^13^C{^1^H} NMR (100 MHz, CDCl_3_, 25 °C) δ 150.8, 143.1, 135.0, 121.6, 120.0, 118.0, 74.1, 43.9, 34.9, 31.5.

### 1-(Benzo[*d*][1,3]dioxol-5-yl) But-3-en-1-ol (**6g**)

Pale yellow oil, 49% (19 mg, 0.096 mmol). The general procedure (16 h) was applied using **4g** (0.2 mmol, 30 mg) and **5** (0.6 mmol, 3 equiv, 65 μL). The title compound was isolated by flash column chromatography (100% DCM). Spectroscopic data were according to the literature:^47^^1^H NMR (400 MHz, CDCl_3_, 25 °C) δ 6.86 (d, *J* = 1.5 Hz, 1H), 6.80–6.74 (m, 2H), 5.93 (s, 2H), 5.82–5.72 (m, 1H), 5.16–5.10 (m, 2H), 4.62 (t, *J* = 6.5 Hz, 1H), 2.47–2.43 (m, 2H), 2.01 (br s, 1H); ^13^C{^1^H} NMR (100 MHz, CDCl_3_, 25 °C) δ 147.7, 146.9, 137.9, 134.4, 119.2, 118.4, 108.0, 106.4, 101.0, 73.2, 43.8.

### 1-(Thiophen-2-yl) But-3-en-1-ol (**6h**)

Pale yellow oil, 39% (12 mg, 0.078 mmol). The general procedure (16 h) was applied using previously distilled **4h** (0.2 mmol, 19 μL) and **5** (0.6 mmol, 3 equiv, 65 μL). The title compound was isolated by flash column chromatography (100% DCM). Spectroscopic data were according to the literature:^48^^1^H NMR (400 MHz, CDCl_3_) δ 7.25–7.21 (m, 1H), 7.00–6.92 (m, 2H), 5.82 (ddt, *J* = 17.2, 10.2, 7.1 Hz, 1H), 5.23–5.11 (m, 2H), 4.98 (t, *J* = 6.4 Hz, 1H), 2.69–2.53 (m, 2H), 2.19 (s, 1H); ^13^C{^1^H} NMR (100 MHz, CDCl_3_) δ 147.76, 133.79, 126.60, 124.54, 123.66, 118.80, 69.34, 43.76.

### 1-(Thiophen-3-yl) But-3-en-1-ol (**6i**)

Pale yellow oil, 45% (14 mg, 0.09 mmol). The general procedure (16 h) was applied using previously distilled **4i** (0.2 mmol, 19 μL) and **5** (0.6 mmol, 3 equiv, 65 μL). The title compound was isolated by flash column chromatography (100% DCM). Spectroscopic data were according to the literature:^48^^1^H NMR (400 MHz, CDCl_3_, 25 °C) δ 7.29 (dd, *J* = 5.0, 3.0 Hz, 1H), 7.19 (d, *J* = 2.9 Hz, 1H), 7.07 (dd, *J* = 5.0, 1.2 Hz, 1H), 5.84–5.74 (m, 1H), 5.18–5.11 (m, 2H), 4.83 (dt, *J* = 8.0, 4.2 Hz, 1H), 2.58–2.45 (m, 2H), 2.09 (br s, 1H); ^13^C{^1^H} NMR (100 MHz, CDCl_3_, 25 °C) δ 145.3, 134.2, 126.0, 125.6, 120.7, 118.5, 69.5, 43.0.

### 1-(Naphthalen-2-yl) But-3-en-1-ol (**6j**)

Pale yellow oil, 35% (14 mg, 0.070 mmol). The general procedure (16 h) was applied using **4j** (0.2 mmol, 31 mg) and **5** (0.6 mmol, 3 equiv, 65 μL). The title compound was isolated by flash column chromatography (100% DCM). Spectroscopic data were according to the literature:^47^^1^H NMR (400 MHz, CDCl_3_, 25 °C) δ 7.84–7.79 (m, 4H), 7.49–7.45 (m, 3H), 5.88–5.77 (m, 1H), 5.21–5.12 (m, 2H), 4.89 (dd, *J* = 7.2, 5.9 Hz, 1H), 2.66–2.53 (m, 2H), 2.12 (br s, 1H); ^13^C{^1^H} NMR (100 MHz, CDCl_3_, 25 °C) δ 141.2, 134.3, 133.2, 132.9, 128.2, 127.9, 127.6, 126.1, 125.8, 124.5, 124.0, 118.5, 73.5, 43.7.

### 1-Phenylhex-5-en-3-ol (**6k**)

Pale yellow oil, 34% (12 mg, 0.068 mmol). The general procedure (72 h) was applied using previously distilled **4k** (0.2 mmol, 26 μL) and **5** (0.6 mmol, 3 equiv, 65 μL). The title compound was isolated by flash column chromatography (100% DCM). Spectroscopic data were according to the literature:^47^^1^H NMR (400 MHz, CDCl_3_, 25 °C) δ 7.29–7.17 (m, 5H), 5.81–5.79 (m, 1H), 5.16–5.11 (m, 2H), 3.66 (ddd, *J* = 12.2, 7.6, 4.7 Hz, 1H), 2.80–2.75 (m, 1H), 2.72–2.64 (m, 1H), 2.31–2.28 (m, 1H), 2.24–2.15 (m, 1H), 1.81–1.75 (m, 2H), 1.61 (br s, 1H); ^13^C{^1^H} NMR (100 MHz, CDCl_3_, 25 °C) δ 142.0, 134.6, 128.4 (2C), 128.4 (2C), 125.8, 118.3, 69.9, 42.0, 38.4, 32.0.

### 6,10-Dimethylundeca-1,9-dien-4-ol (**6l**)

Pale yellow oil, 31% (12 mg, 0.061 mmol). The general procedure (72 h) was applied using previously distilled **4l** (0.2 mmol, 36 μL) and **5** (0.6 mmol, 3 equiv, 65 μL). The title compound was isolated by flash column chromatography (100% DCM) as a mixture of *syn* and *anti* diasteroisomers. Spectroscopic data were according to the literature:^47^^1^H NMR (400 MHz, CDCl_3_, 25 °C) mixture of diastereoisomers δ 5.86–5.76 (m, 1H), 5.14–5.08 (m, 3H), 3.76–3.70 (m, 1H), 2.28–2.22 (m, 1H), 2.16–2.04 (m, 1H), 2.04–1.87 (m, 2H), 1.66 (s, 3H), 1.69–1.55 (m, 3H), 1.58 (s, 3H), 1.51–1.05 (m, 2H), 0.90 (dd, *J* = 6.4, 6.4 Hz, 3H); ^13^C{^1^H} NMR (100 MHz, CDCl_3_, 25 °C) mixture of diastereoisomers δ 135.1, 135.0, 131.4, 124.9, 118.3, 118.2, 68.9, 68.5, 44.5, 44.4, 42.9, 42.3, 38.0, 36.9, 29.5, 29.1, 25.9, 25.6, 25.6, 20.4, 19.3, 17.8.

### Tridec-1-en-4-ol (**6m**)

Pale yellow oil, 33% (13 mg, 0.066 mmol). The general procedure (72 h) was applied using previously distilled **4m** (0.2 mmol, 38 μL) and **5** (0.6 mmol, 3 equiv, 65 μL). The title compound was isolated by flash column chromatography (100% DCM). Spectroscopic data were according to the literature:^48^^1^H NMR (400 MHz, CDCl_3_) δ 5.83 (dddd, *J* = 20.4, 9.6, 7.9, 6.5 Hz, 1H), 5.17–5.10 (m, 2H), 3.64 (dtd, *J* = 7.7, 5.9, 4.1 Hz, 1H), 2.34–2.26 (m, 1H), 2.13 (dt, *J* = 13.7, 7.9 Hz, 1H), 1.60 (s, 2H), 1.44 (d, *J* = 2.7 Hz, 1H), 1.26 (t, *J* = 3.2 Hz, 12H), 0.90–0.85 (m, 4H); ^13^C{^1^H} NMR (100 MHz, CDCl_3_) δ 134.9, 118.0, 70.7, 41.9, 36.8, 31.9, 29.6, 29.6, 29.5, 29.3, 25.6, 22.6, 14.1.

### 1-(4-Chlorophenyl)-2-vinylpentan-1-ol (**8a**)

Isolated as mixture of diasteroisomers, 1.4:1 *syn*:*anti*, colorless oil, 40% (9 mg, 0.040 mmol). The general procedure (16 h) was applied using **4a** (0.1 mmol, 14 mg) and **7** (0.3 mmol, 3 equiv, 43 mg). The title compound was isolated by flash column chromatography (7:3 DCM/hexane, then 8:1 hexane/ethyl acetate). Spectroscopic data were according to the literature:^50^^1^H NMR (400 MHz, CDCl_3_) δ 7.31–7.25 (m, 6H), 7.20–7.17 (m, 2H), 5.61 (ddd, *J* = 17.2, 10.3, 9.2 Hz, 1H, *anti* form), 5.45 (ddd, *J* = 17.1, 10.3, 9.1 Hz, 1H, *syn* form), 5.27–5.13 (m, 2H, *anti* form), 5.09–4.96 (m, 2H, *syn* form), 4.59 (m, 1H, *syn* form), 4.35 (d, *J* = 8.2 Hz, 1H, *anti* form), 2.37 (m, 1H), 2.27–2.19 (m, 1H), 2.18 (d, *J* = 2.3 Hz, 1H), 2.00 (d, *J* = 4.6 Hz, 1H), 1.51–1.36 (m, 2H), 1.35–1.27 (m, 2H), 1.14 (m, 6H), 0.84 (t, *J* = 7.1 Hz, 3H), 0.77 (t, *J* = 7.0 Hz, 3H); ^13^C{^1^H} NMR (100 MHz, CDCl_3_) δ 141.0, 140.9, 138.9, 138.1, 133.2, 133.0, 128.3, 128.3, 128.1, 128.0, 119.1, 117.7, 76.2, 76.0, 52.6, 51.2, 32.5, 31.7, 20.3, 20.3, 14.0, 13.8; ^13^C{^1^H} (100 MHz, CDCl_3_) δ 141.0 (*anti* form), 140.9 (*syn* form), 138.9 (*anti* form), 138.1 (*syn* form), 133.2 (*anti* form), 133.0 (*syn* form), 128.3 (*syn*+*anti* form), 128.1 (*anti* form), 128.0 (*syn* form), 119.1 (*anti* form), 117.7 (*syn* form), 76.2 (*syn* form), 75.9 (*anti* form), 52.6 (*anti* form), 51.2 (*syn* form), 32.5 (*anti* form), 31.7 (*syn* form), 20.3 (*syn*+*anti* form), 14.0 (*syn* form), 13.8 (*anti* form).

### 1-{henyl-4-vinylheptan-3-ol (**8k**)

Isolated as mixture of diasteroisomers, 1.2:1 *syn*:*anti*, colorless oil, 23% (5 mg, 0.023 mmol). The general procedure (48 h) was applied using previously distilled **4k** (0.1 mmol, 13 μL) and **7** (0.3 mmol, 3 equiv, 43 mg). The title compound was isolated by flash column chromatography (7:3 DCM/hexane, then 8:1 hexane/ethyl acetate). Spectroscopic data were according to the literature:^51^^1^H NMR (400 MHz, CDCl_3,_*syn+anti*) δ 7.28 (d, *J* = 7.7 Hz, 2H), 7.22–7.12 (m, 3H), 5.63–5.51 (m, 1H), 5.19–5.03 (m, 2H), 3.54–3.41 (m, 1H), 2.87–2.77 (m, 1H), 2.70–2.58 (m, 1H), 2.14–2.01 (m, 1H), 1.89–1.77 (m, 1H), 1.76–1.57 (m, 1H), 1.43–1.28 (m, 4H), 0.85 (t, *J* = 8.0 Hz, 3H); ^13^C{^1^H} NMR (100 MHz, CDCl3) δ 142.3 (*syn+anti* form), 138.9 (*anti* form), 138.8 (*syn* form), 128.4 (*syn* form, 2H), 128.3 (*anti* form, 2H), 125.7 (*syn+anti* form), 118.0 (*syn* form), 117.4 (*anti* form), 73.7 (*anti* form), 72.9 (*syn* form), 50.6 (*anti* form), 50.3 (*syn* form), 36.5 (*syn* form), 35.7 (*anti* form), 32.9 (*syn* form), 32.4 (*anti* form), 32.3 (*anti* form), 32.1 (*syn* form), 20.4 (*syn+anti* form), 14.0 (*syn+anti* form).

### Procedure for Photoredox Cobalt-Catalyzed Allylation of **4a** on a 1 mmol Scale

The general procedure (72 h) was applied using **4a** (1 mmol, 140 mg), Co(OAc)_2_·4H_2_O (7 mol %, 70 μmol, 18 mg), 4,4′-di-*tert*-butyl-2,2′-dipyridyl (dtbbpy) (10 mol %, 0.10 mmol, 27 mg), **2** (5 mol %, 50 μmol, 31 mg), diethyl 1,4-dihydro-2,6-dimethyl-3,5-pyridinedicarboxylate HE (2 equiv, 2 mmol, 506 mg), **5** (3 mmol, 3 equiv, 330 μL), freshly distilled inhibitor-free THF (18 mL), and distilled water (2 mL). The title compound was isolated by flash column chromatography (7:3 DCM/hexane) as a pale yellow oil (50%, 90 mg, 0.49 mmol). Spectroscopic data were according to the literature.^47^

### Photophysical, Electrochemical, and Mechanistic Studies

All of the photophysical analyses were carried out in air-equilibrated tetrahydrofuran at 298 K unless otherwise specified. UV–vis absorption spectra were recorded with a PerkinElmer λ40 spectrophotometer using quartz cells with an optical path length of 1.0 cm. Degassed solutions were obtained by means of repeated pump–freeze–thaw cycles (∼4 × 10^–6^ mbar) in sealed quartz cuvettes. Luminescence spectra were recorded with a PerkinElmer LS-50, a Varian Cary Eclipse, or an Edinburgh FLS920 spectrofluorimeter equipped with a Hamamatsu R928 phototube. The estimated experimental errors are 2 nm on the band maximum and 5% on the molar absorption coefficient and luminescence lifetime.

Luminescence measurements at 77 K were performed in a DCM/MeOH [1:1 (v/v)] mixture using quartz tubes. Fluorescence lifetimes were measured with an Edinburgh FLS920 spectrofluorometer by a time-correlated single-photon counting (TCSPC) technique. Thermally activated delayed fluorescence (TADF) lifetimes were measured with a PerkinElmer LS55 spectrofluorometer. Emission quantum yields were measured using perylene in MeOH (Φ_FLUO_ = 92%) as the standard.^52^ TADF quantum yields were calculated by knowing Φ_FLUO_ and the intensity ratio between prompt and delayed fluorescence. Singlet oxygen quantum yields were measured with an Edinburgh FLS920 spectrofluorometer equipped with a Ge detector using tetraphenyl porphyrin (TPP) in THF (Φ_Δ_ = 62%) as the standard.^53^ Cyclic voltammetry was performed at room temperature by using an EcoChemie Autolab 30 potentiostat in a three-electrode setup [glassy carbon working electrode (*d* = 3 mm), silver wire quasi-reference electrode, and Pt wire counter electrode] in anhydrous MeCN (supporting electrolyte, 0.05 M TEAPF_6_) and using Fc^+^/Fc as the internal standard (Fc^+^/Fc = +0.38 V vs SCE). The working electrode was polished with 0.03 μm alumina paste, rinsed with water and acetone, and finally blow-dried.

## Supporting Information Available

Results of the photophysical study, screening of reaction conditions for the photoredox reaction, copies of NMR spectra, and analyses of the determination of the structure of compound **2** (PDF)
