# Supplementary material for: Light-Induced Access to Carbazole-1,3-dicarbonitrile: A Thermally Activated Delayed Fluorescent (TADF) Photocatalyst for Cobalt-Mediated Allylations
Source: J Org Chem. 2022 Nov 16;88(10):6390–400. doi: 10.1021/acs.joc.2c01825 (PMC10204086; doi:10.1021/acs.joc.2c01825)

SUPPORTING INFORMATION

LIGHT INDUCED ACCESS TO CARBAZOLE-1,3-DICARBONITRILE: A THERMALLY ACTIVATED DELAYED FLUORESCENT (TADF) PHOTOCATALYST FOR COBALT MEDIATED ALLYLATIONS

Emanuele Pinosa,[a,b] Elena Bassan,[a,b] Sultan Cetin,[a] Marco Villa,[a,b] Simone Potenti,[a,c] Francesco Calogero,[a,b] Andrea Gualandi,*[a,b] Andrea Fermi,*[a,b] Paola Ceroni,[a,b] and Pier Giorgio Cozzi*[a,b]

[a] ALMA MATER STUDIORUM - Università di Bologna, Dipartimento di Chimica “G. Ciamician”, Via Selmi 2, 40126, Bologna, Italy

[b] Center for Chemical Catalysis - C3, Alma Mater Studiorum - Università di Bologna, Via Selmi 2, 40126 Bologna, Italy

[c] Scuola Normale Superiore, Piazza dei Cavalieri 7, 56126 Pisa, Italy

corresponding authors e-mails: andrea.gualandi10@unibo.it; andrea.fermi2@unibo.it, piergiorgio.cozzi@unibo.it- Università di Bologna, via Selmi 2, 40126 Bologna, Italy

Table of contents

Photophysical Studies	S3
Photophysical measurements at 77 K	S3
NMR studies of compounds **1** and **2**	S6
Peaks assignments for **1**	S8
Peaks assignments for **2**	S12
2D NMR spectra	S16
Photoredox reactions	S22
Reaction set-up	S23
Copies of NMR spectra for homoallylic alcohols	S25

Photophysical Studies

Photophysical measurements at 77 K

Figure S1 Emission spectra of derivative **1** (a, λ_{ex} = 420nm) and **2** (b, λ_{ex} = 405 nm) in a glassy matrix (DCM:MeOH 1:1, v/v) at 77K. Emission spectra were registered in fluorescence mode (solid line) and phosphorescence mode with gate time of 50 ms and delay time of 0.05 ms (dashed line) or 0 ms (dotted line).

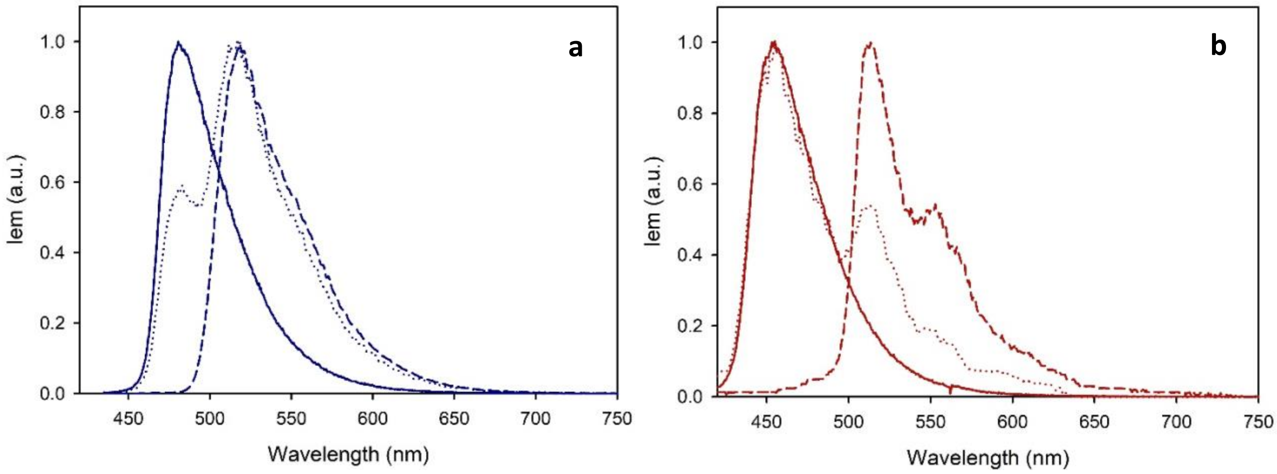

Figure S1 shows a comparison of the fluorescence (solid lines) and phosphorescence (dashed lines) of compounds **1** and **2** in rigid matrix at 77 K. The emission spectra registered with gate time of 50 ms and without delay time (dotted lines) correspond to a convolution of the fluorescence and phosphorescence bands.

Figure S2 (a) Absorption spectra of **2** (2.5×10^{-3} M, black line), Hantzsch ester (**HE**, 0.5 M, red line), $CoBr_2$ and dtbbpy (each of them at concentration of 5×10^{-3} M, green line), 4-chlorobenzaldehyde (0.05 M, blue line), allyl acetate (0.15 M, cyan line) in THF/H_2O (9:1 v/v) and Kessil lamp profile (λ_{em} = 456 nm, purple line). The concentrations are the same used in the reaction conditions. (b) Absorption spectra of the reaction components in the region of the emission of the Kessil Lamp (420nm - 500nm).

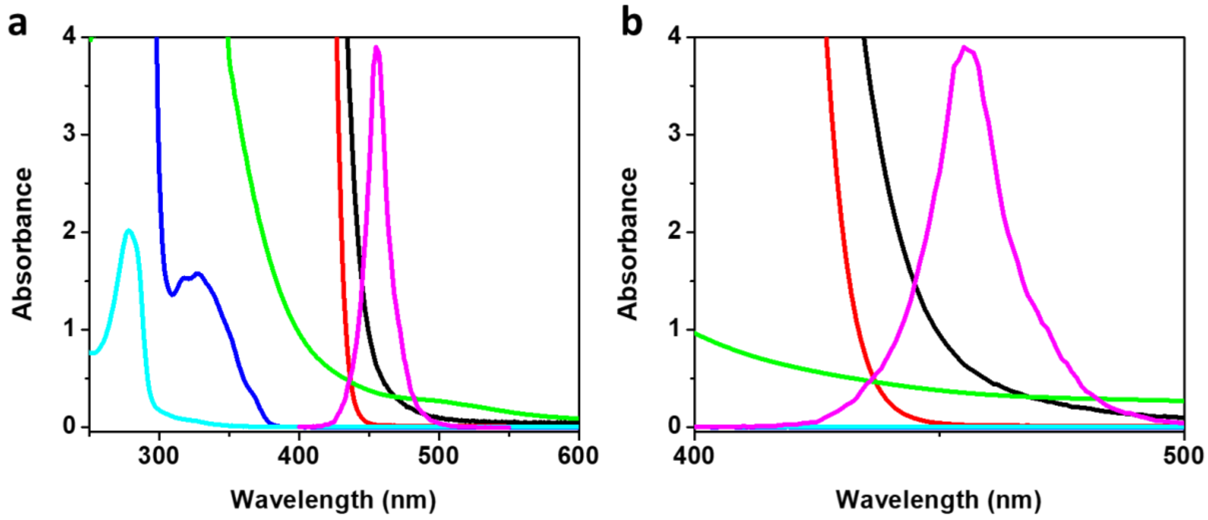

Figure S2 demonstrates that the photocatalyst **2** is not the only species absorbing the 456 nm Kessil light in the reaction of allylation of 4-chlorobenzaldehyde. Based on the absorption spectra of these species and the profile of the irradiation source it is estimated that 65% of light is absorbed by **2,** 10% by **HE** and 25% by the Co(II) complex, while 4-chlorobenzaldehyde and allyl acetate do not absorb light in this range of wavelengths. We decided to use 456 nm to mitigate the absorption of both HE and Co(II) complex and maximize the light absorbed by **2**. At lower wavelength **HE** is absorbing more light compared to photocatalyst **2**.

Investigations on emission quenching kinetics

Figure S3 Absorption spectra (a,b,c), time resolved decay of prompt (d, e, f) and delayed (g, h, i) fluorescence of **2** (ca. 10^{-5} M, in THF/H_2O 9:1, v/v) and upon addition of 4-chlorobenzaldehyde 5 × 10^{-2} M (a, d, g), allyl acetate 1.5 × 10^{-1} M (b, e, h), solution of $CoBr_2$ and dtbbpy (c, f, i). λ_{exc} = 405 nm (d, e, f); λ_{exc} = 420 nm (g, h, i).

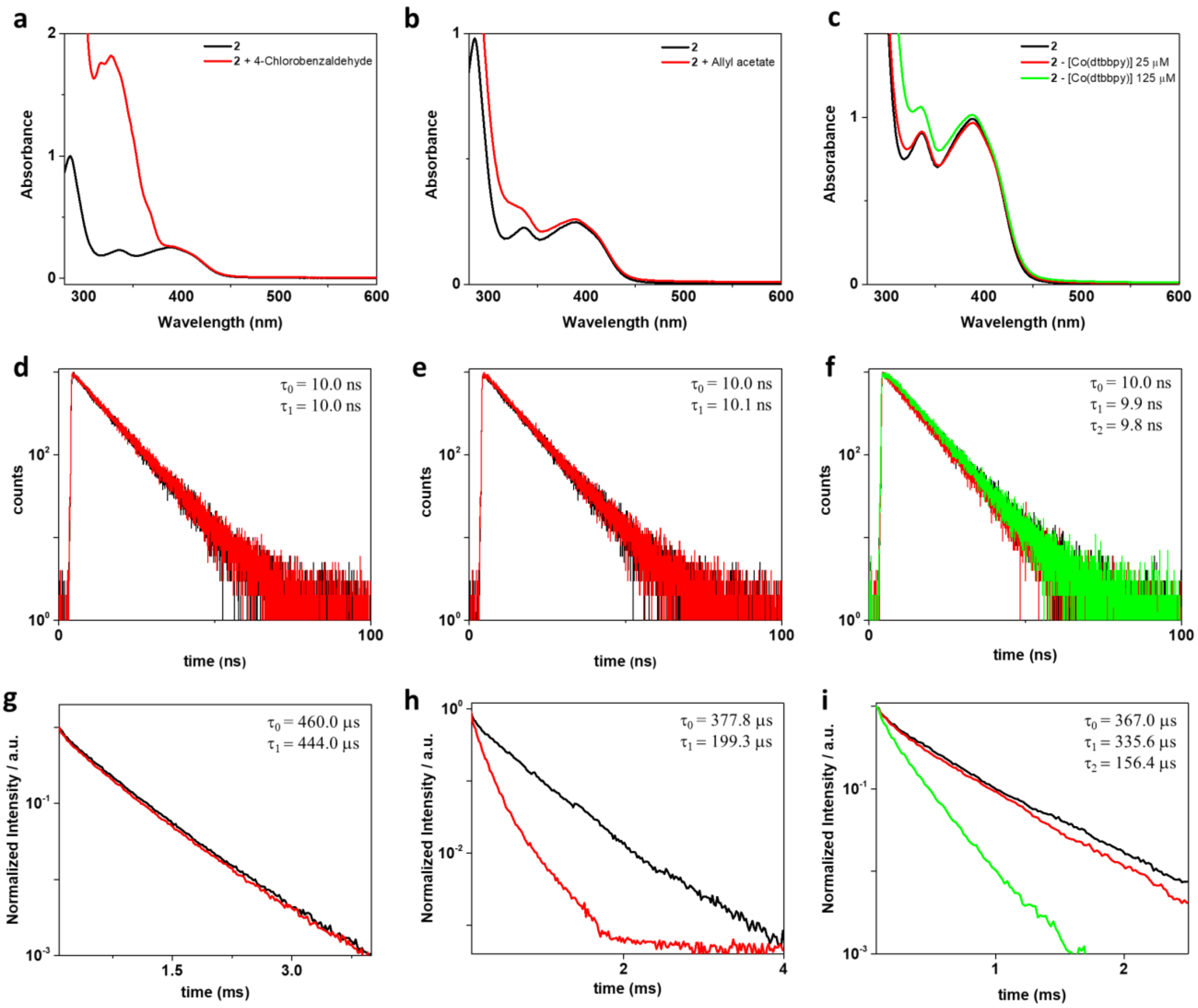

The quenching processes are studied by recording the changes in the lifetime of both prompt and delayed emission of **2** upon addition of 4-chlorobenzaldehyde and allyl acetate at reaction concentrations (0.05 M and 0.15 M respectively) and solution of $CoBr_2$ and dtbbpy at diluted concentrations (25 and 125 μM), as the Co(II) complex absorbs excitation light. Although quenching experiments for **HE** were performed and strong quenching was observed for delayed emission, a reliable value of the Stern-Volmer quenching constant was not obtained. As a result, the quenching efficiency of species under the reaction conditions was not evaluated due to lack of data for **HE**. It was concluded that no quenching of prompt fluorescence of photocatalyst takes place in the presence of any quencher. The quenching constant of the delayed fluorescence of **2** by allyl acetate and Co(II) complex was estimated as 2.5×10^4 $M^{-1}s^{-1}$ and 1×10^8 $M^{-1}s^{-1}$, respectively, and no quenching was observed for 4-chlorobenzaldehyde.

NMR studies of compounds 1 and 2

Figure S4. ^{1}H NMR (400 MHz, $CDCl_3$) of **1**.

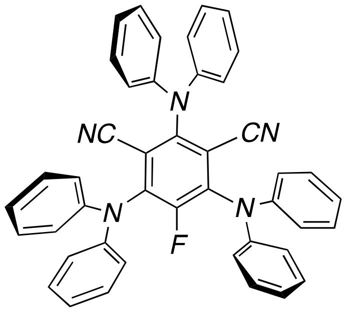

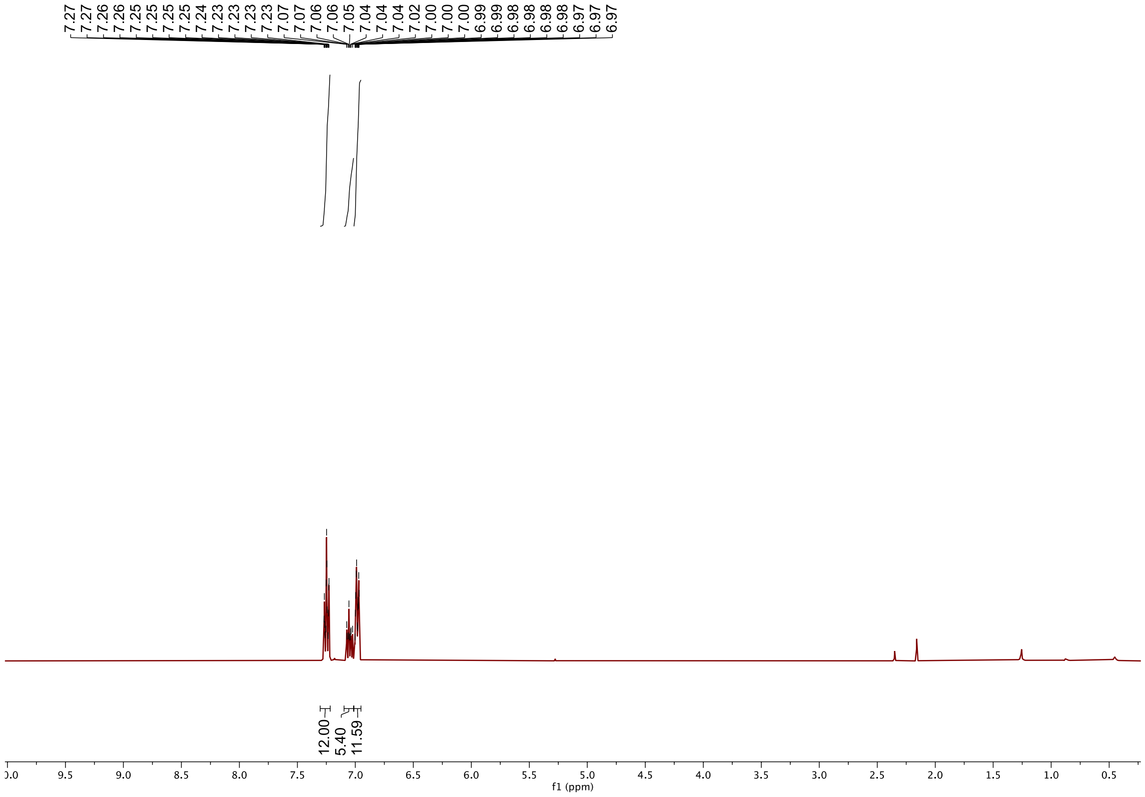

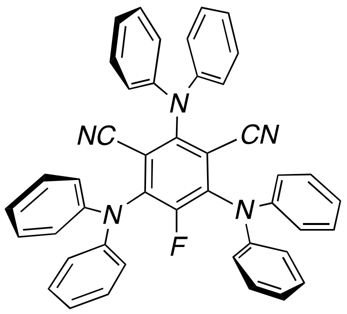

Figure S5. $^{13}C\{1H\}$ NMR (400 MHz, $CDCl_3$) of **1**.

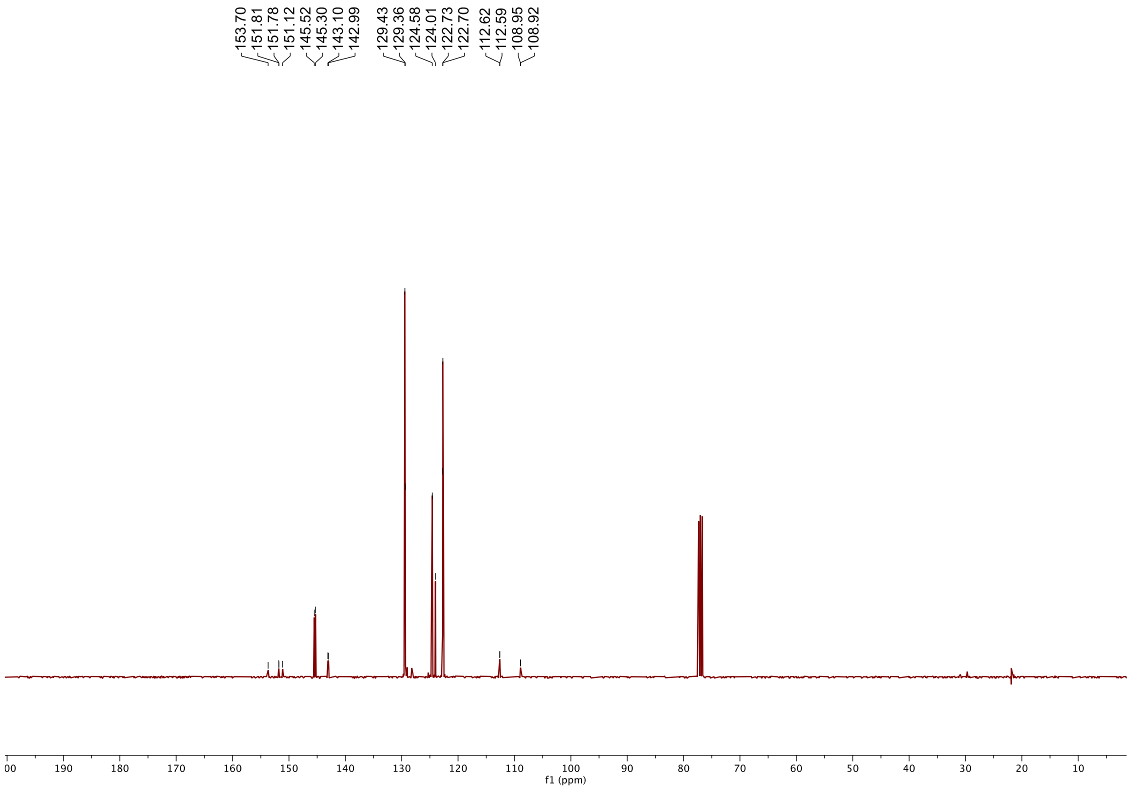

Peaks assignments for 1

Figure S6. Graphical peak assignment for ¹HNMR spectrum of **1**.

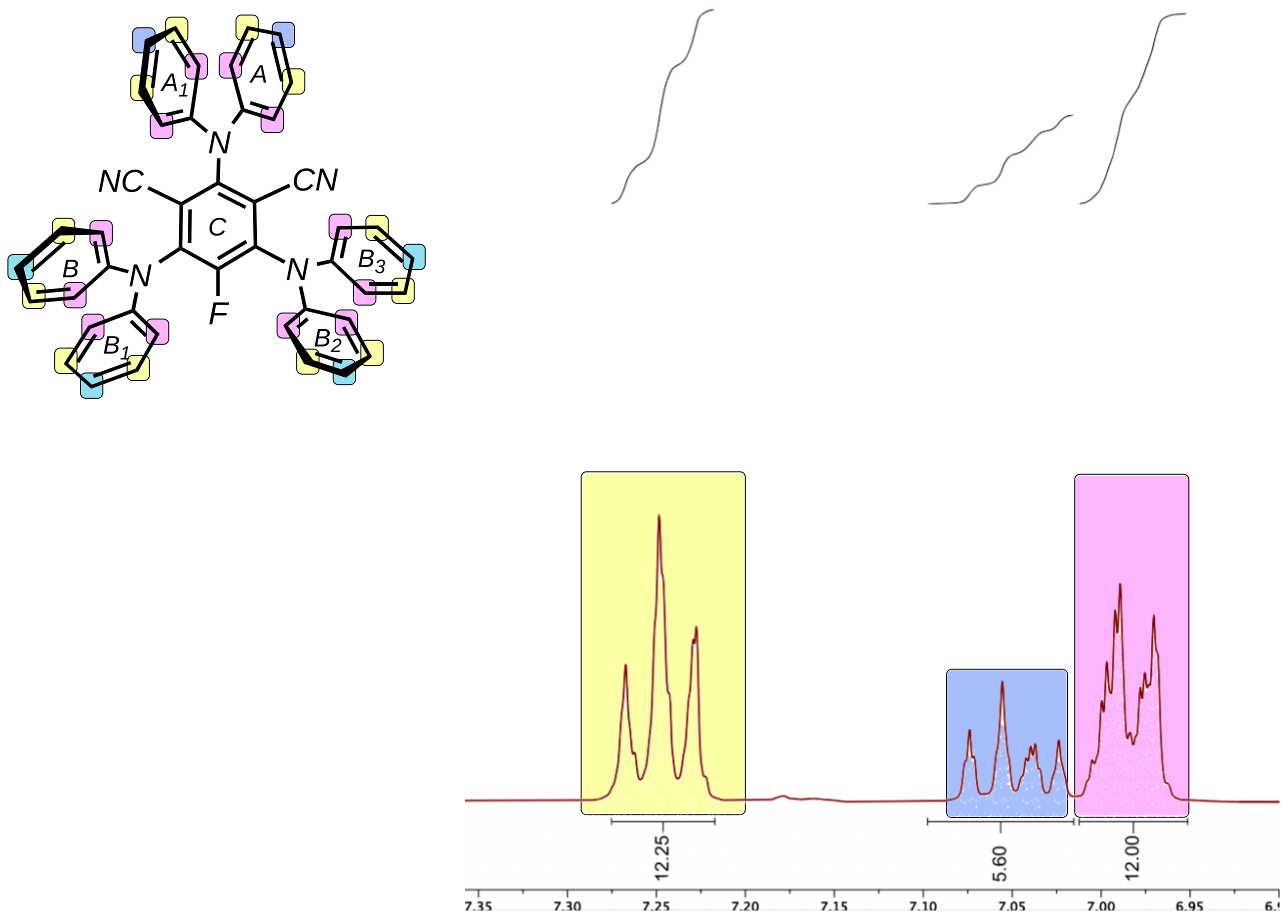

Figure S7. Graphical peak assignment for $^{13}C\{1H\}$ NMR spectrum of **1**.

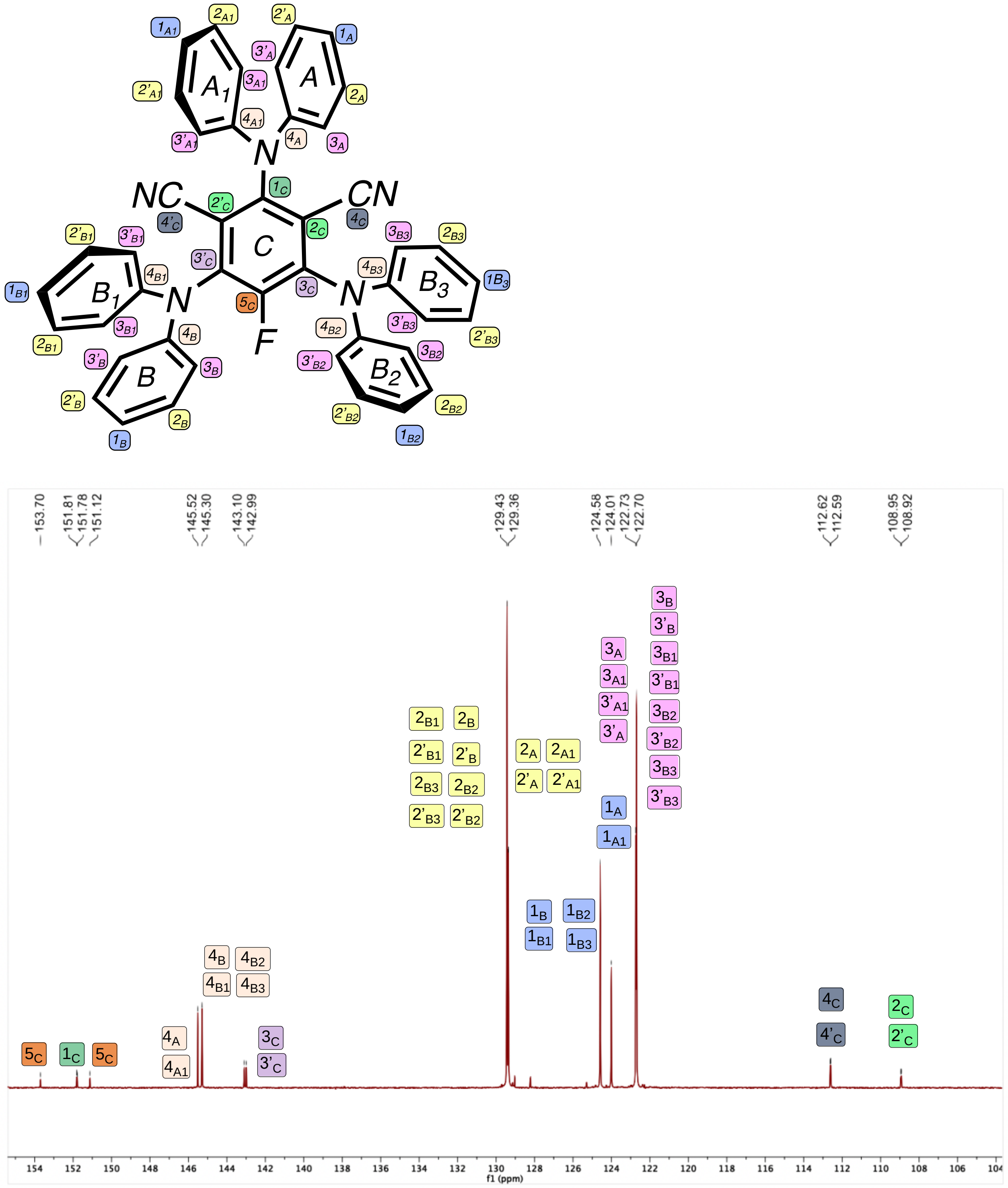

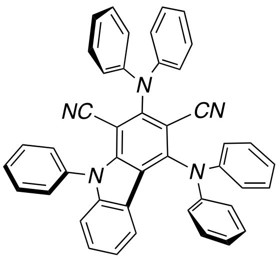

Figure S8. ^{1}H NMR (400 MHz, $CDCl_3$) of **2**.

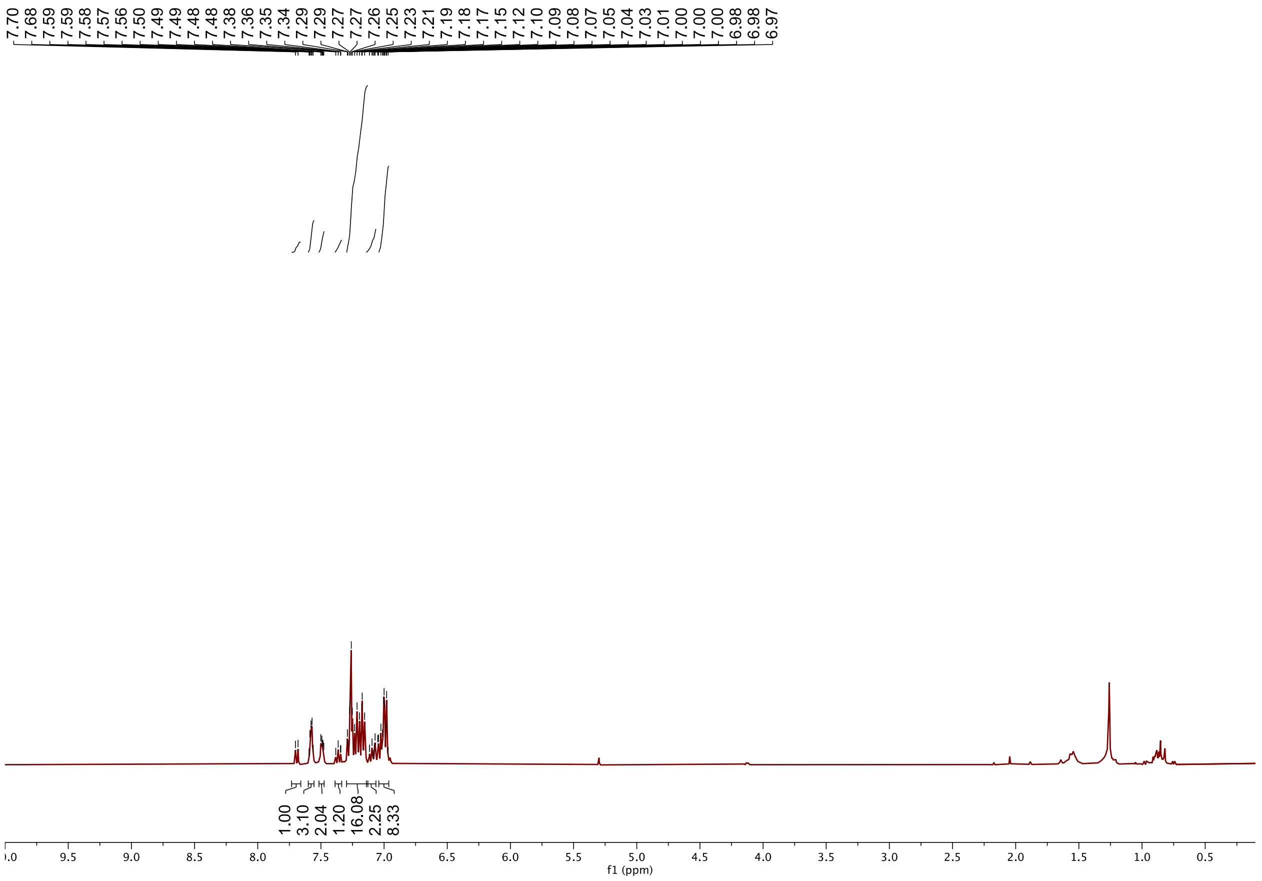

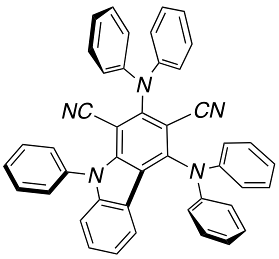

Figure S9. ^{1}H NMR (400 MHz, $CDCl_3$) of **2**.

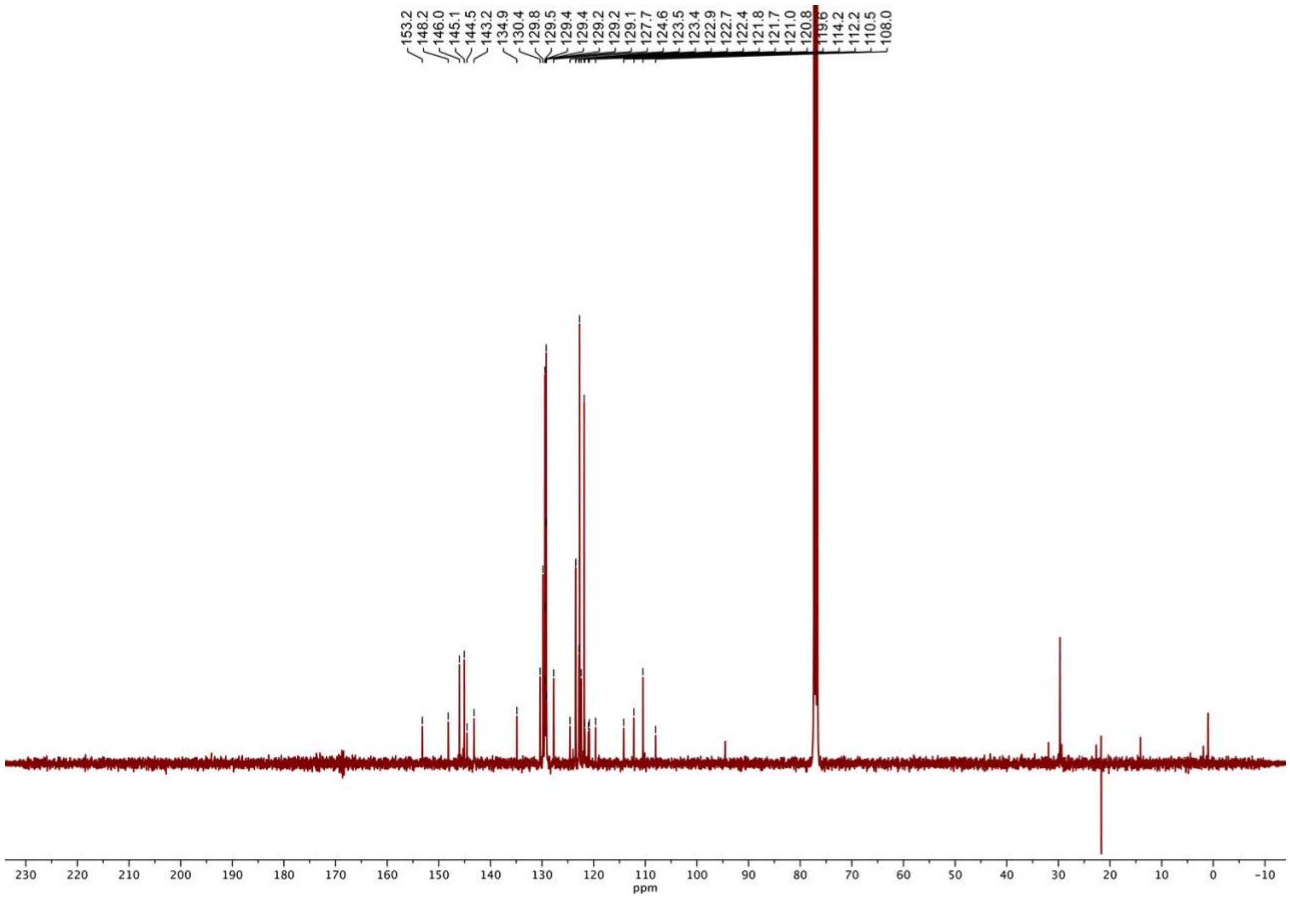

Peaks assignments for 2

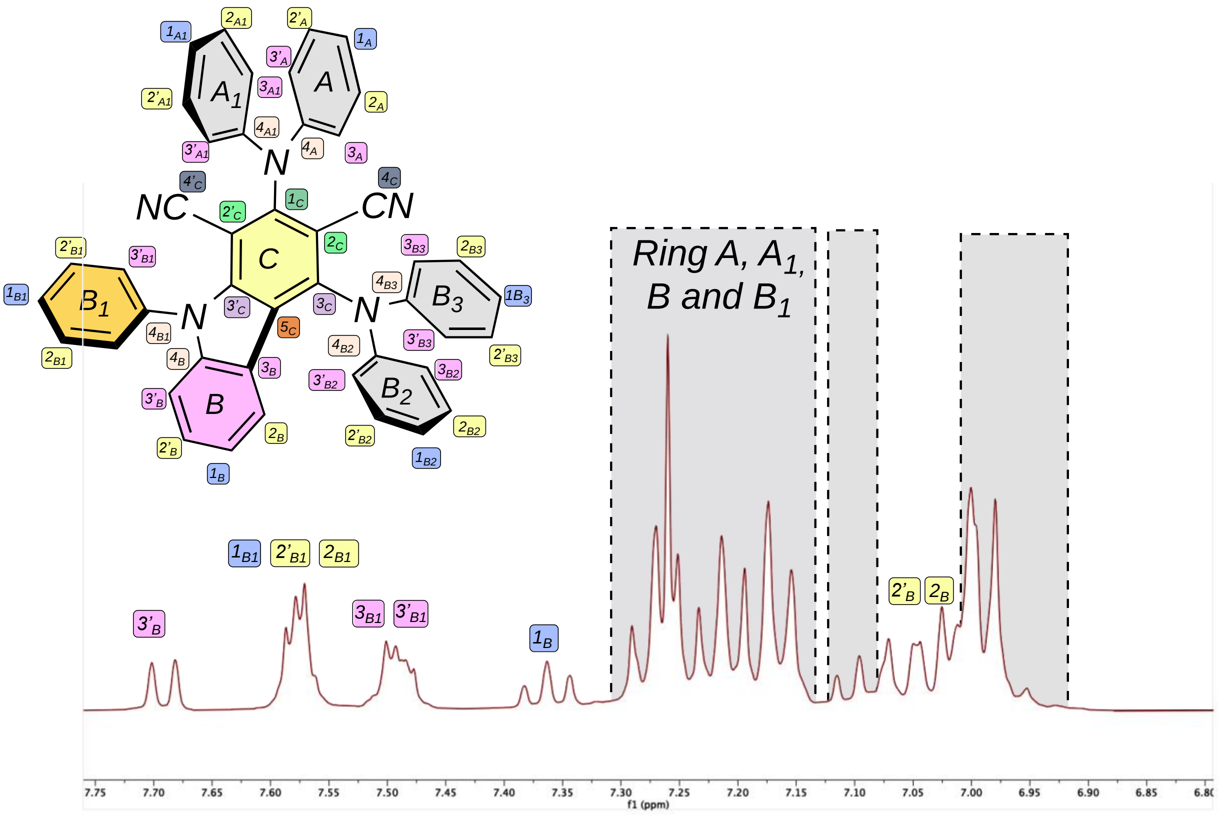

Figure S10. Graphical peak assignment for ^{1}H NMR spectrum of **2**.

Figure S11. Graphical peak assignment for $^{13}C\{1H\}$ NMR: spectrum of **2** (diphenyl amine region zoom).

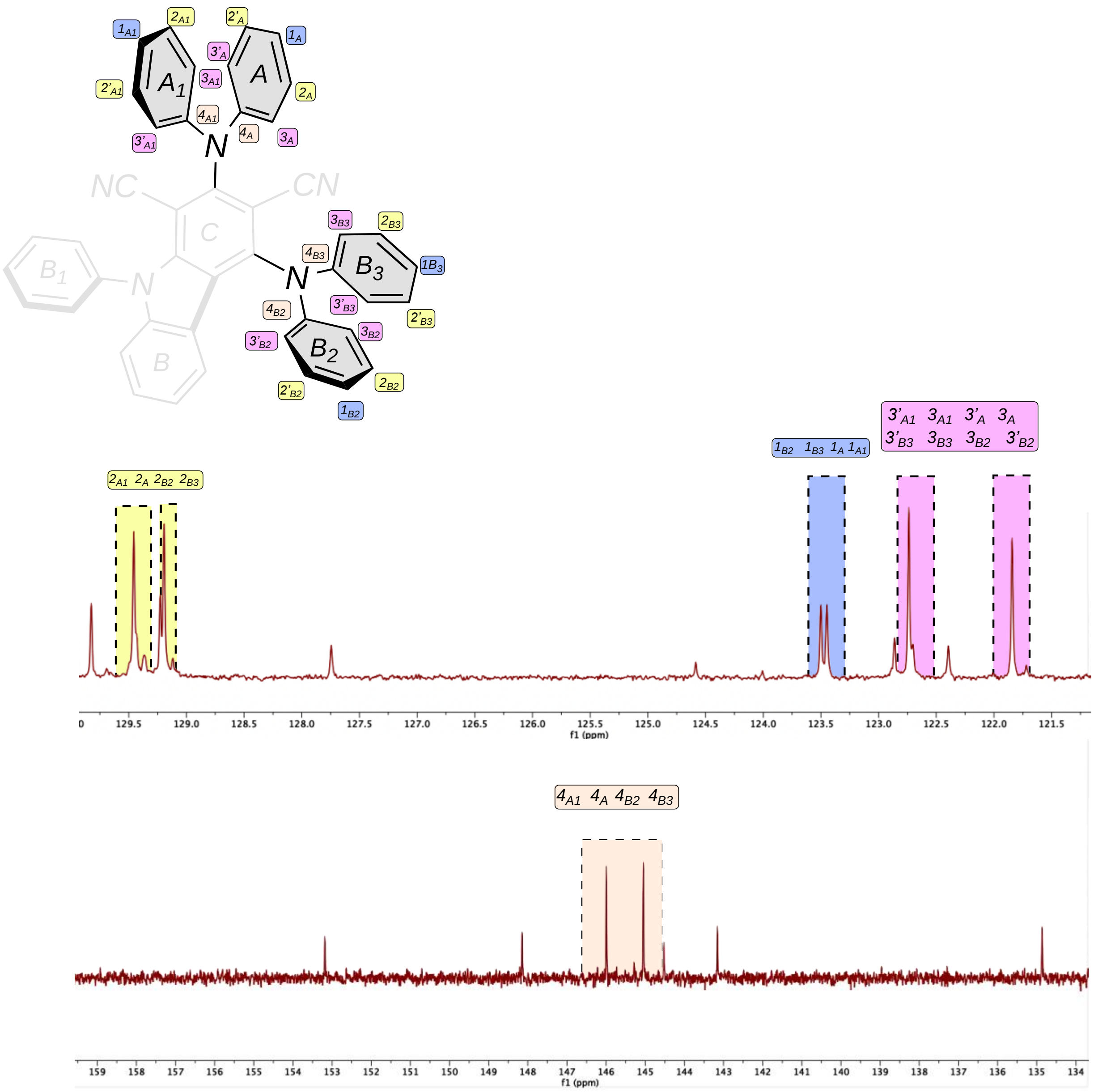

Figure S12. Graphical peak assignment for $^{13}C\{1H\}$ NMR: spectrum of **2** (core region zoom).

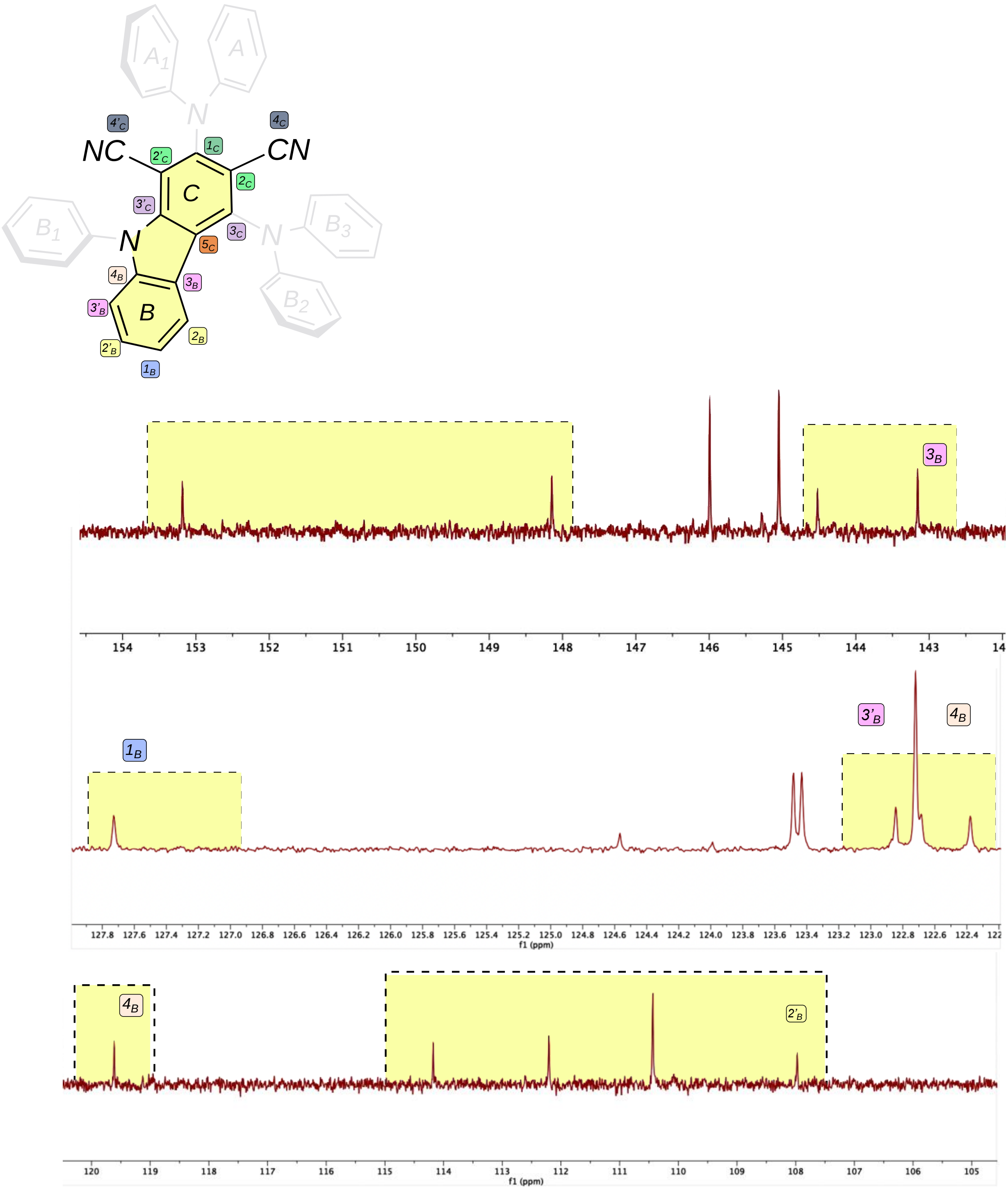

Figure S13. Graphical peak assignment for $^{13}C\{1H\}$ NMR: spectrum of **2** (*N*-phenyl region zoom).

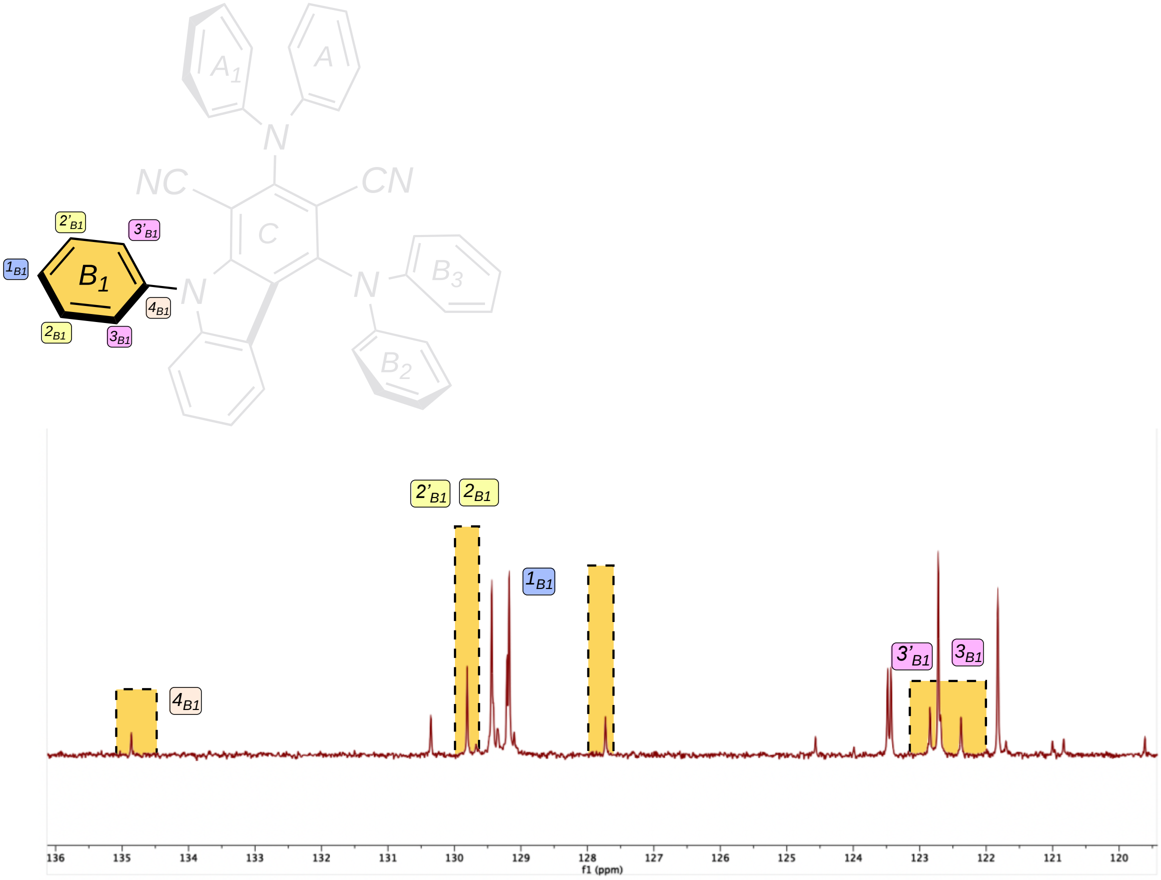

2D NMR spectra

Figure S14. COSY spectrum (400 MHz, $CDCl_3$) of **1**.

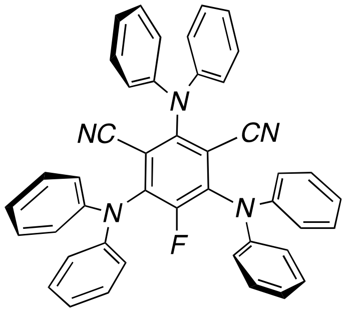

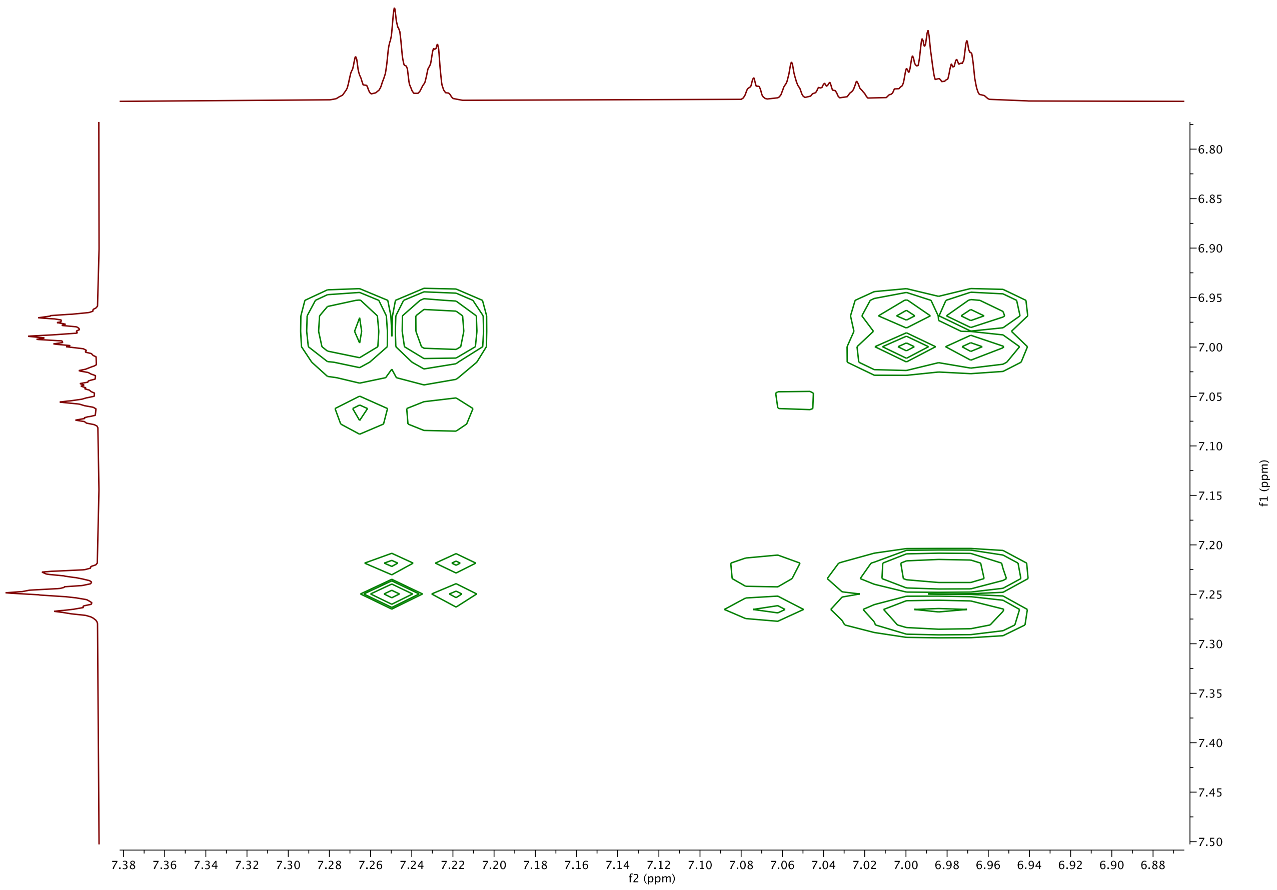

Figure S15. HSQC spectrum (400 MHz, $CDCl_3$) of **1**.

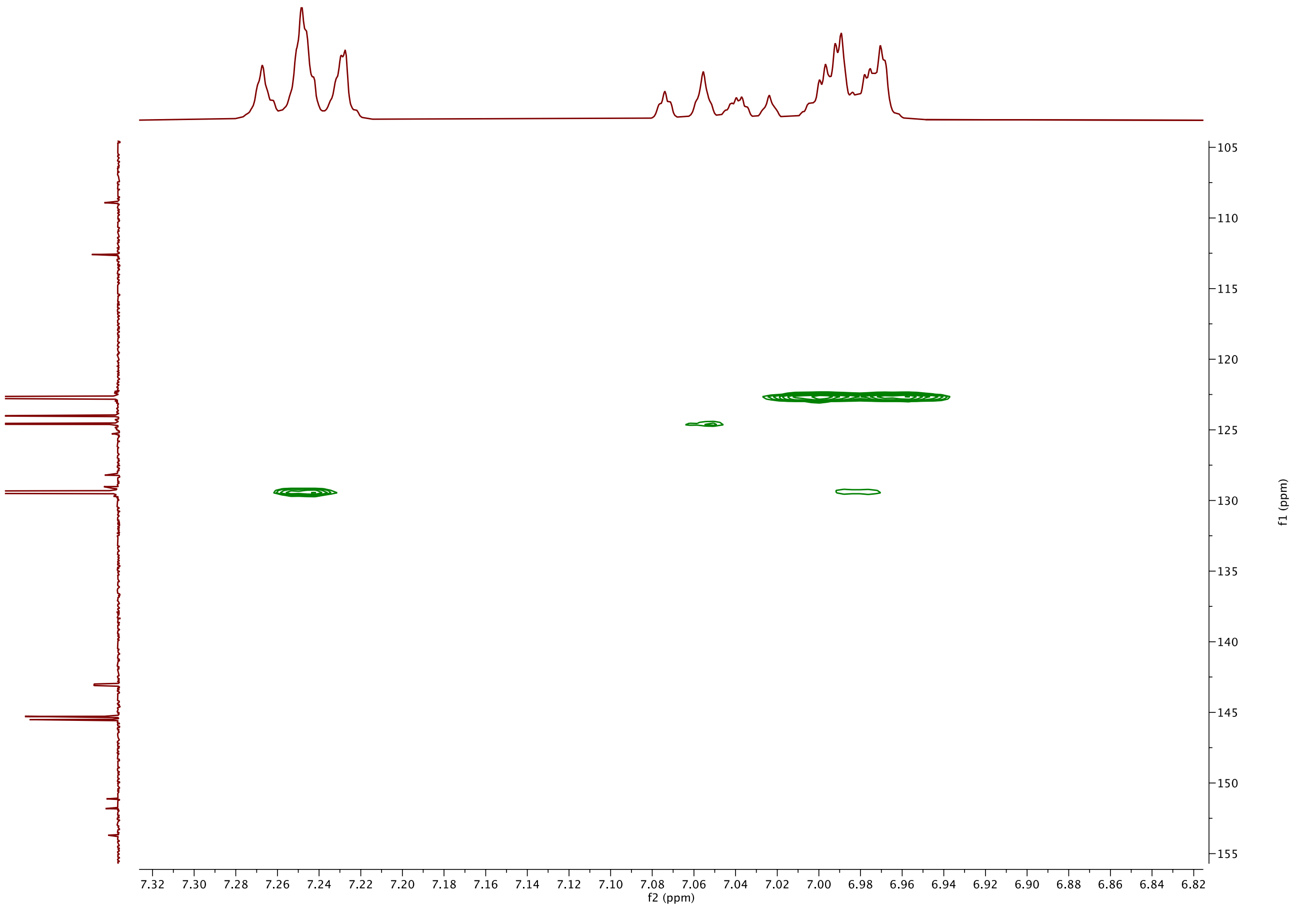

Figure S16. HMBC spectrum (400 MHz, $CDCl_3$) of **1**.

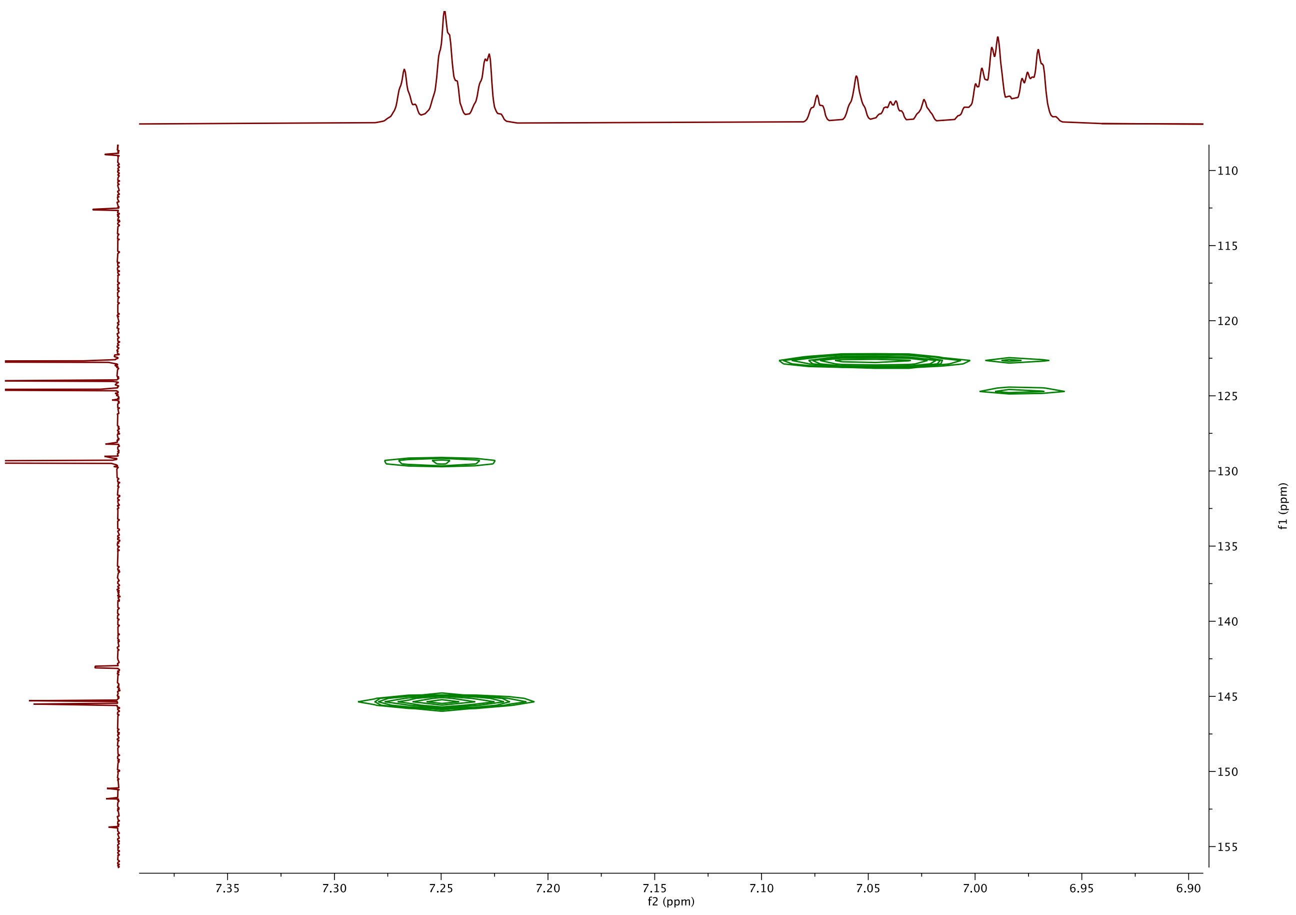

Figure S17. COSY spectrum (400 MHz, CDCl₃) of **2**.

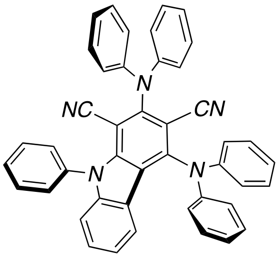

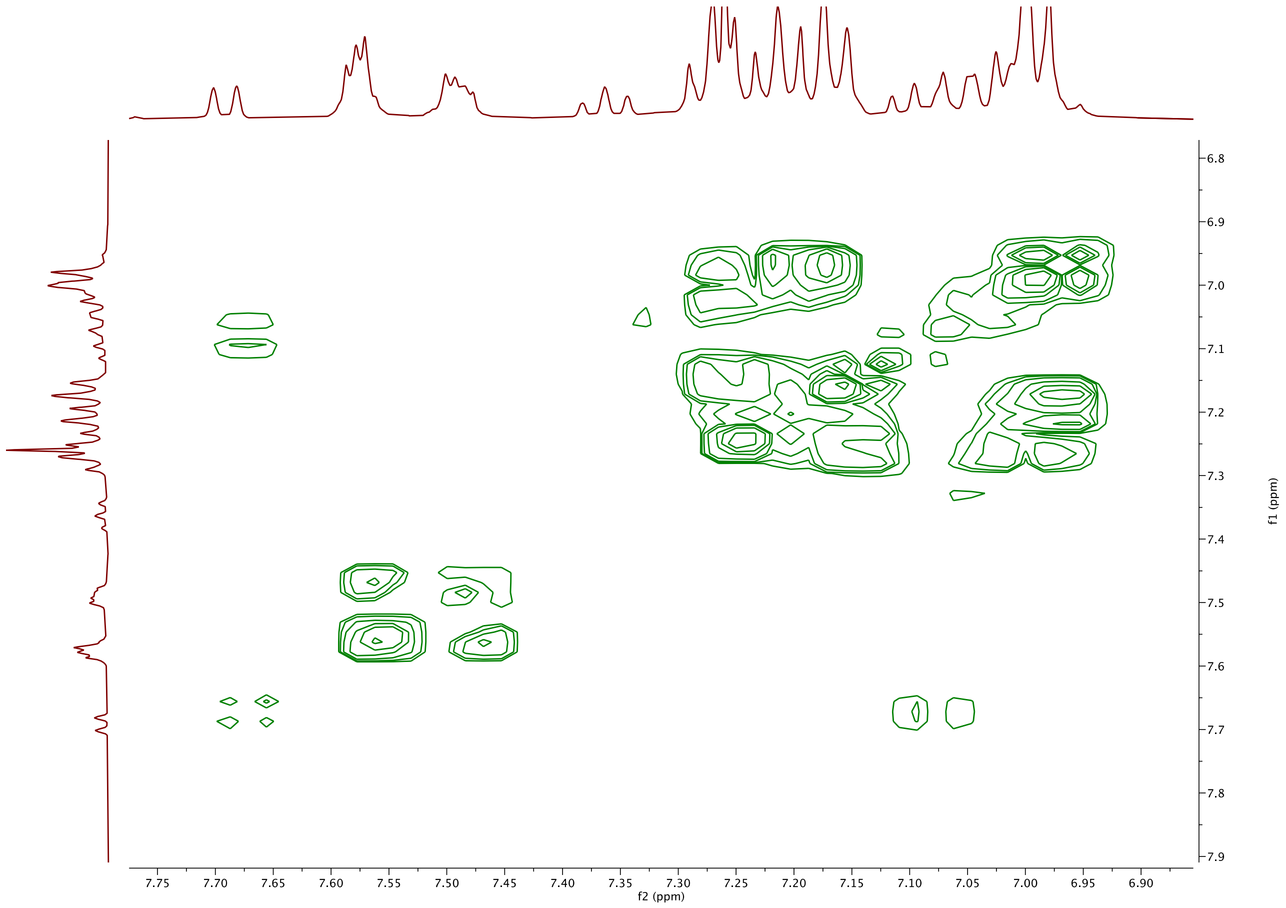

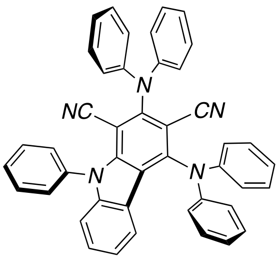

Figure S18. HSQC spectrum (400 MHz, $CDCl_3$) of **2**.

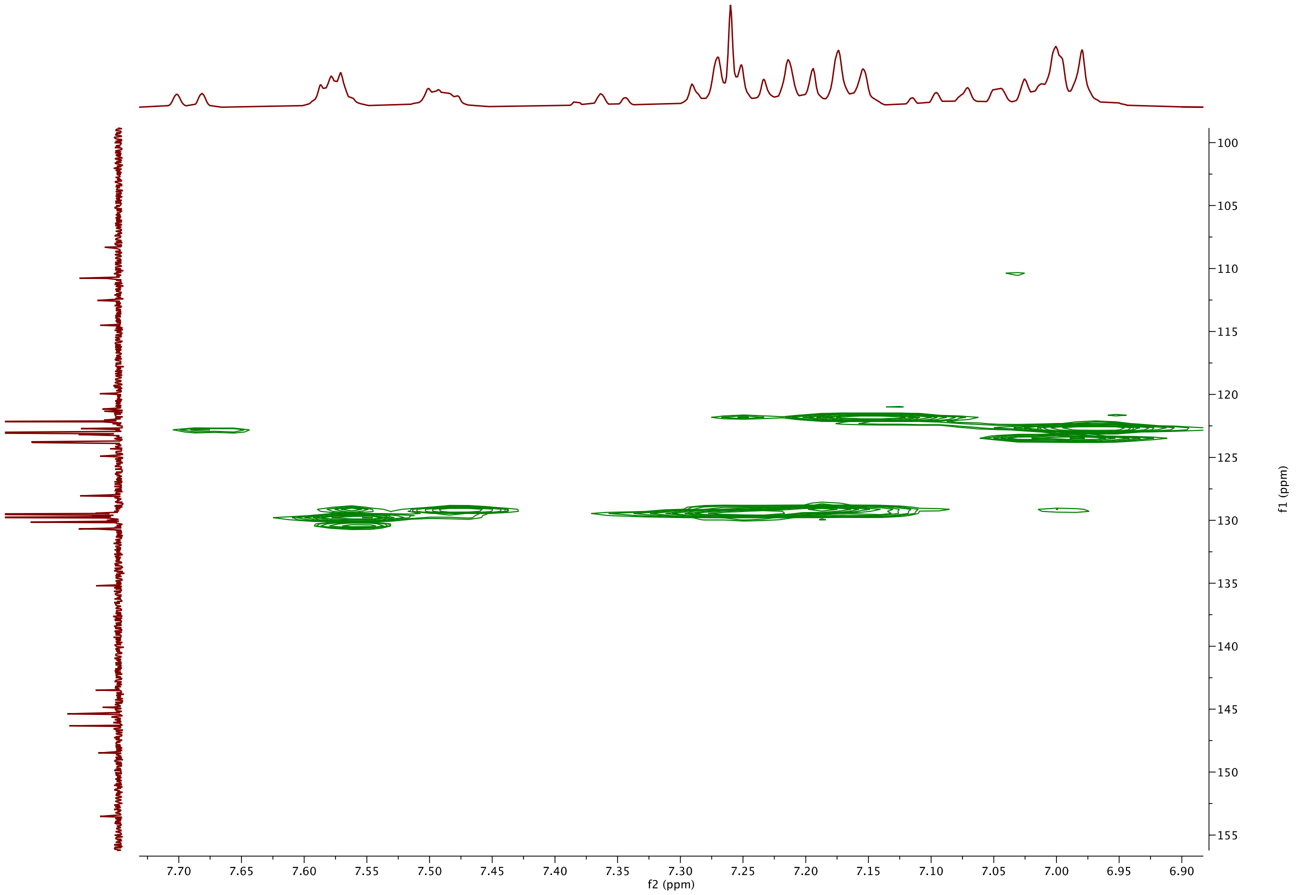

Figure S19. HMBC spectrum (400 MHz, $CDCl_3$) of **2**.

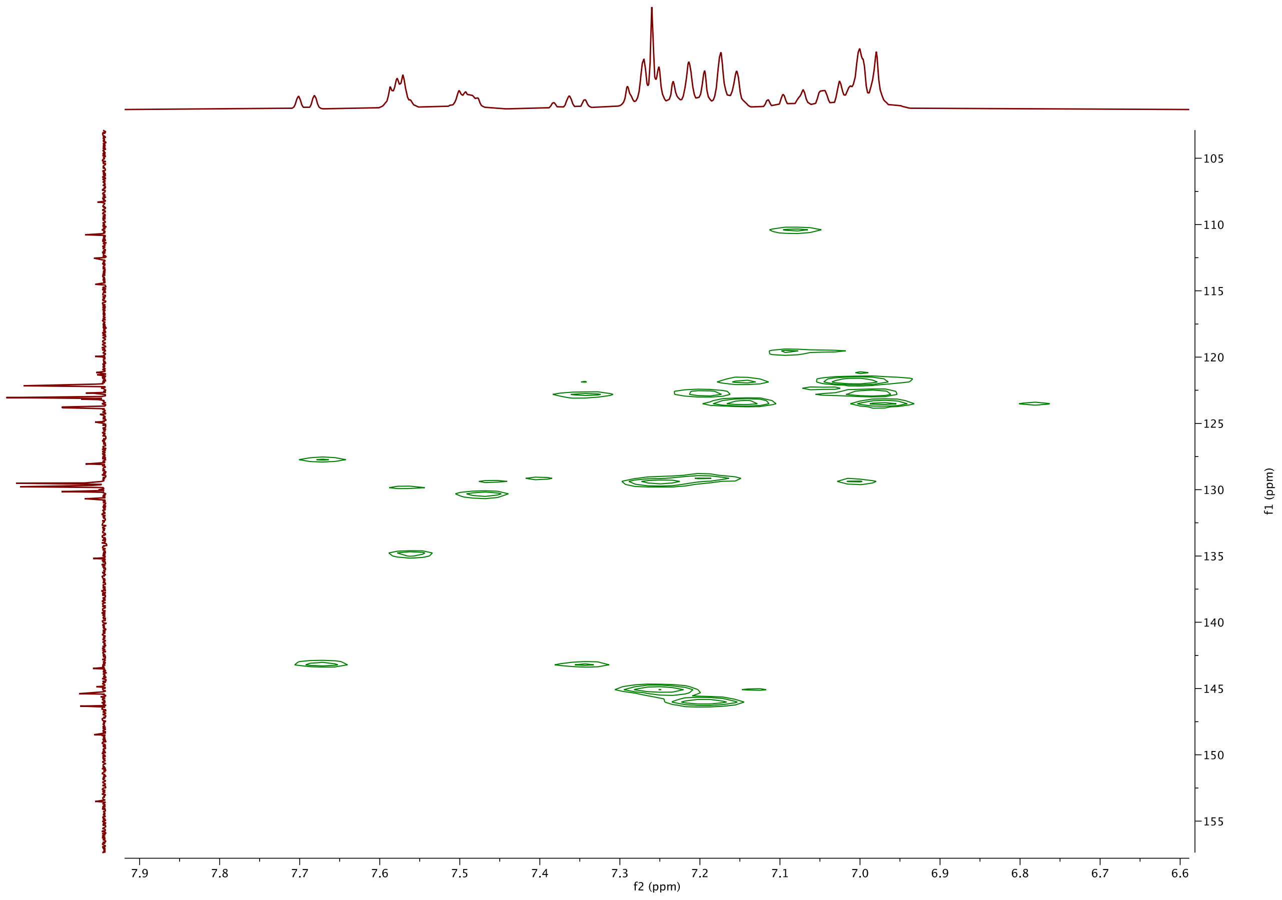

Photoredox reactions

Figure S20. Emission profile of the Kessil® PR160L@456 nm used to irradiate the solutions (form Kessil® website: https://www.kessil.com/science/PR160L.php).

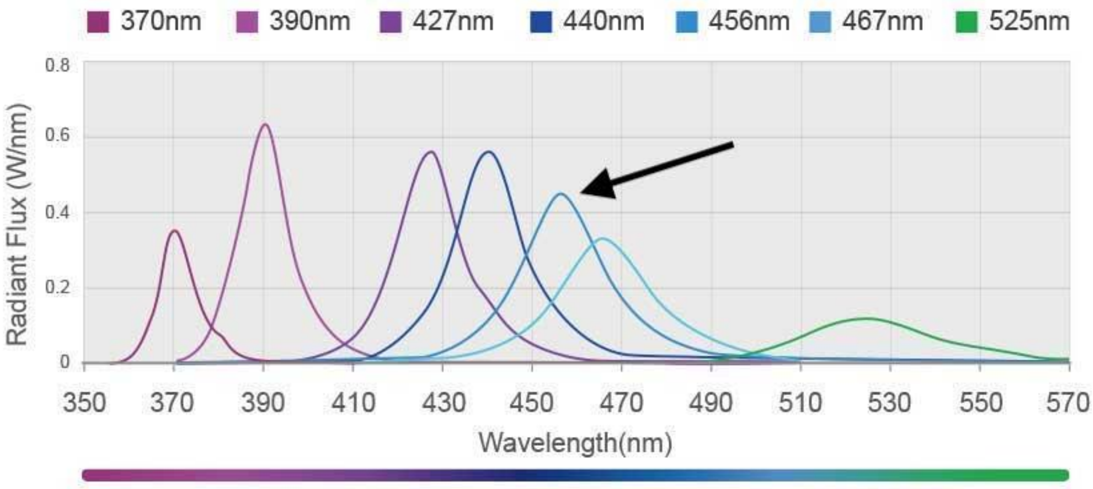

Scheme S1. Unsuitable substrate for the photoredox allylation of aldehyde promoted by cobalt.

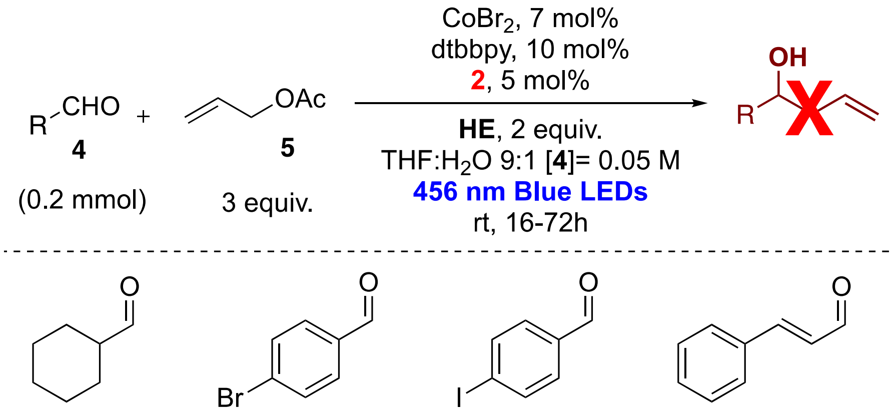

Reaction set-up

Figure S21. Reaction set-up with Kessil® PR160L@456 nm lamp. Reaction flasks were positioned approximatively at 18 cm from the light source.

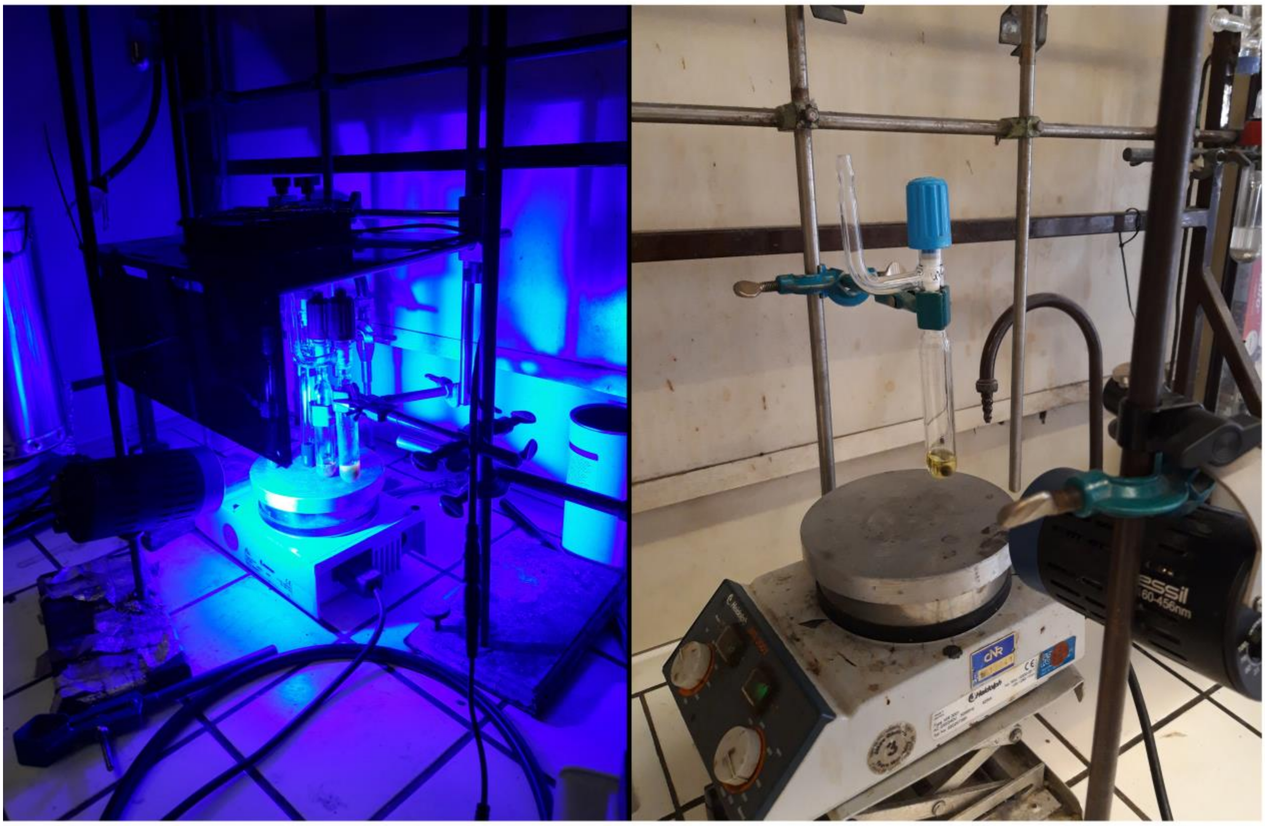

Table S1. Condition screening for photoredox allylation of 4-chlorobenzaldehyde promoted by cobalt.

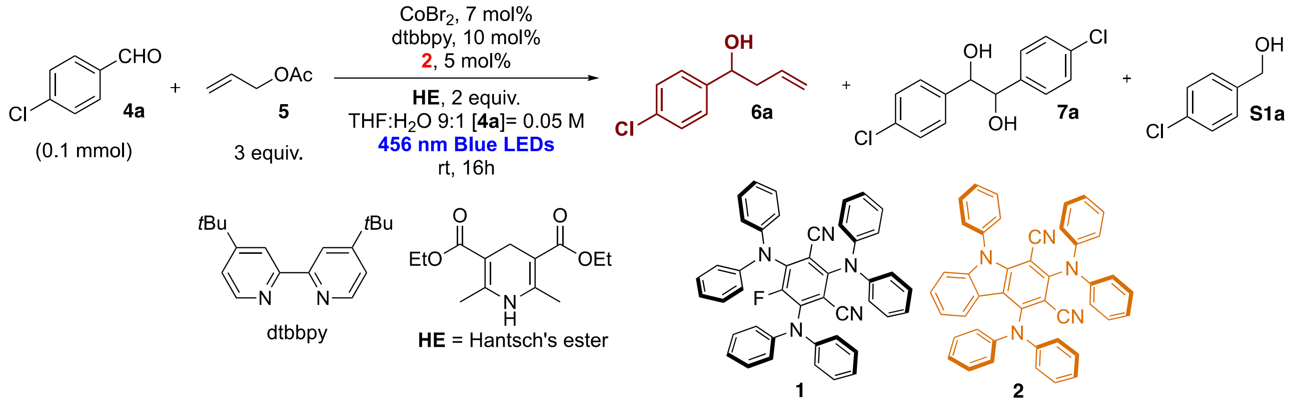

Entry[a]	Deviation from standard conditions	Conversion[b]	6a/7a/S1a[b]
1	$CoCO_3$ hydrate instead of $CoBr_2$	78	62/30/8
2	$Co(acac)_2{\cdot}H_2O$ instead of $CoBr_2$	17	100/0/0
3	$Co(OAc)_2{\cdot}4H_2O$ instead of $CoBr_2$	89	100/0/0
4	$Co(OAc)_2{\cdot}4H_2O$, **1** instead of **2**	92	48/41/11
5	$CoCl_2{\cdot}6H_2O$ instead of $CoBr_2$	>99	1/99/0
6	1 mmol scale and $Co(OAc)_2{\cdot}4H_2O$ instead of $CoBr_2$	80	74(50)/18/8
7	None	96	90(87)/4/2
8	No $CoBr_2$	N.R.[c]	-
9	No photocatalyst	N.R.[c]	-
10	No Light	N.R.[c]	-
11	No dtbbpy	>99	10/90[d]/0
12	No HE	N.R.[c]	-
13	**1** instead of **2**	>99	2/97[d]/1
14	THF as solvent	>99	10/60[d]/30
15	**1** instead of **2** and THF as solvent	96	12/69[d]/19

[a] All the reactions were carried out under irradiation with Kessil® 40W 456 nm Blue LED. [b] Determined by ^{1}H NMR analysis on the reaction crude. Isolated yields after chromatographic purification are given in parentheses. [c] N.R. = No reaction. [d] 1:1 ratio of *meso*:*d/l* compounds was observed by ^{1}H NMR analysis on the reaction crude.

Copies of NMR spectra for homoallylic alcohols

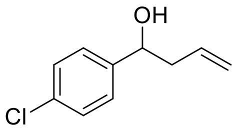

6a

[1]**H NMR** (400 MHz, $CDCl_3$)

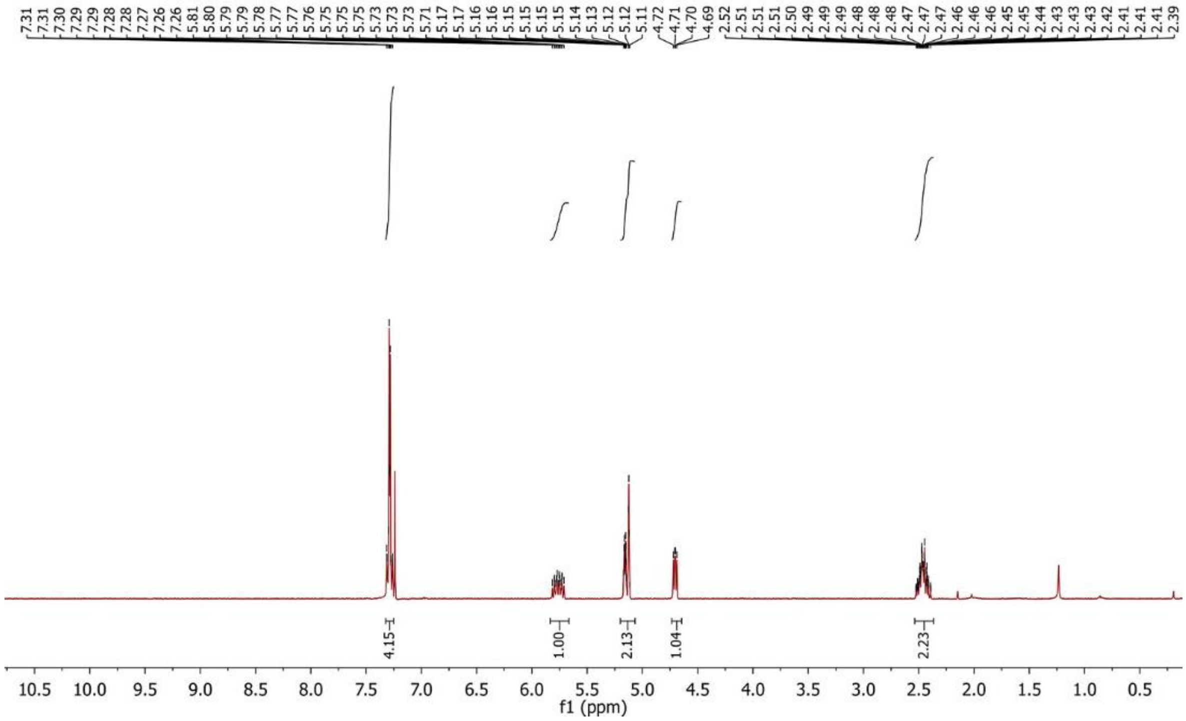

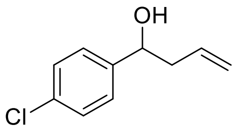

$^{13}C\{^1H\}$ NMR (100 MHz, $CDCl_3$)

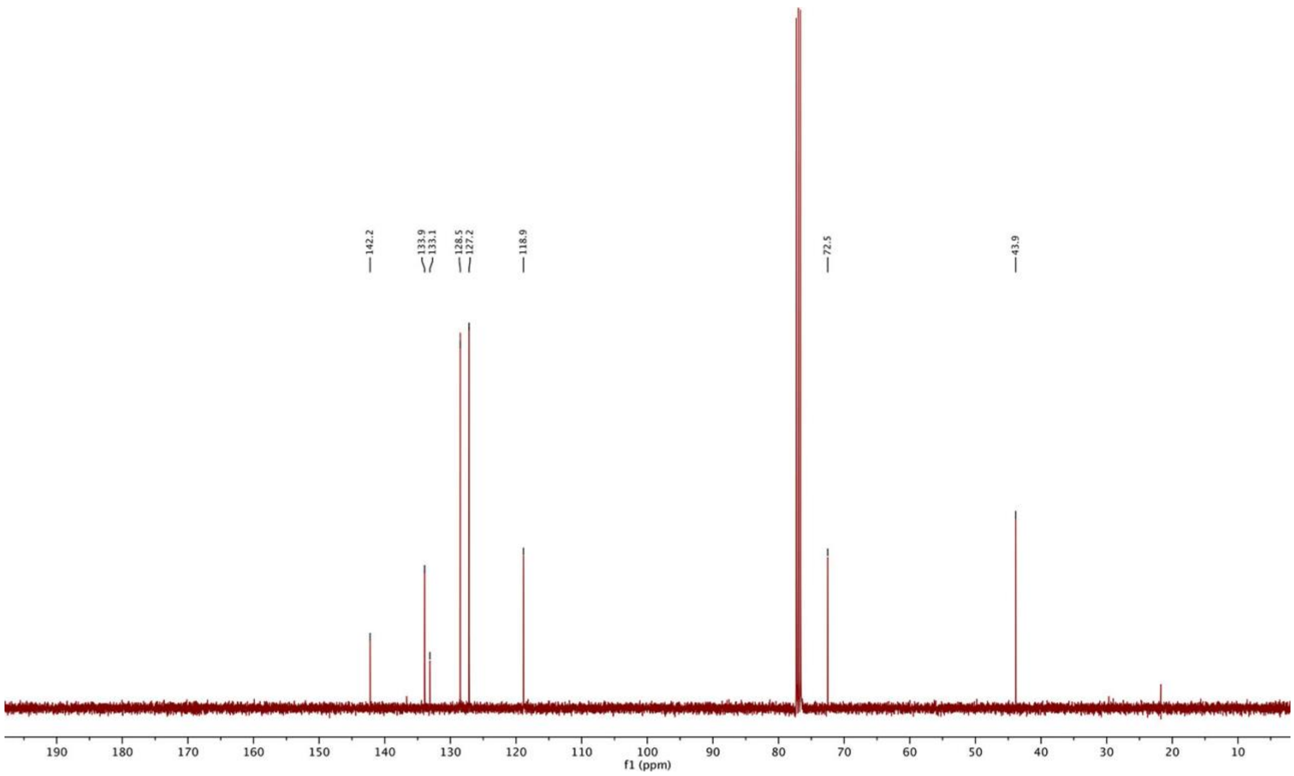

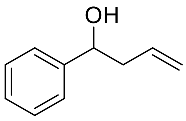

6b

^{1}H NMR (400 MHz, $CDCl_3$)

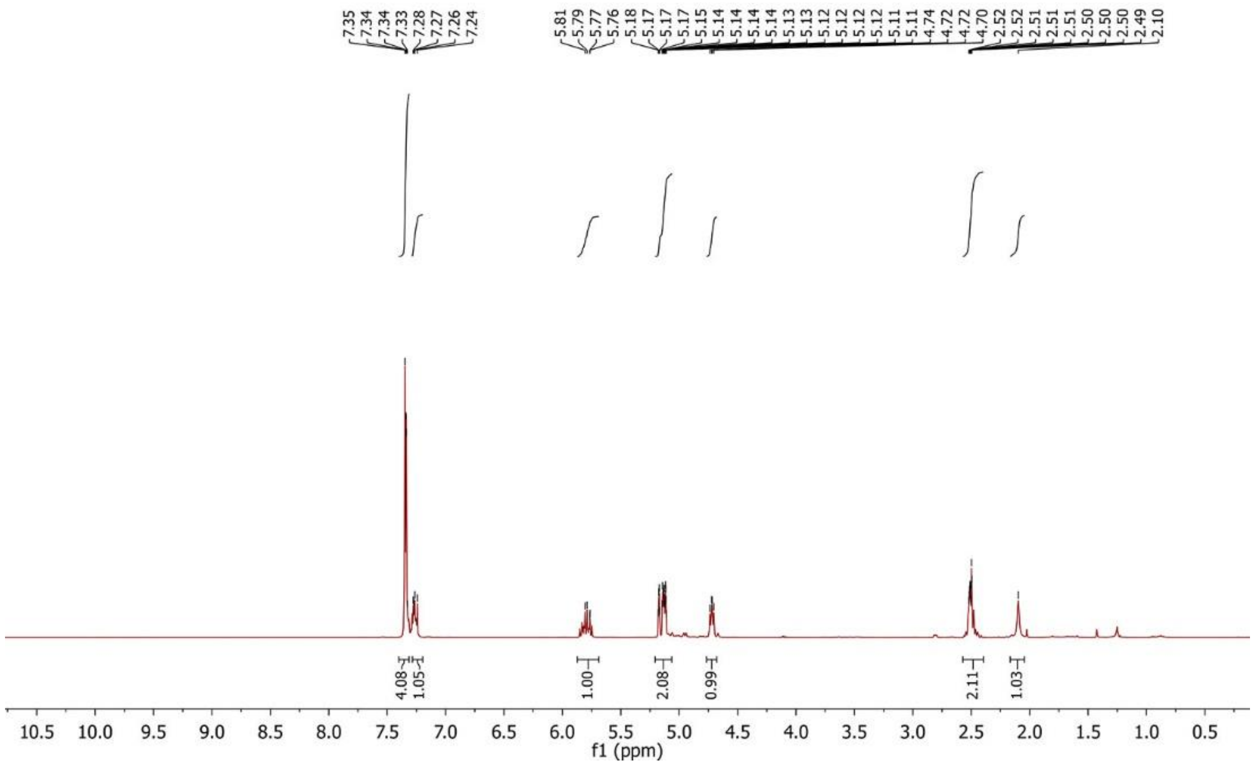

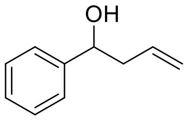

6b

^{13}C{^{1}H} NMR (100 MHz, $CDCl_3$)

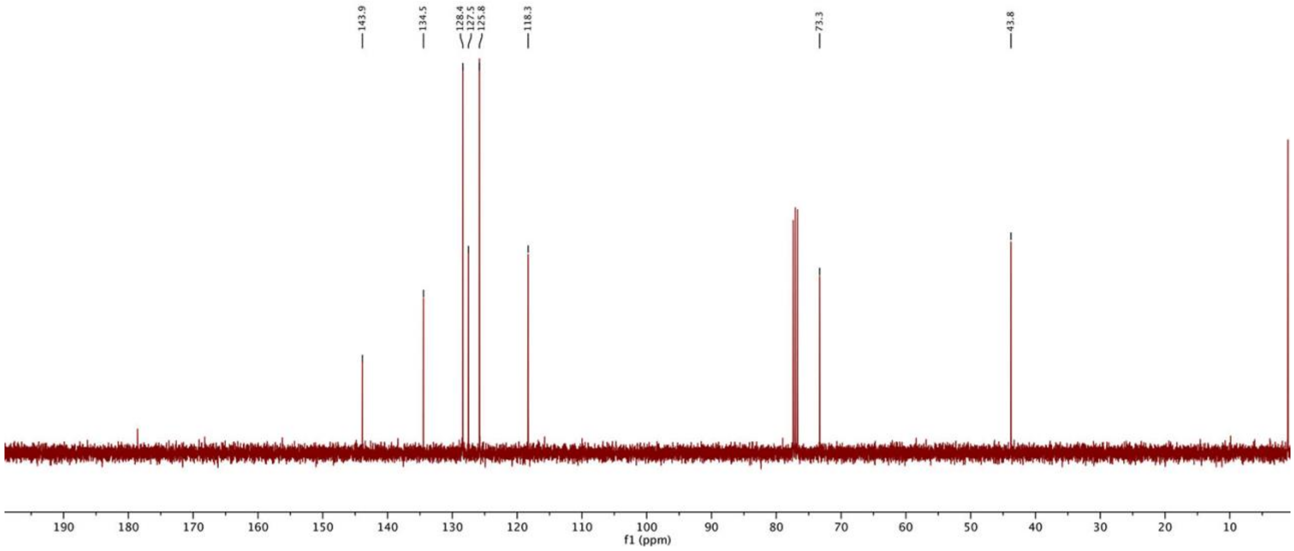

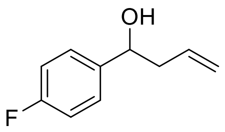

6c

^{1}H NMR (400 MHz, $CDCl_3$)

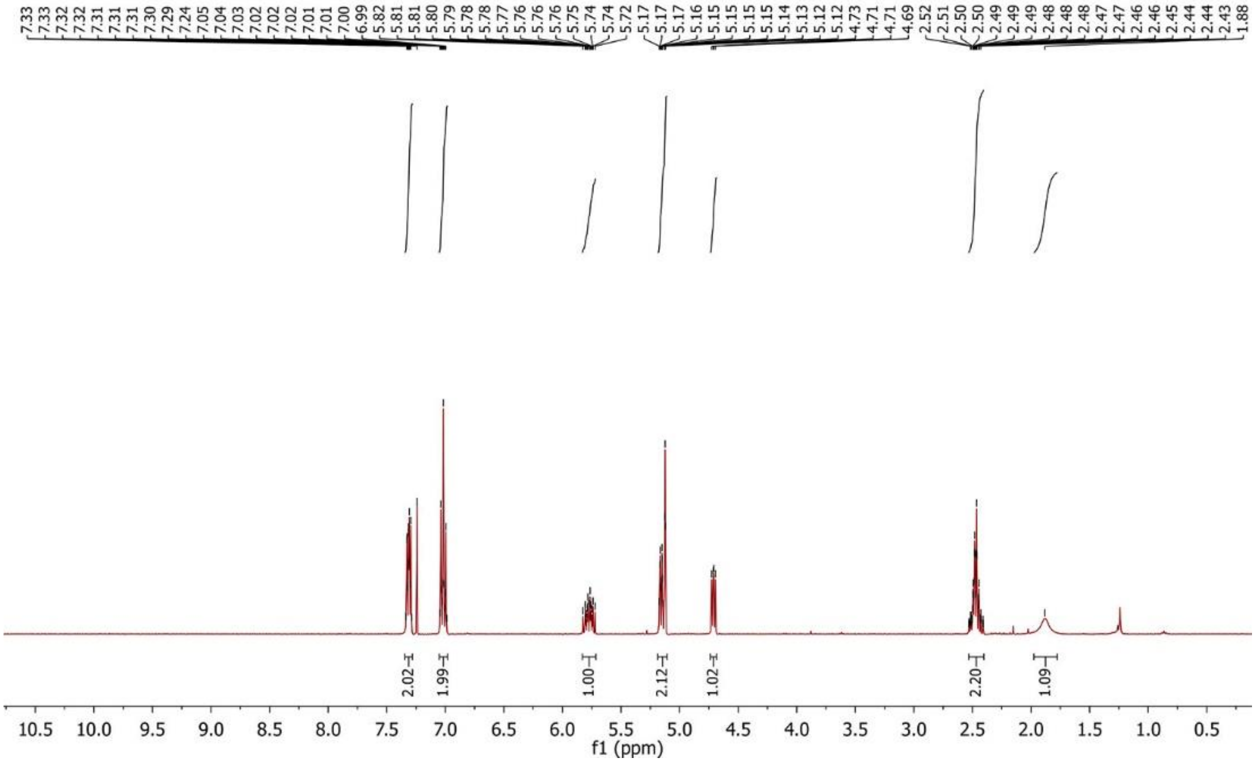

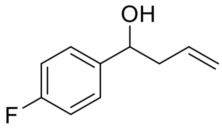

6c

$^{13}C\{^{1}H\}$ NMR (100 MHz, $CDCl_3$)

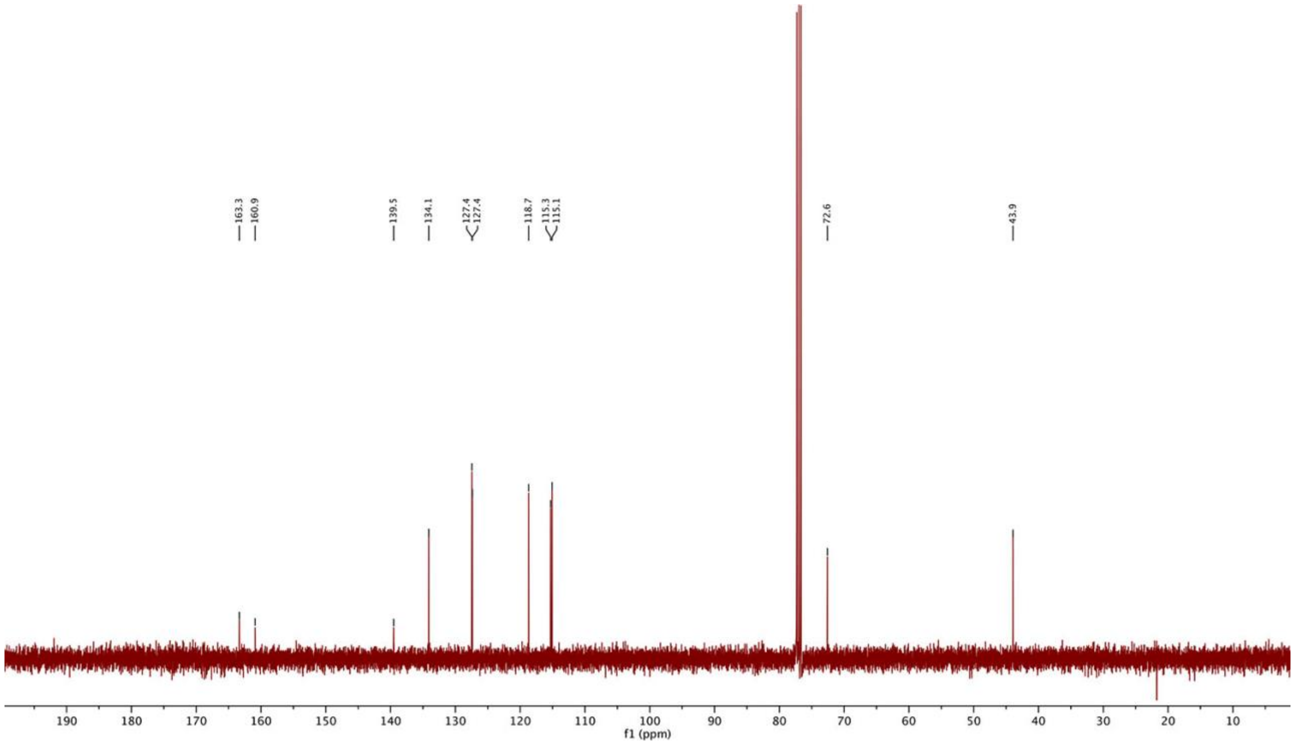

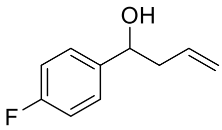

6c

^{19}F NMR (377 MHz, $CDCl_3$)

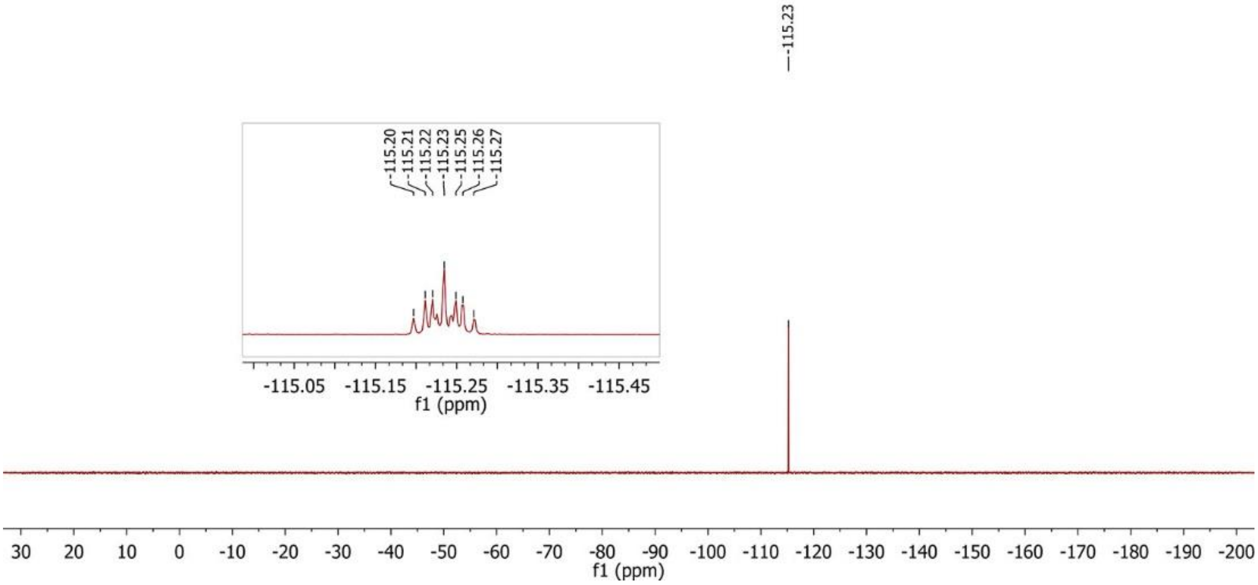

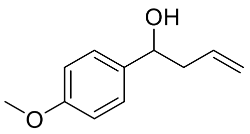

6d

[1]**H NMR** (400 MHz, $CDCl_3$)

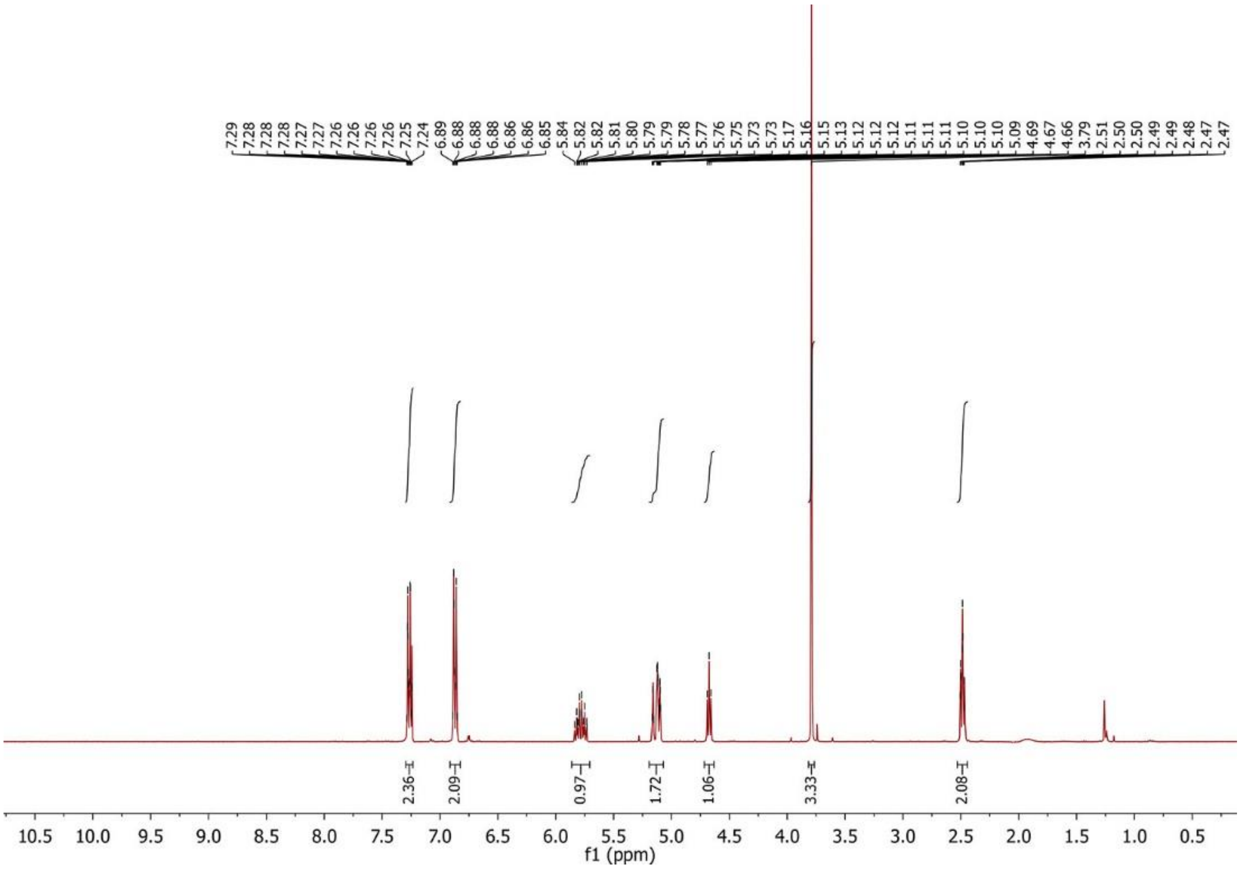

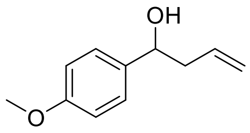

6d

$^{13}C\{^1H\}$ NMR (100 MHz, $CDCl_3$)

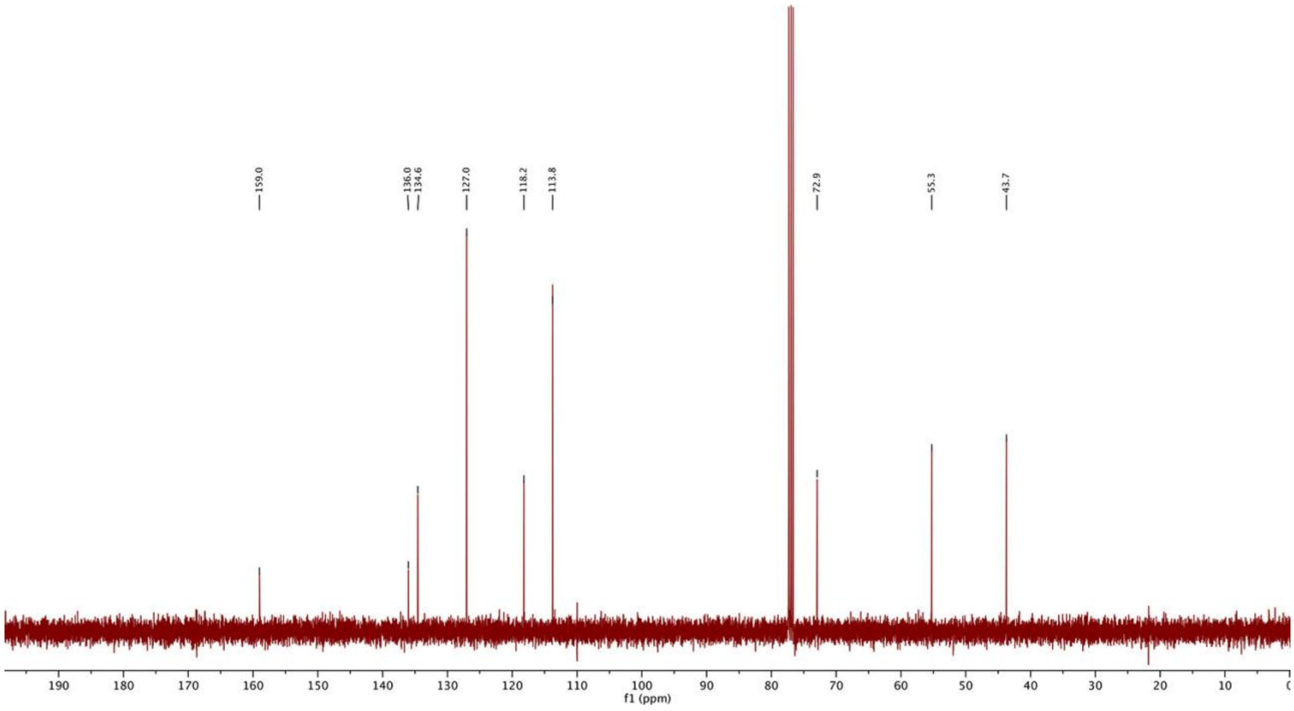

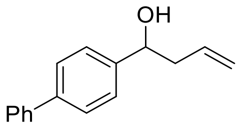

6e

^{1}H NMR (400 MHz, $CDCl_3$)

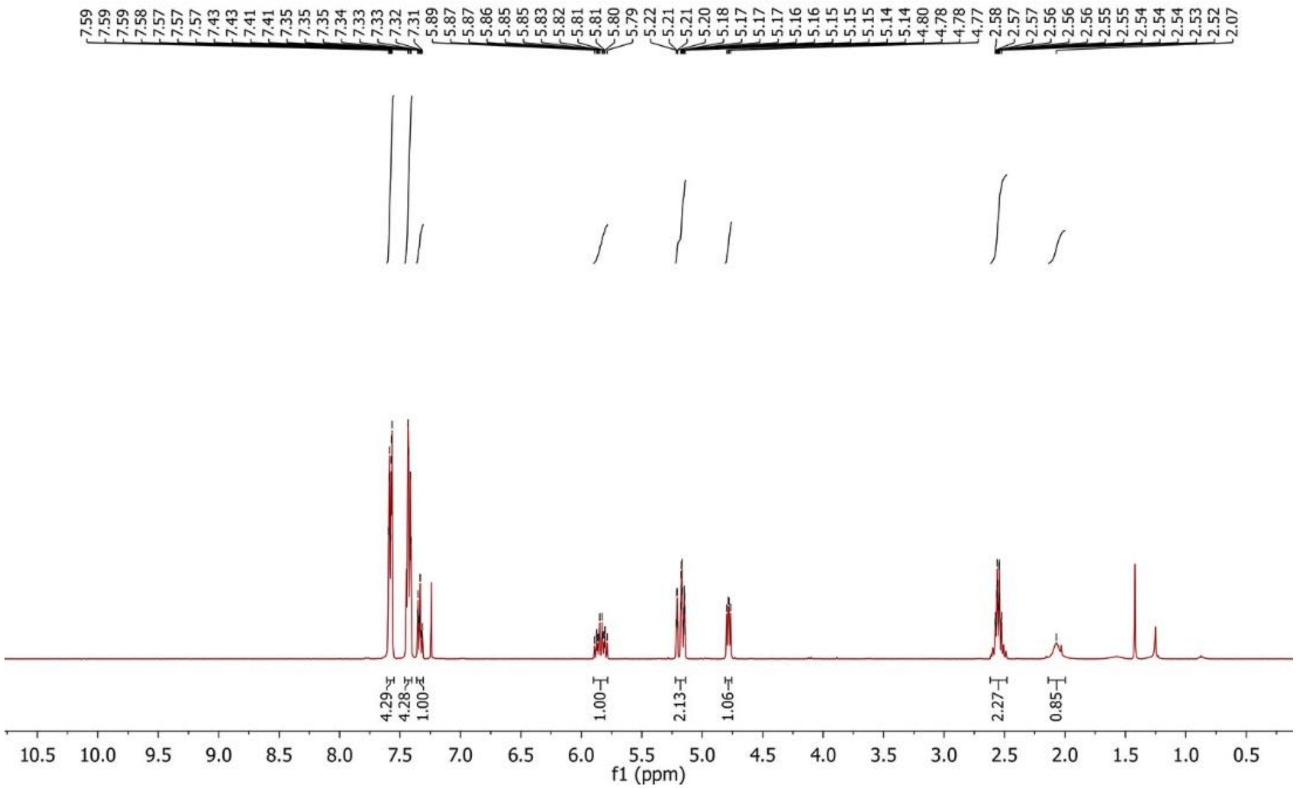

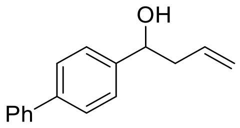

6e

$^{13}C\{^{1}H\}$ NMR (100 MHz, $CDCl_3$)

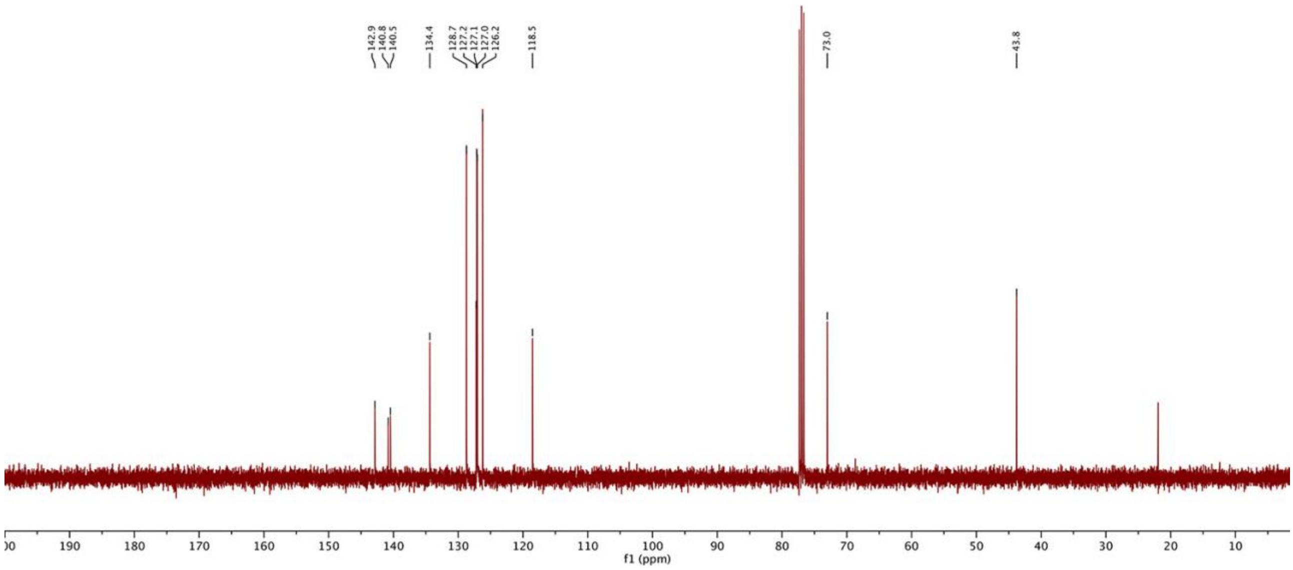

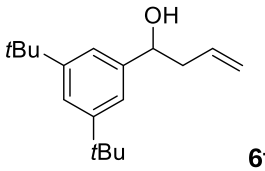

6f

^{1}H NMR (400 MHz, $CDCl_3$)

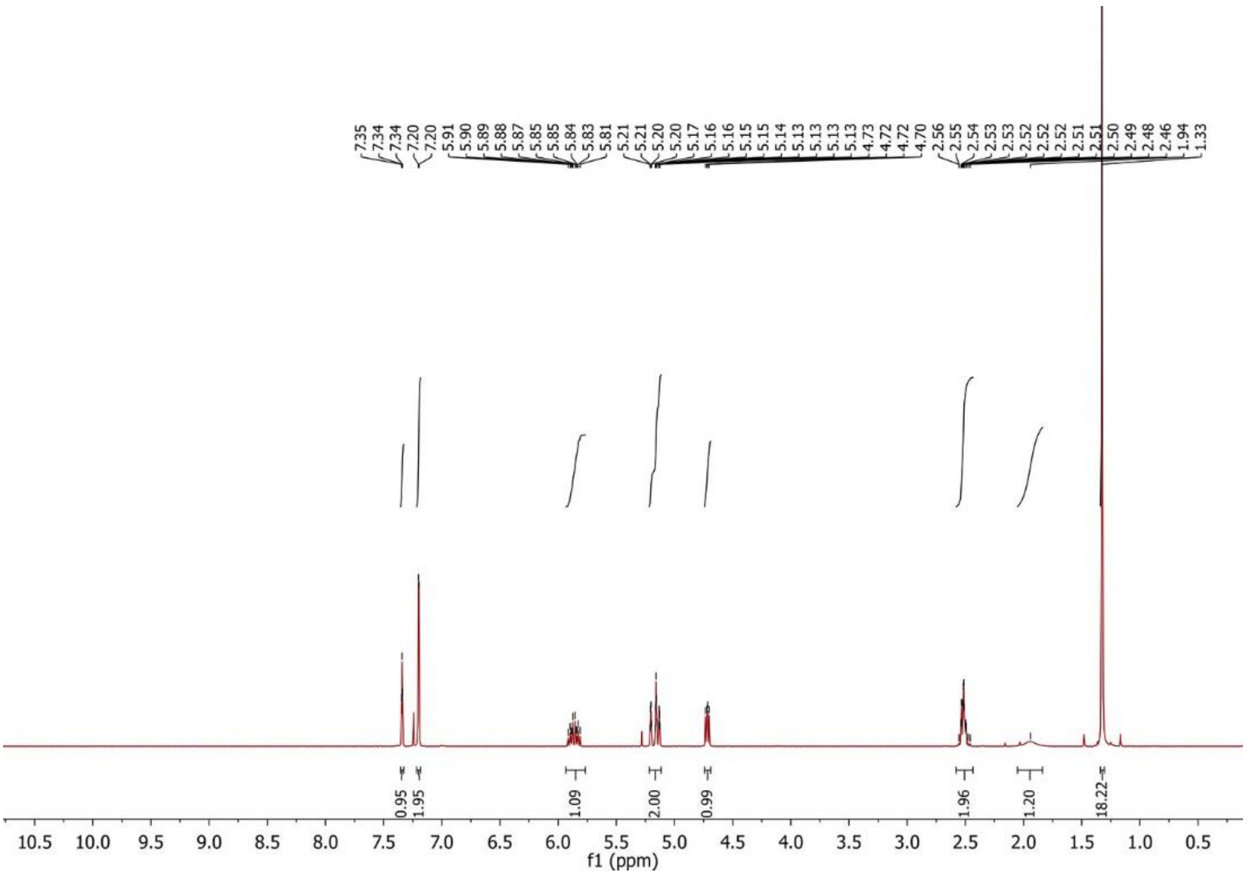

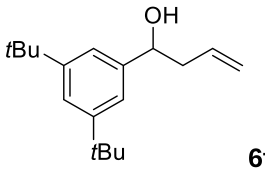

6f

^{13}C{^{1}H} NMR (100 MHz, $CDCl_3$)

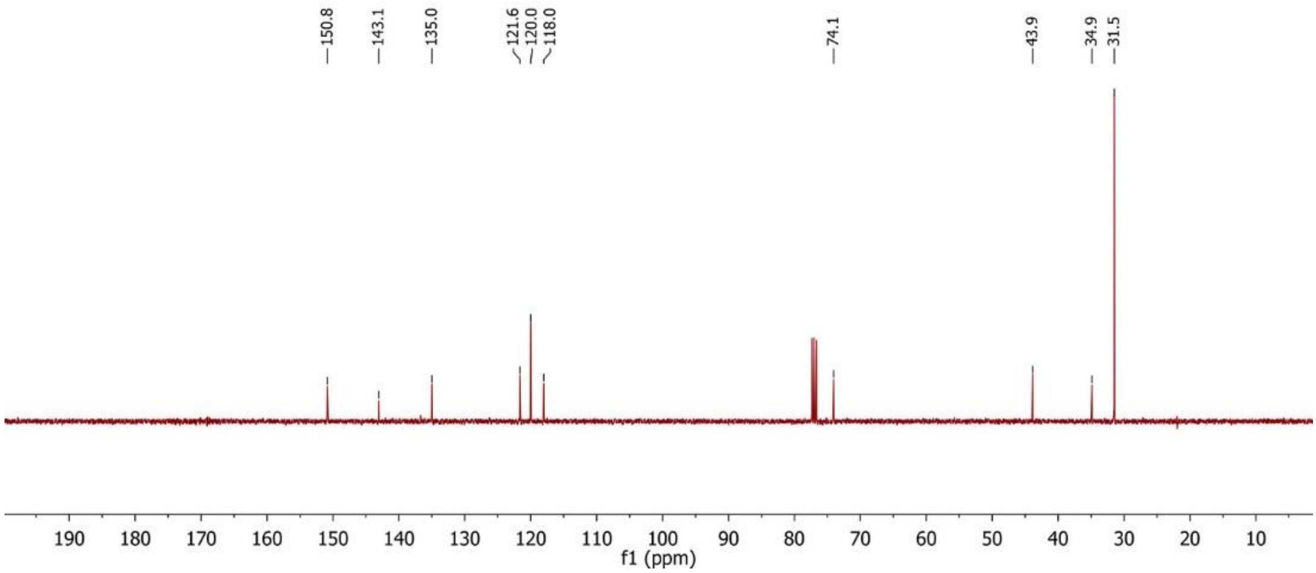

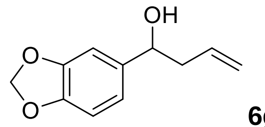

6g

^{1}H NMR (400 MHz, $CDCl_3$)

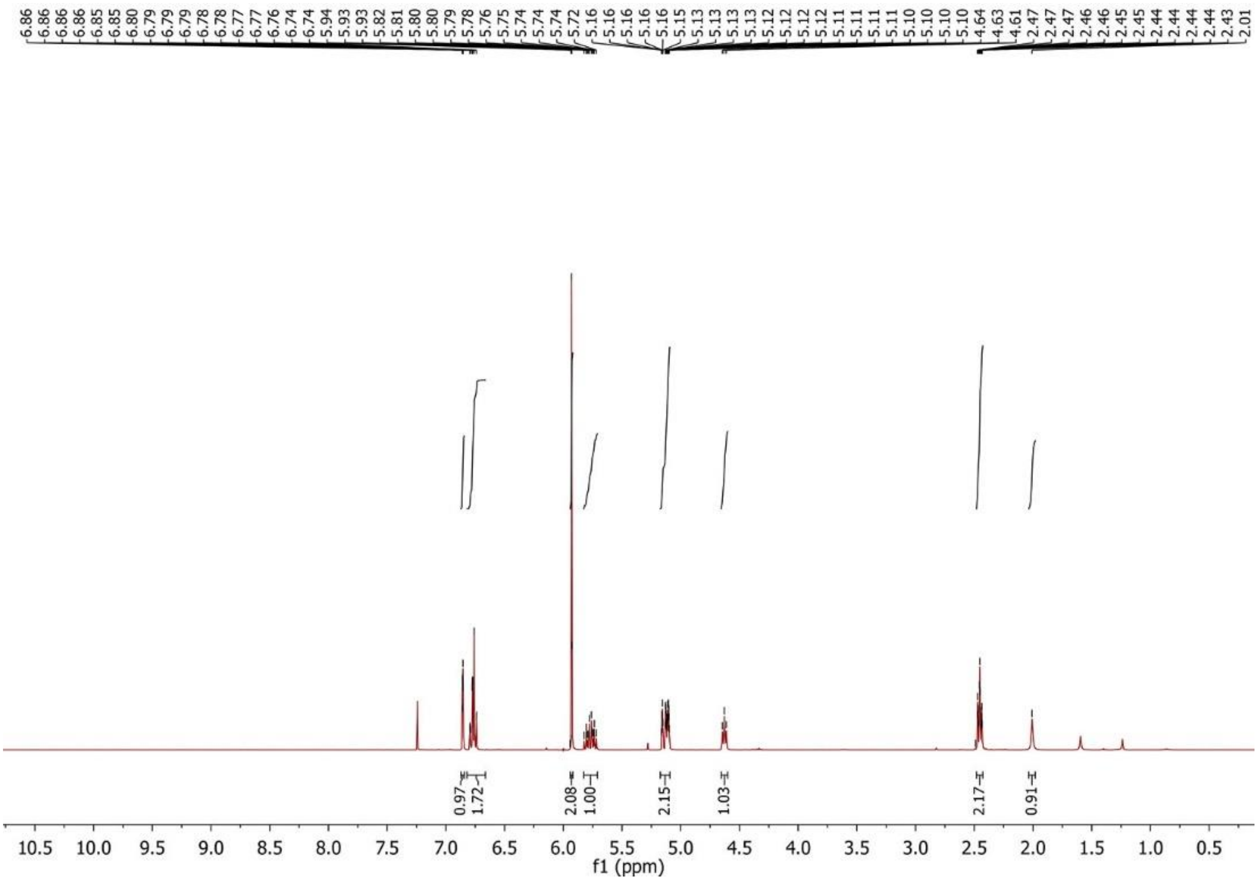

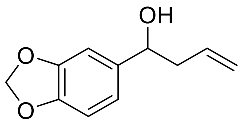

6g

$^{13}C\{^{1}H\}$ NMR (100 MHz, $CDCl_3$)

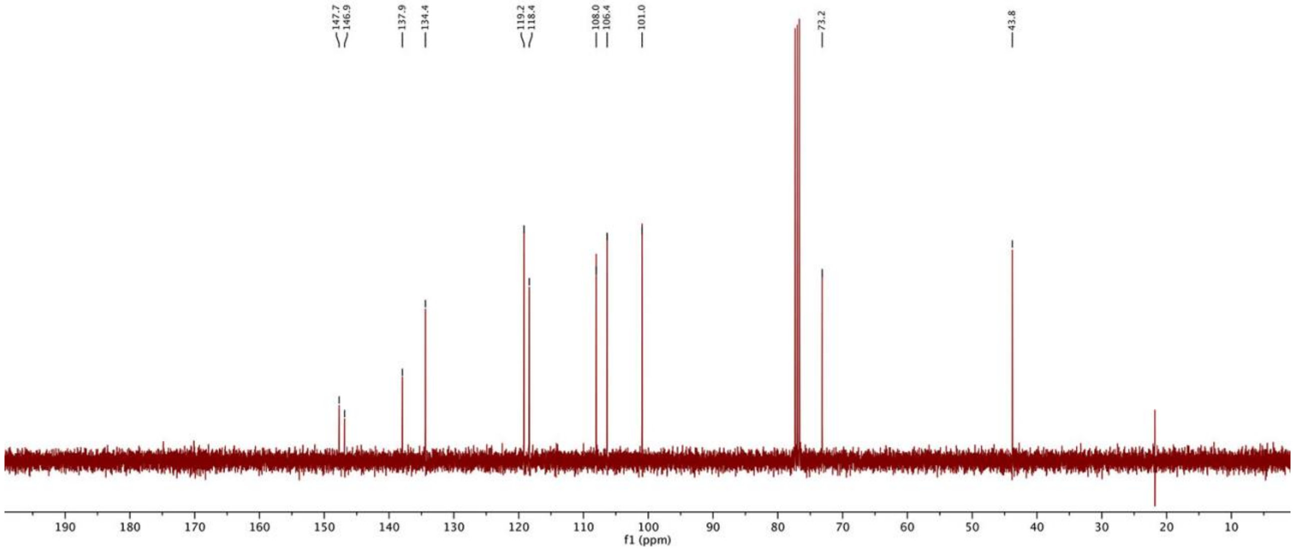

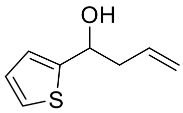

6h

^{1}H NMR (400 MHz, $CDCl_3$)

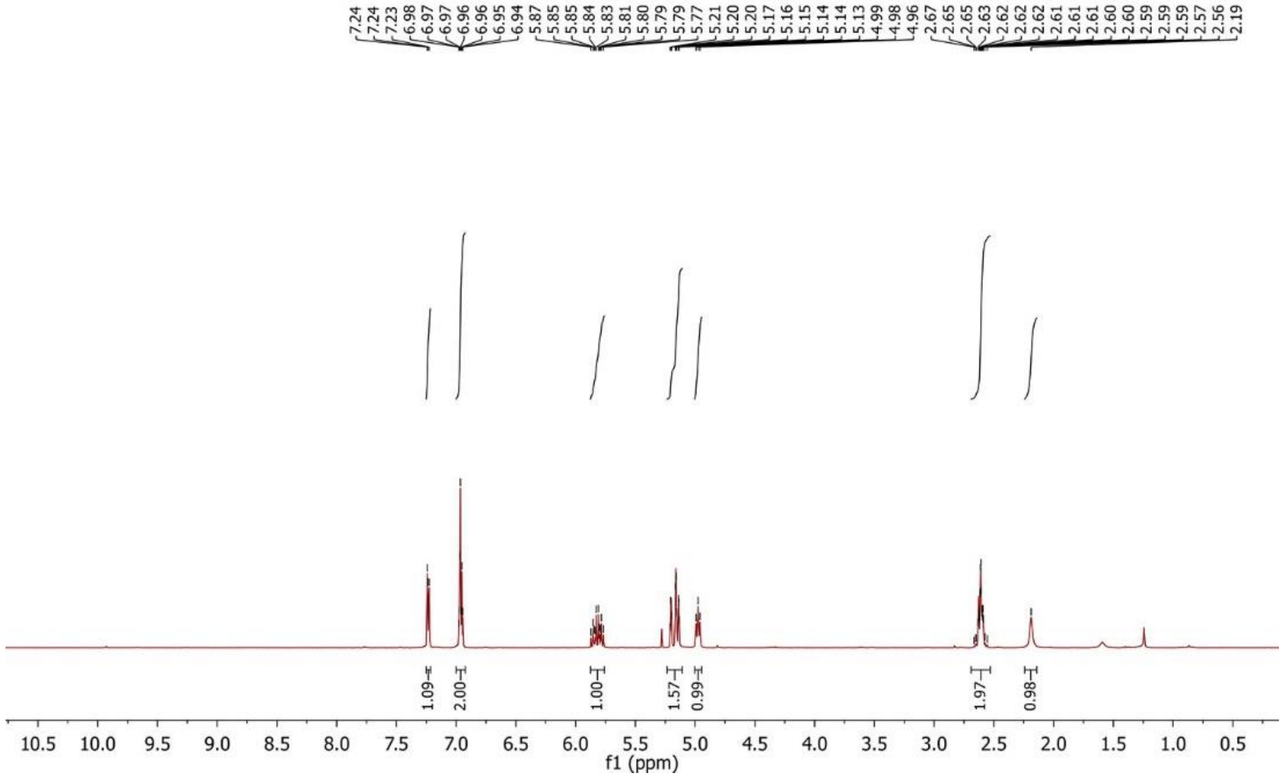

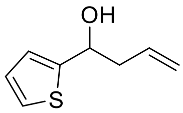

6h

^{13}C{^{1}H} NMR (100 MHz, $CDCl_3$)

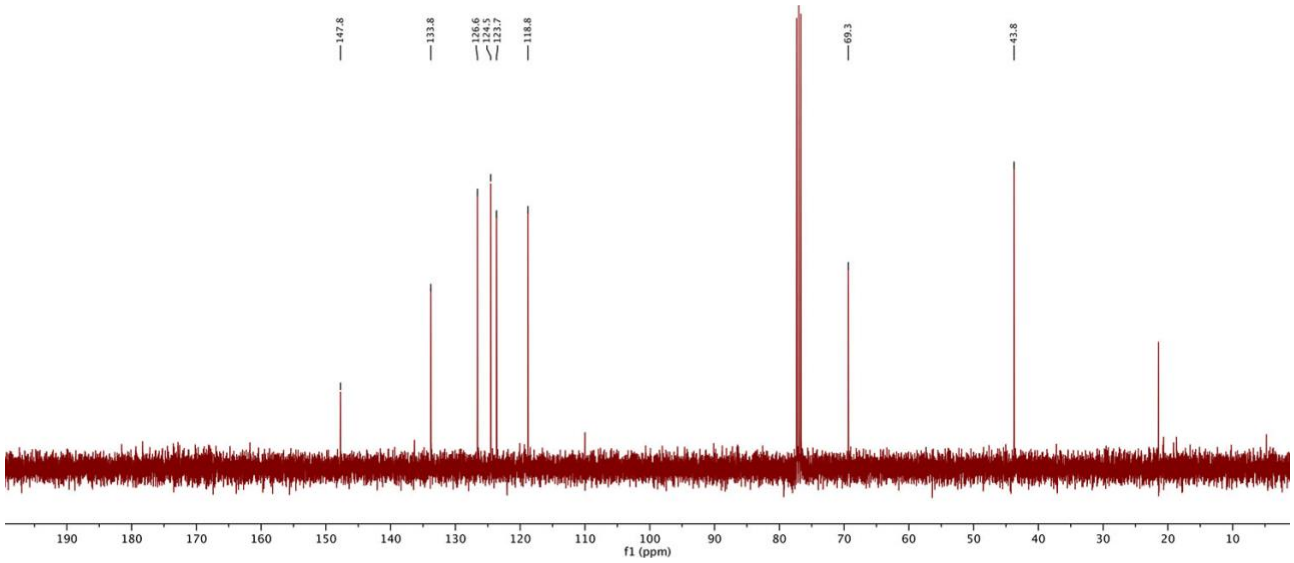

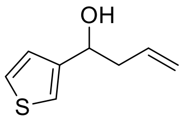

6i

^{1}H NMR (400 MHz, $CDCl_3$)

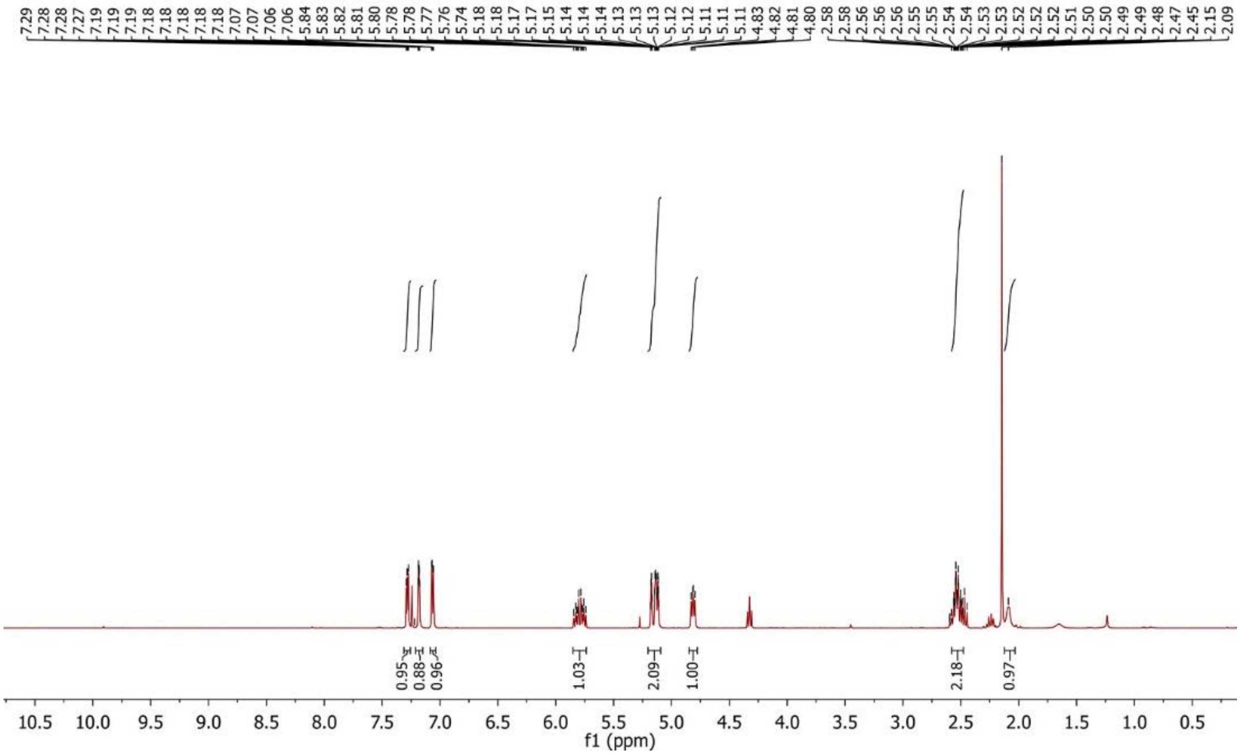

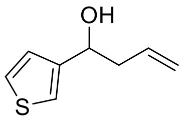

6i

^{13}**C{^{1}H} NMR** (100 MHz, $CDCl_3$)

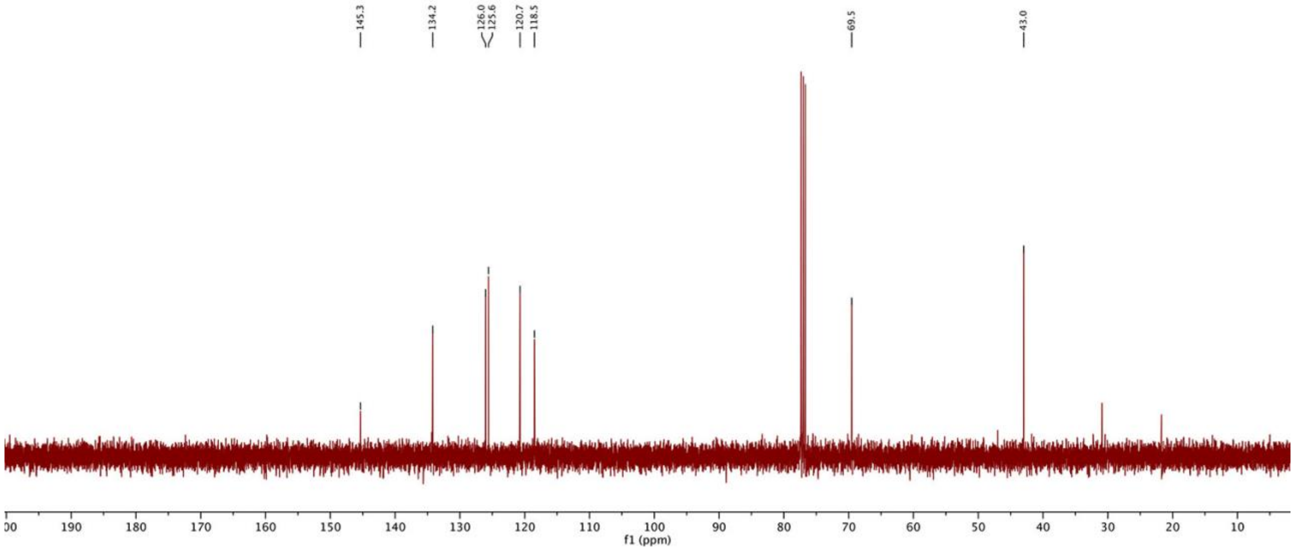

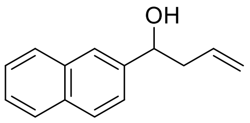

6j

^{1}H NMR (400 MHz, $CDCl_3$)

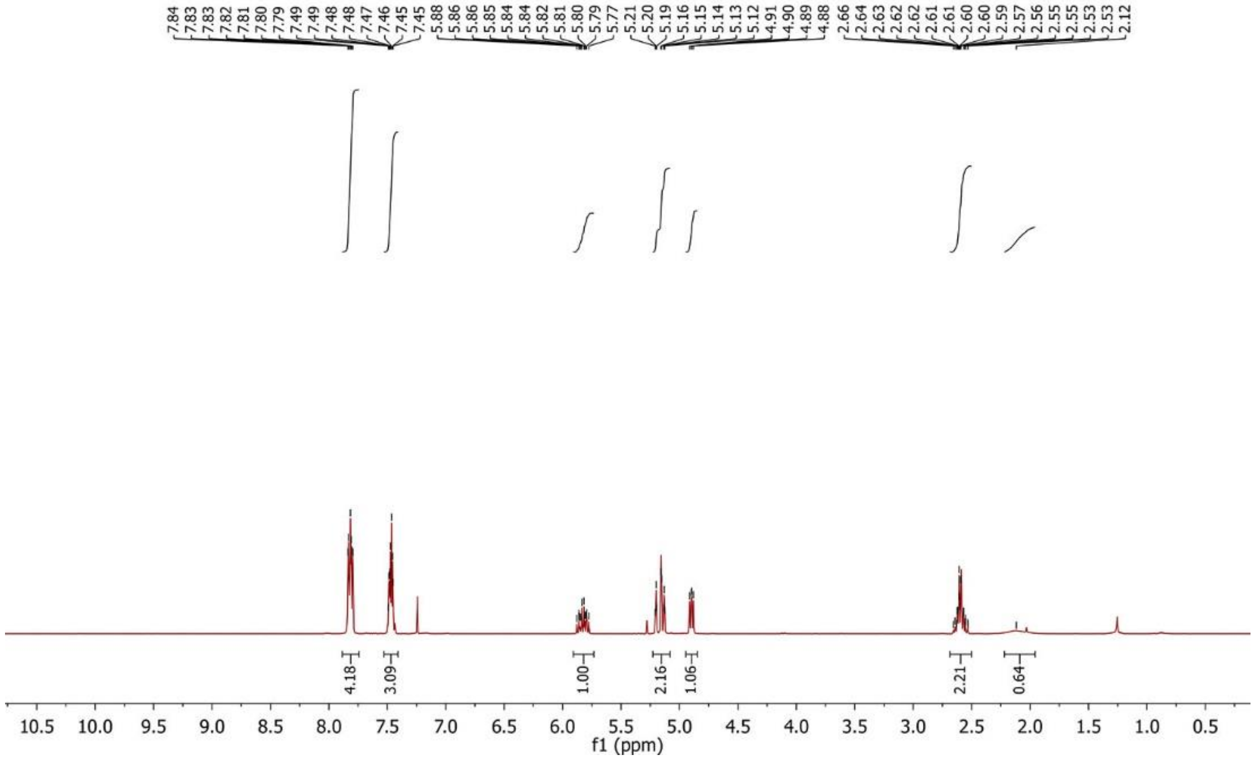

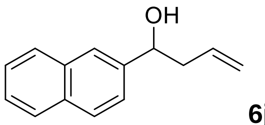

6j

^{13}**C{**^{1}**H} NMR** (100 MHz, $CDCl_3$)

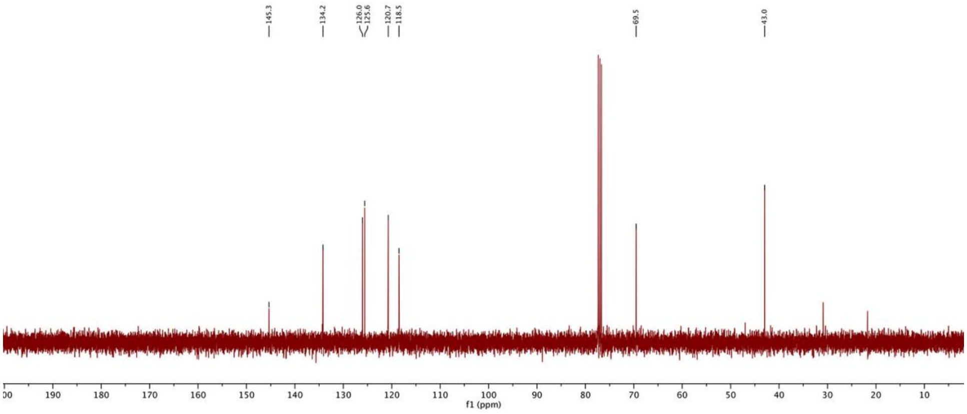

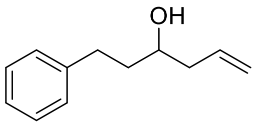

6k

[1]**H NMR** (400 MHz, $CDCl_3$)

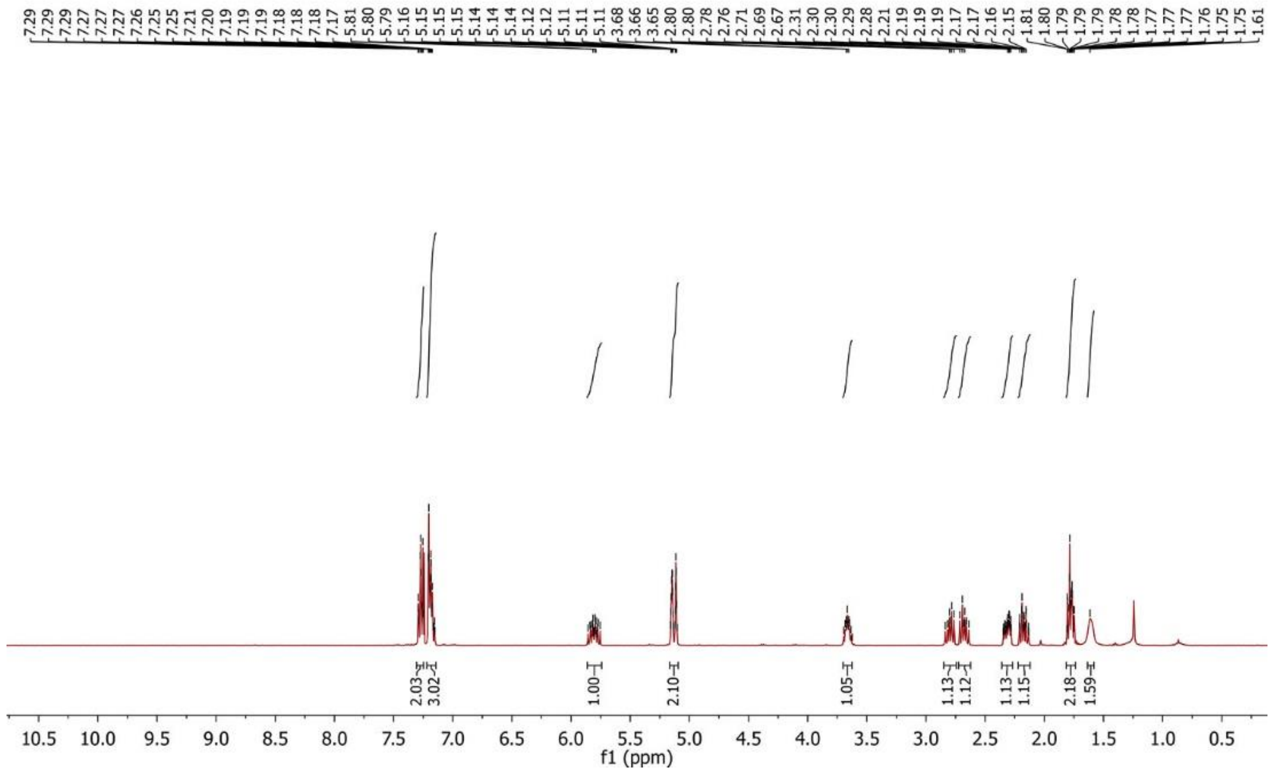

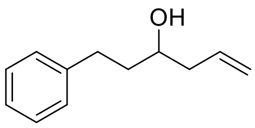

6k

$^{13}C\{^1H\}$ NMR (100 MHz, $CDCl_3$)

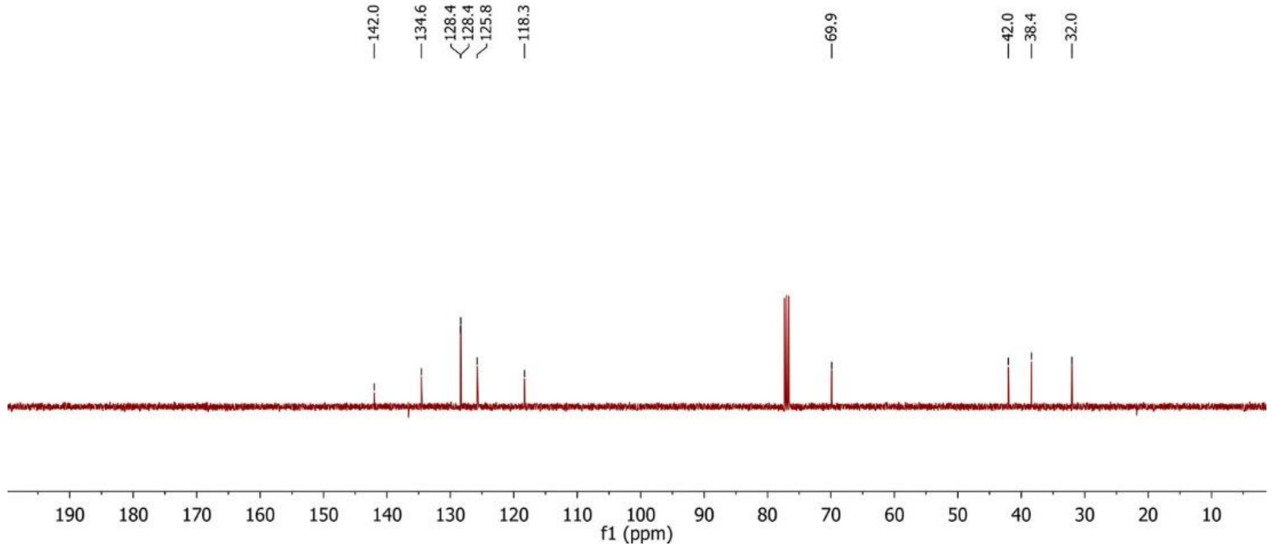

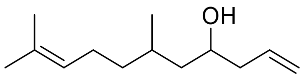

6l

^{1}H NMR (400 MHz, $CDCl_3$)

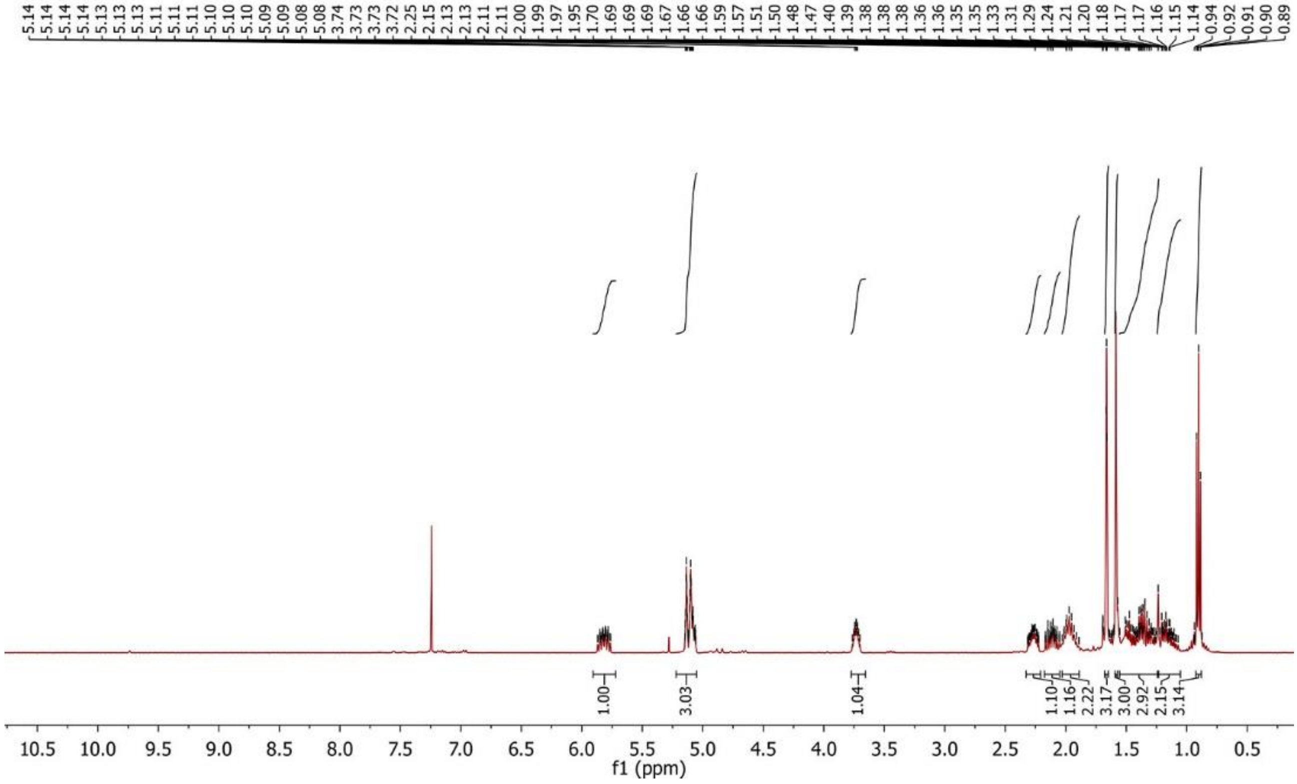

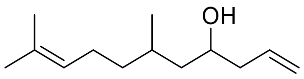

6l

$^{13}C\{^{1}H\}$ NMR (100 MHz, $CDCl_3$)

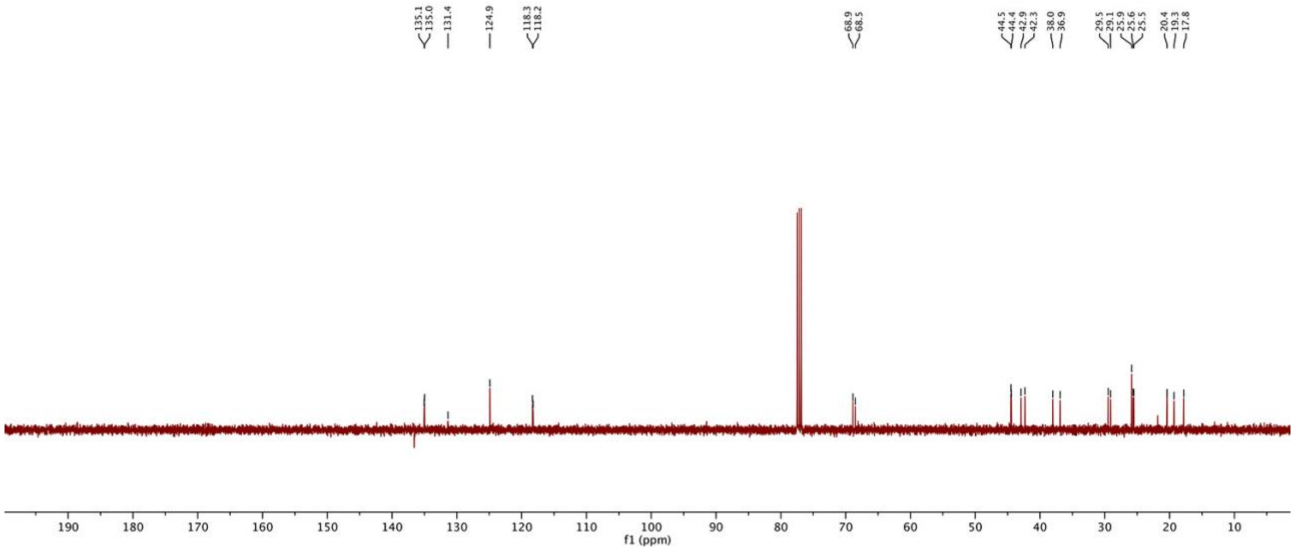

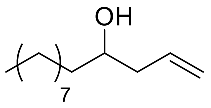

6m

^{1}H NMR (400 MHz, $CDCl_3$)

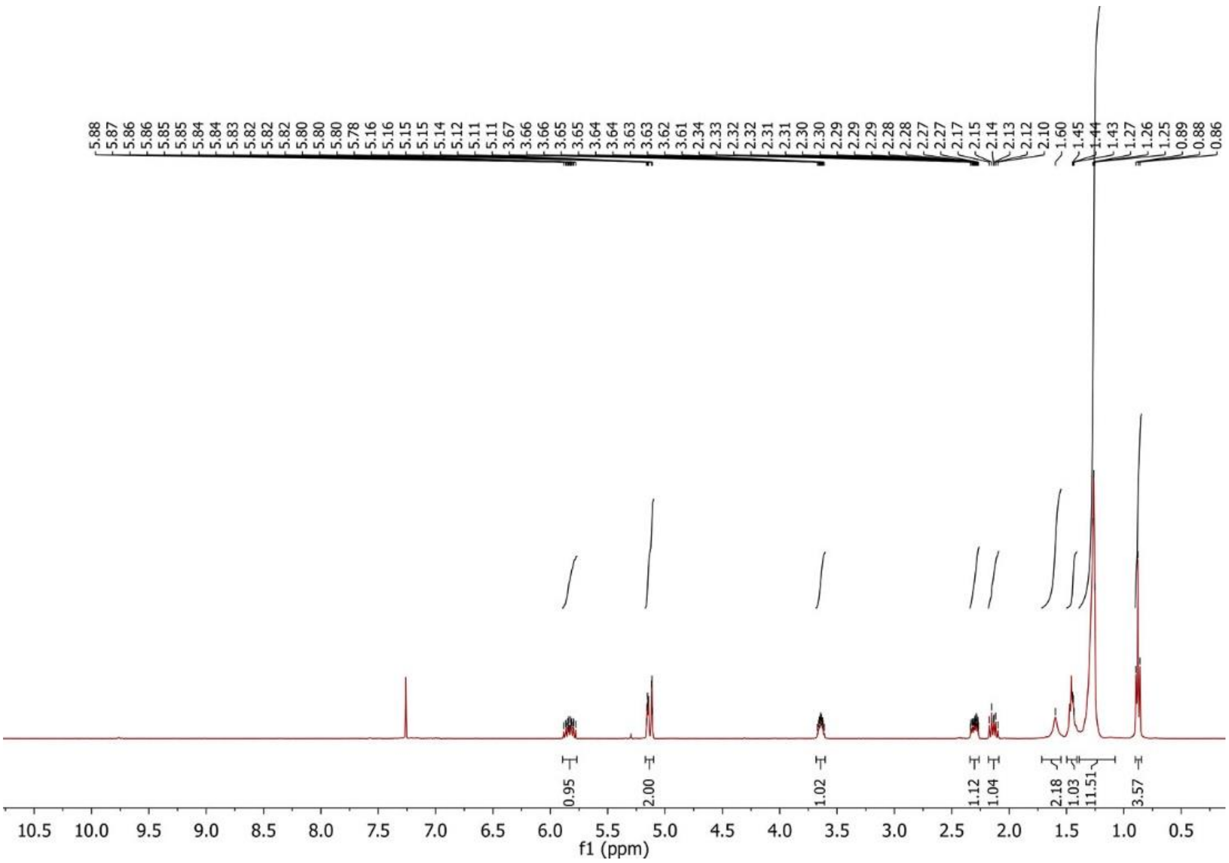

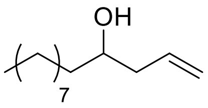

6m

$^{13}C\{^{1}H\}$ NMR (100 MHz, $CDCl_3$)

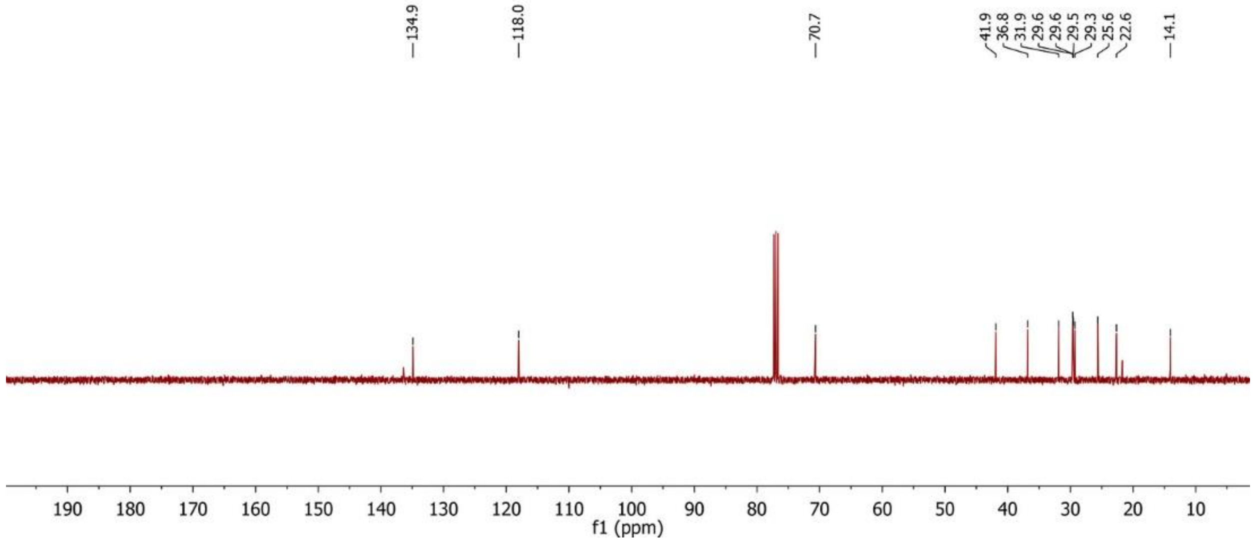

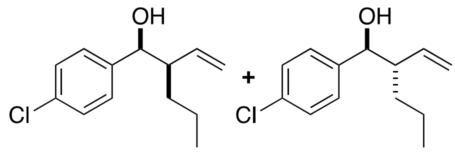

8a

^{1}H NMR (400 MHz, $CDCl_3$)

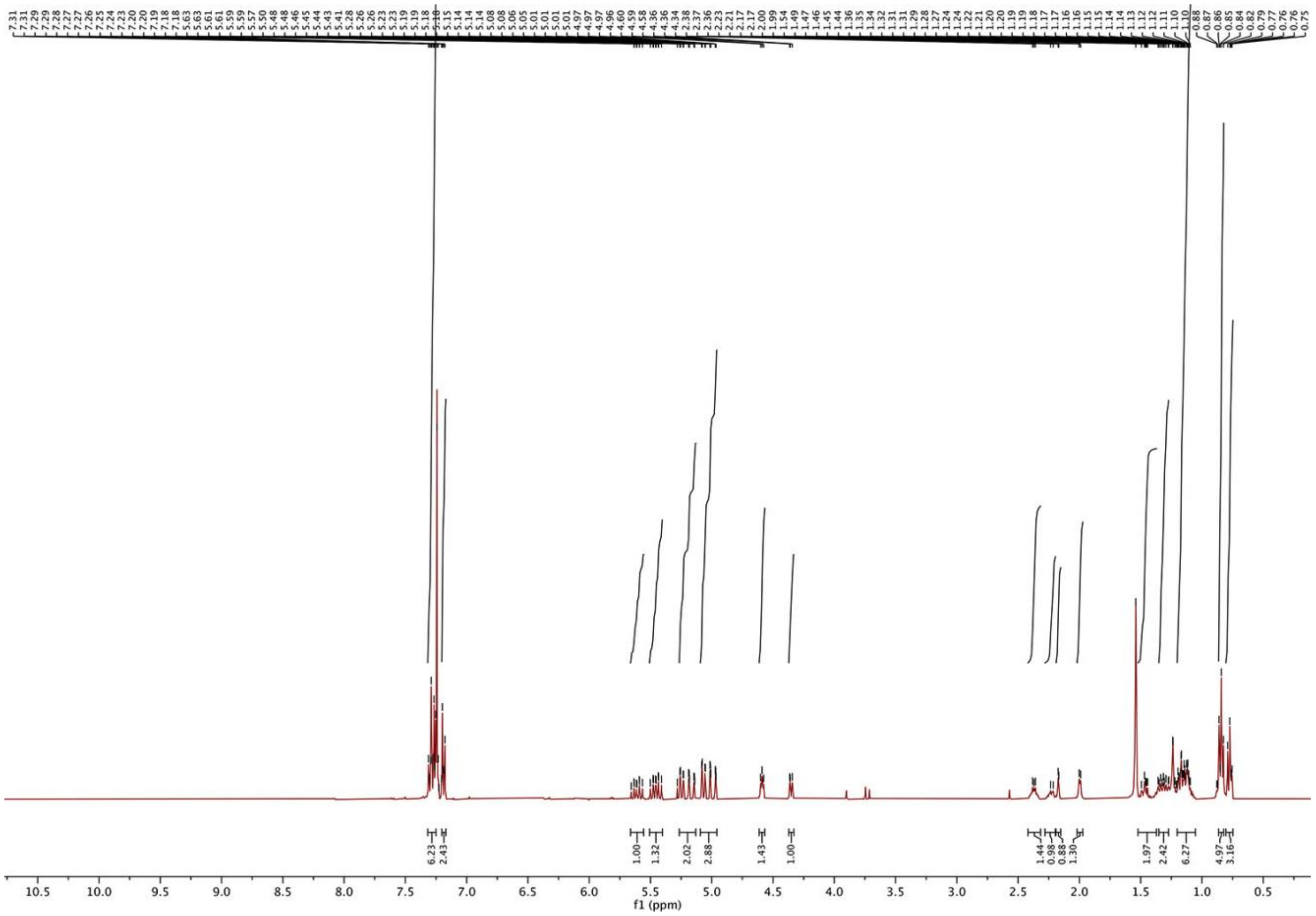

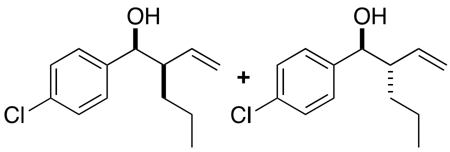

8a

^{13}C{^{1}H} NMR (100 MHz, $CDCl_3$)

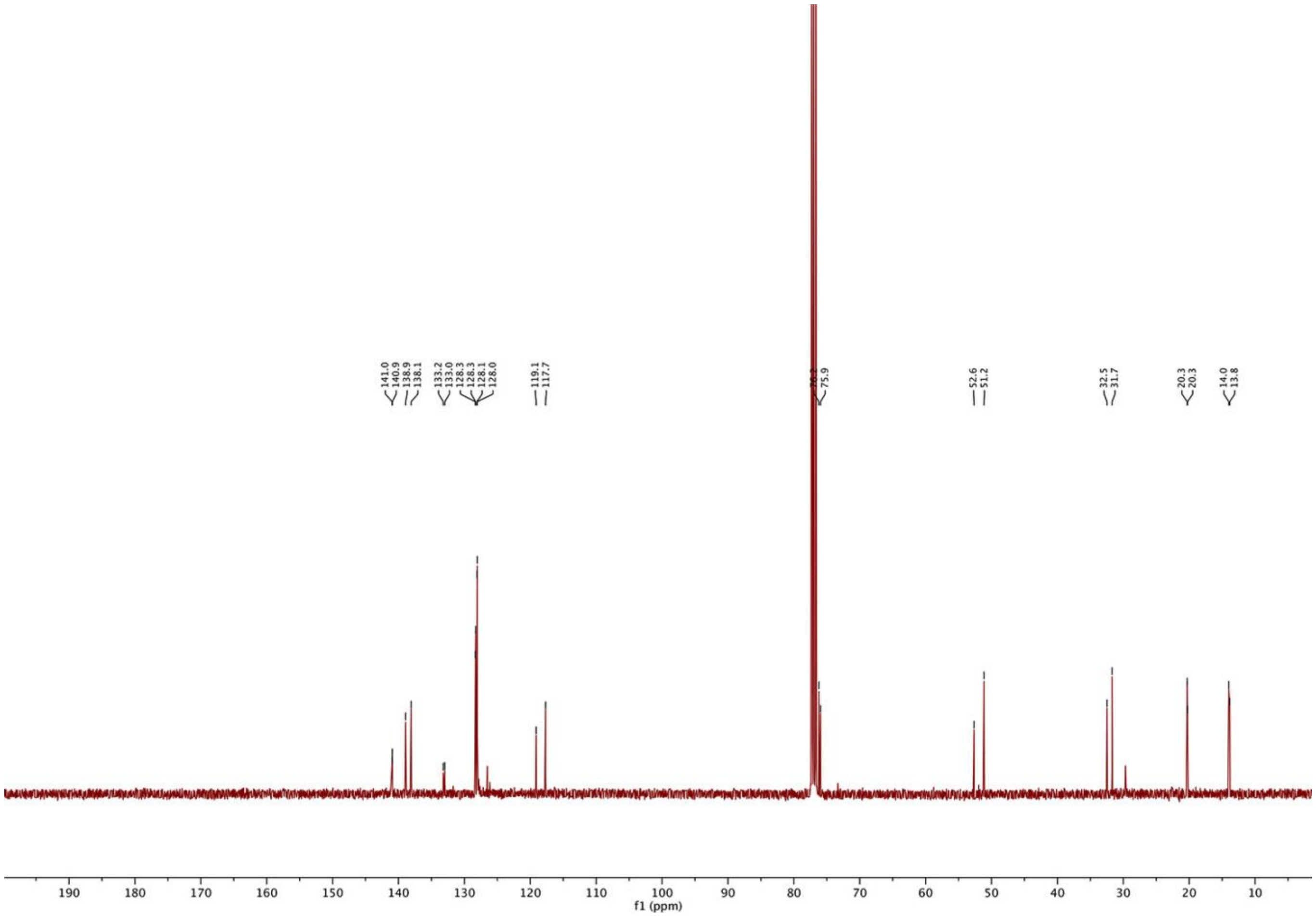

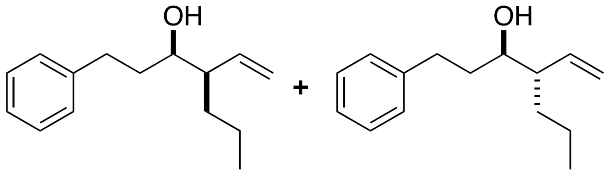

8k

^{1}H NMR (400 MHz, $CDCl_3$)

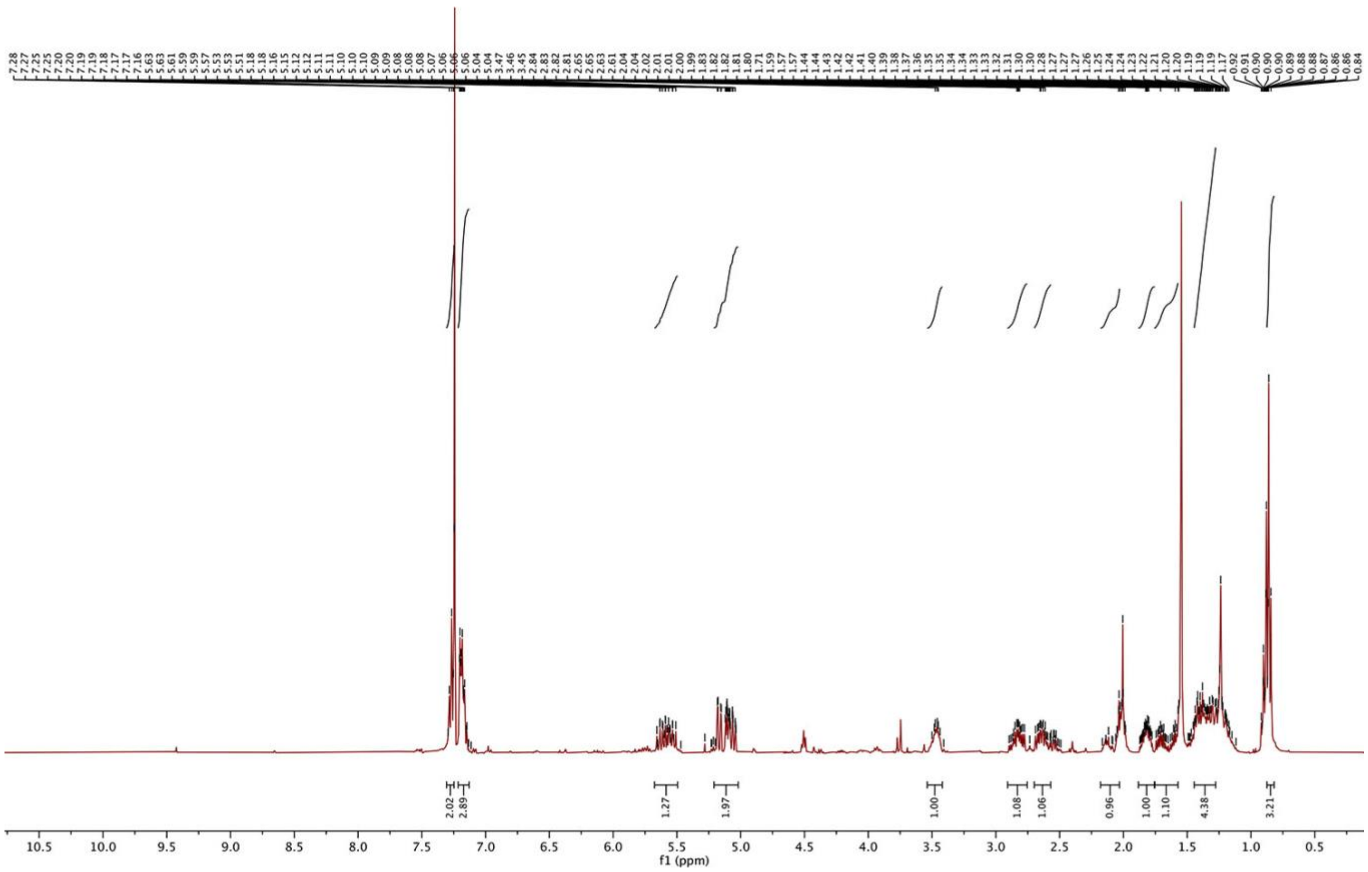

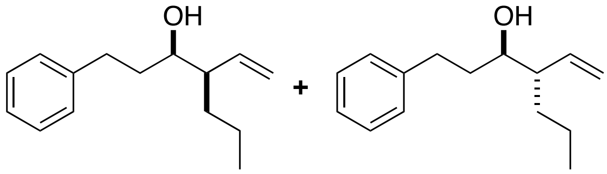

8k

^{13}C{^{1}H} NMR (100 MHz, CDCl$_3$)

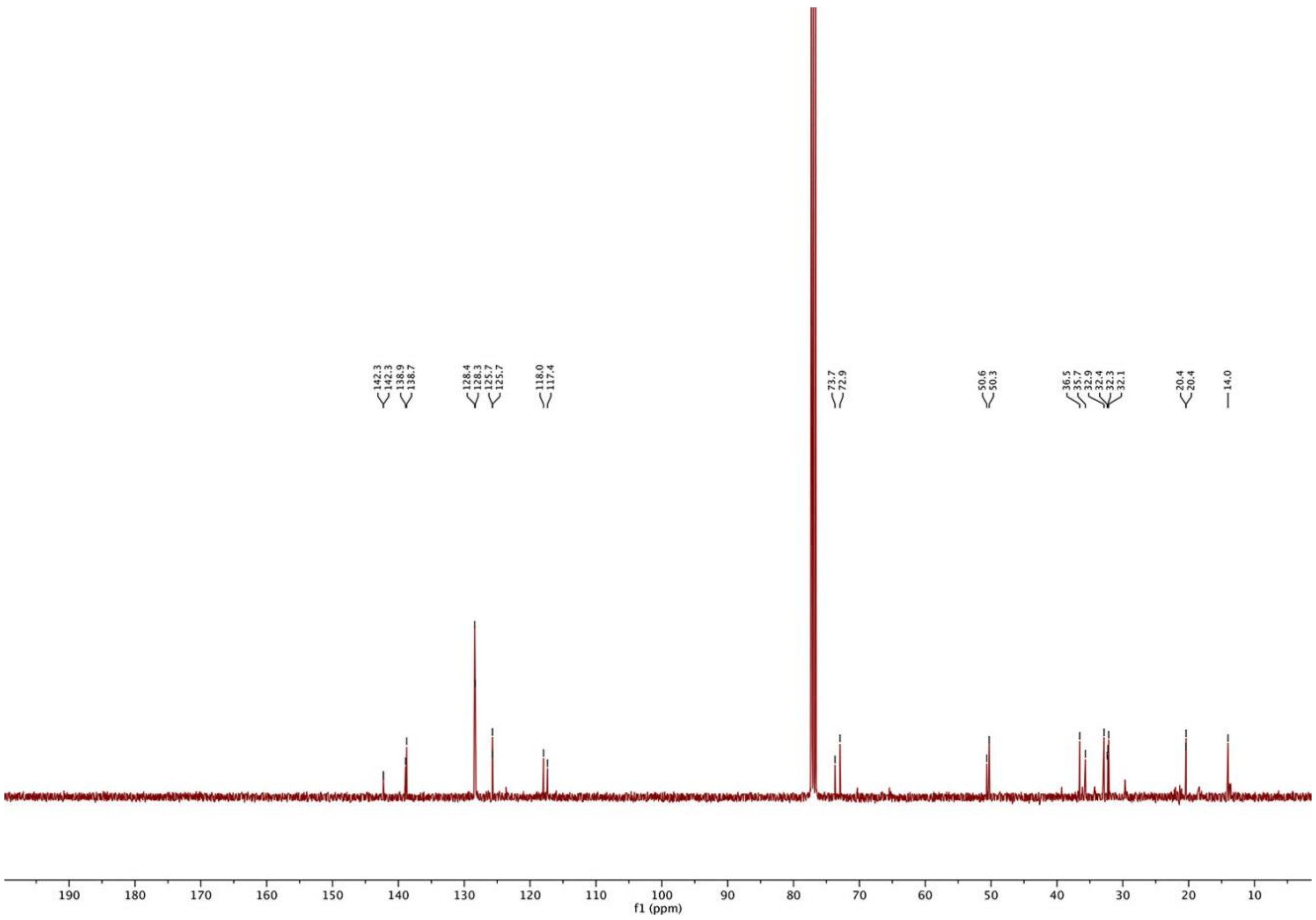

Supplement: Supplementary file 1 — jo2c01825_si_001.pdf [file jo2c01825_si_001.pdf]
